# Placenta-derived macaque trophoblast stem cells: differentiation to syncytiotrophoblasts and extravillous trophoblasts reveals phenotypic reprogramming

**DOI:** 10.1038/s41598-020-76313-w

**Published:** 2020-11-05

**Authors:** Jenna Kropp Schmidt, Logan T. Keding, Lindsey N. Block, Gregory J. Wiepz, Michelle R. Koenig, Michael G. Meyer, Brittany M. Dusek, Kamryn M. Kroner, Mario J. Bertogliat, Avery R. Kallio, Katherine D. Mean, Thaddeus G. Golos

**Affiliations:** 1 Wisconsin National Primate Research Center, University of Wisconsin-Madison, Madison, WI USA; 2 Department of Comparative Biosciences, University of Wisconsin-Madison, Madison, WI USA; 3 Department of Obstetrics and Gynecology, University of Wisconsin-Madison, Madison, WI USA

**Keywords:** Stem cells, Rhesus macaque

## Abstract

Nonhuman primates are excellent models for studying human placentation as experimental manipulations in vitro can be translated to in vivo pregnancy. Our objective was to develop macaque trophoblast stem cells (TSCs) as an in vitro platform for future assessment of primate trophoblast development and function. Macaque TSC lines were generated by isolating first and second trimester placental villous cytotrophoblasts followed by culture in TSC medium to maintain cellular proliferation. TSCs grew as mononuclear colonies, whereas upon induction of syncytiotrophoblast (ST) differentiation multinuclear structures appeared, indicative of syncytium formation. Chorionic gonadotropin secretion was > 4000-fold higher in ST culture media compared to TSC media. The secretion of chorionic gonadotropin by TSC-derived ST reflects a reprogramming of macaque TSCs to an earlier pregnancy phenotype. Characteristic trophoblast hallmarks were defined in TSCs and ST including expression of C19MC miRNAs and the macaque placental nonclassical MHC class I molecule, Mamu-AG. Extravillous trophoblasts (EVTs) were derived that express macaque EVT markers Mamu-AG and CD56, and also secrete high levels of MMP2. Our analyses of macaque TSCs suggests that these cells represent a proliferative, self-renewing population capable of differentiating to STs and EVTs in vitro thereby establishing an experimental model of primate placentation.

## Introduction

Pregnancy-related complications, including preeclampsia and intrauterine fetal growth restriction, stem from suboptimal placental development^1,2^. The consequences of poor placental development transcend pregnancy as fetal programming in utero predisposes the fetus to increased risk for cardiovascular disease and metabolic syndrome in adulthood^3,4^. The molecular cues underlying placental developmental events occurring within the first trimester of human pregnancy, however, are not well understood. Therefore, defining the mechanisms underlying early placentation is necessary to not only improve both maternal and fetal well-being, but also to improve the child’s health throughout their lifespan.

Early placental development in humans is relatively unexplored as in vivo samples are a limited resource. It is postulated that a trophoblast stem cell (TSC) niche resides in the early human first trimester placenta^5–8^, where these cells are a proliferative, less differentiated progenitor cell population that gives rise to both villous cytotrophoblasts that fuse to form the syncytia as well as extravillous trophoblast (EVT) progenitor cells at the cell column tips^9^. The isolation and characterization of human TSC populations has been limited given the uncertainty of which markers truly define primitive or early trophoblasts and TSCs^5^.

Trophoblast culture systems serve as in vitro platforms to develop a better understanding of placental development and physiology as environmental/culture conditions and genes can be manipulated. Human trophoblast cell models include cells derived from naturally occurring choriocarcinomas (AC1M, BeWo, JAR, JEG-3)^10–12^, immortalization of human primary trophoblast cultures (HTR-8/SVneo, SWAN-71)^10,13,14^, treatment of human pluripotent embryonic stem cells (ESCs) to promote specification of trophectoderm^15,16^, and the isolation of primary trophoblasts^17^. Human ESCs differentiated to trophoblast-like cells upon BMP4 treatment^15,16^, however, do not continue to proliferate. There is also considerable variation in BMP4 treatment protocols leading to differing gene expression profiles and phenotypes^5,18^. Moreover, ESC-derived trophoblasts differ from villous cytotrophoblasts in their gene expression profile, which is representative of a more primitive trophoblast cell^18^.

In the mouse, TSCs can be derived by culturing extraembryonic ectoderm in the presence of FGF4, heparin and mouse embryonic fibroblasts (or supplementation of TGF-ß1/Activin) and can readily serve as an in vitro experimental platform^19^. However, these methods have not been successfully adapted to human TSC derivation^20^, suggestive that FGF4 is dispensable for human TSC renewal. More recently, optimization of culture conditions for human trophoblasts has led to the establishment of human TSCs and TSC-organoid models^21–23^. Okae et al.^21^ defined a TSC medium capable of supporting continued proliferation of primary human cytotrophoblasts and blastocyst-derived trophoblasts when cultured on a collagen IV matrix. The human TSCs expressed characteristic trophoblast transcription factors and primate-specific chromosome 19 microRNA cluster (C19MC) miRNAs and also demonstrated DNA hypomethylation near the *ELF5* promoter, all of which are features of human trophoblasts. Importantly, under differentiation-specific culture media formulations TSCs could be differentiated to either chorionic gonadotropin (CG)-secreting syncytia or HLA-G positive EVTs. The derivation of human trophoblast organoid cultures was recently described by Haider et al.^22^ and Turco et al.^23^ In both studies, the organoids formed mononuclear trophoblasts on the outer periphery with syncytia and lacunae-like structures within the center. Upon modulation of culture media conditions in either organoid system, HLA-G positive trophoblastic outgrowths formed, a hallmark of human EVTs. Although the human TSC media components vary slightly across these studies, collectively they have demonstrated that Wnt activation, EGF signaling, and inhibition of TGF-ß are essential for maintaining proliferation of human trophoblasts in culture.

Macaques are an ideal model for human pregnancy studies as, like the human, they develop a villous hemochorial placenta^24,25^. Primate placentation is characterized by invasion of trophoblasts into the decidualized endometrium and remodeling of maternal spiral arteries. Importantly, macaques express placenta-specific MHC class I homologs^26–29^ and C19MC miRNAs^30^ similar to humans. The macaque model uniquely presents an experimental continuum utilizing in vitro embryos, in vitro trophoblast cell cultures and experimental in vivo pregnancy studies to encompass each stage of pregnancy.

Previously reported macaque in vitro trophoblast models have been derived by extended culture of hatched blastocysts on feeder layers or with feeder layer-conditioned media^31–34^. Matsumoto et al.^34^ recently derived macaque TSCs by extended culture of blastocysts in the presence of FGF4, the key factor in maintaining mouse TSCs. While Matsumoto et al.^34^ and VandeVoort et al.^33^ have provided evidence of in vitro macaque trophoblast differentiation, these TSC models lack the ability to tightly control differentiation in a cell-type specific manner. Hence, our objective was to derive macaque TSCs utilizing the method described by Okae et al.^21^ to generate human TSCs, where differentiation was tightly controlled for deriving either STs or EVTs. In this study, eight macaque TSC lines were generated from first trimester and early second trimester placental villous cytotrophoblasts (pri-CTB), a more widely available resource in comparison to monkey blastocysts. Here we demonstrate that macaque TSCs are capable of maintaining cellular proliferation in vitro, and upon appropriate culture conditions, can differentiate to both ST and EVT-like cells. Importantly, macaque-derived TSCs and their differentiated derivatives display characteristic features of human trophoblasts, and remarkably, reestablish CG secretion indicating reversion to an early pregnancy phenotype. The macaque TSC model offers an experimental platform for in vitro assessment of experimental infection, evaluation of trophoblast-targeted therapies, and development of genome editing tools to assess primate trophoblast development and function for translation to in vivo macaque pregnancy studies.

## Results

### Generation of TSCs and differentiated trophoblast cells

**Figure 1:**
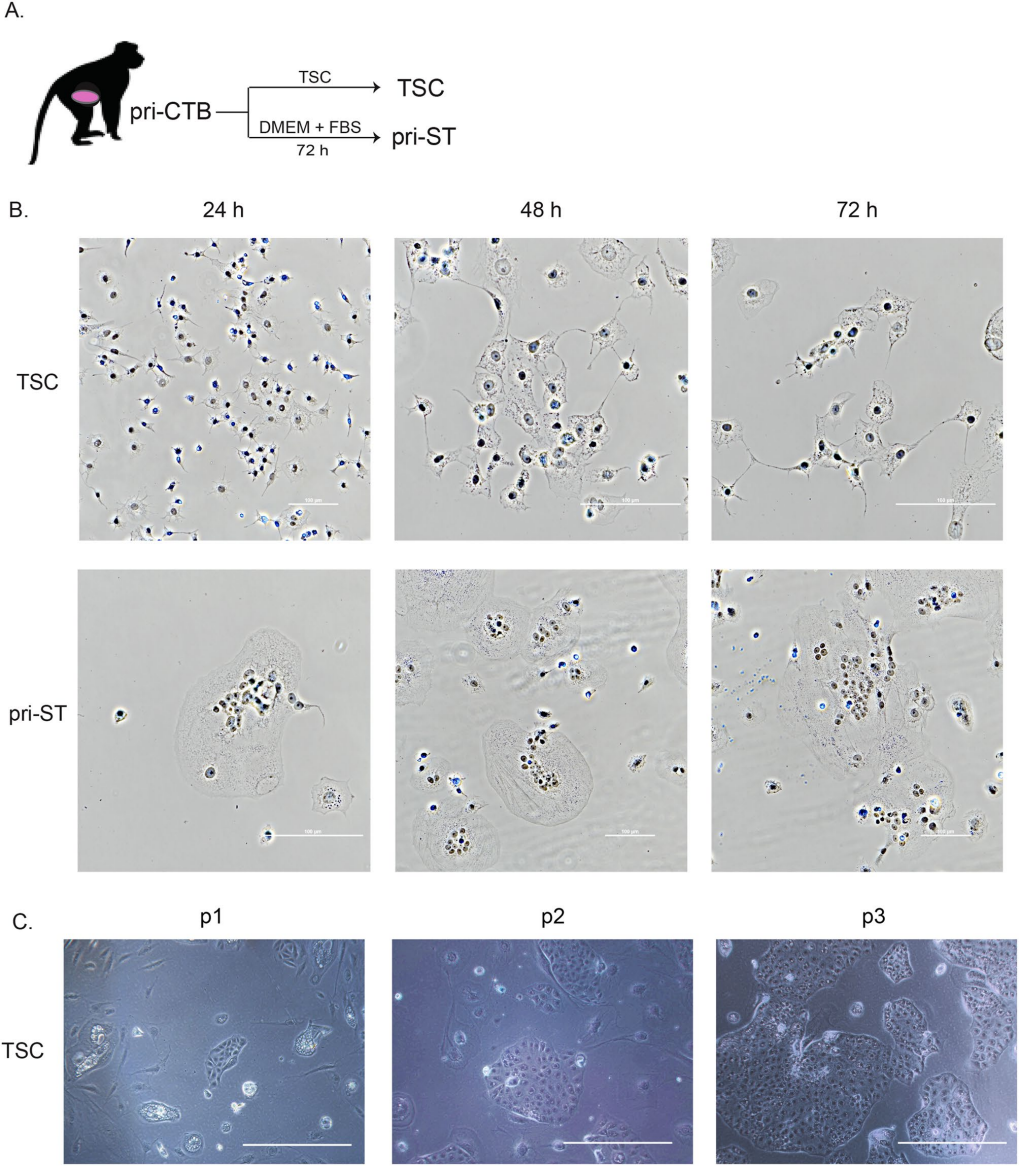
Derivation of trophoblast stem cells and primary syncytiotrophoblasts from villous cytotrophoblasts. (**A**) Primary villous cytotrophoblasts (pri-CTB) were isolated from macaque placentas and cultured in trophoblast stem cell (TSC) medium to support cellular proliferation or differentiated to primary syncytiotrophoblasts (pri-ST). (**B**) Phase contrast images of TSC and pri-ST at 24, 48 and 72 h in culture. Scale bars represent 100 µm. (**C**) Representative phase-contrast microscopic images of TSCs at passage (p) 1, 2 and 3. Scale bars represent 500 µm.

Placentas were collected between 40–75 days of gestation from eight pregnant macaques to isolate pri-CTB for the generation of self-renewing TSCs, as illustrated in Fig. 1A. Pri-CTBs grown in standard trophoblast culture medium (DMEM and 10% FBS) readily formed syncytia within 72 h and did not continue to proliferate (Supplementary Fig. S1), as has been previously shown^35,36^. In contrast, initial TSC cultures contained few syncytia (Fig. 1B), and mononuclear cell colonies of relatively homogeneous appearance were clearly visible within the first week of culture in TSC medium. TSC lines typically proliferated rapidly with passage at 2–5 day intervals. Representative images from the first three passages are shown in Fig. 1C.

**Figure 2:**
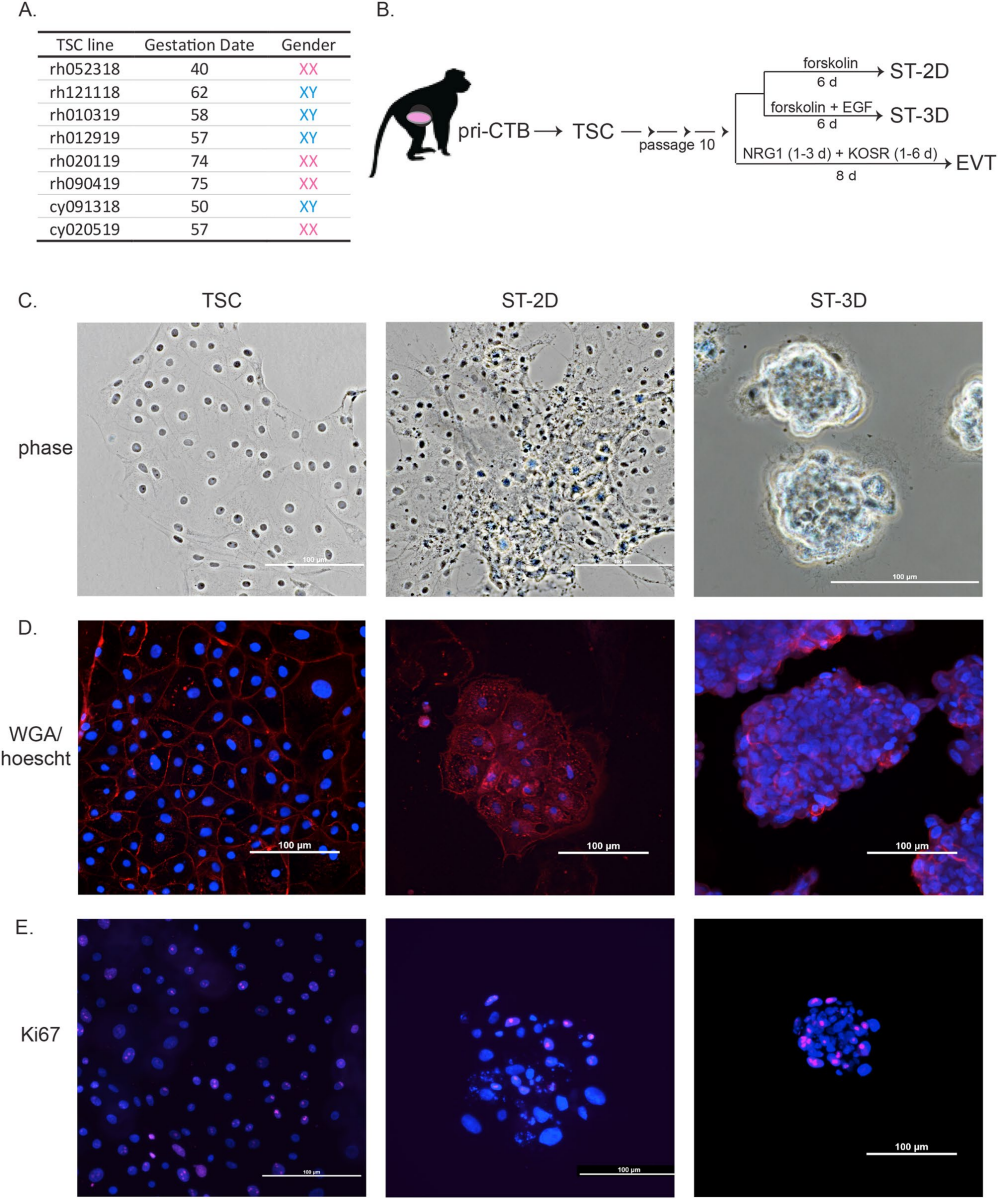
Generation of macaque TSCs and TSC-derived syncytiotrophoblasts. (**A**) Summary of eight TSC lines derived from first or early second trimester rhesus (rh) or cynomolgus (cy) macaque placentas. (**B**) TSCs underwent controlled differentiation beginning at passage 10. Treatment for directing TSCs to adherent 2-dimensional syncytiotrophoblasts (ST-2D), 3-dimensional syncytiotrophoblasts (ST-3D) cultured in suspension, or adherent extravillous cytotrophoblasts (EVT) are outlined. (**C**) Representative phase-contrast microscopic images of each cell type. (**D**) Cells stained with wheat-germ agglutinin (WGA, red) to display cell membranes and Hoescht 33342 (blue) to visualize nuclei. (**E**) Cell proliferation depicted by Ki-67 immunostaining (punctate pink staining) and nuclei stained by DAPI (blue). Scale bars in all panels represent 100 µm.

Putative TSCs were derived from six rhesus and two cynomolgus macaque pri-CTB isolations, of which, four each were of XY and XX karyotype (Fig. 2A). Normal karyotypes were observed in four lines, while an abnormal karyotype was observed in the other four lines with a subset of cells being polyploid (Supplementary Table S1). Chromosome integrity may reflect mosaicism present in the initial cells as has been observed in cytogenetic analysis of human chorionic villi^37^, or could be incurred as a result of culture and continued passage. TSC lines have been cultured up to 50 passages with consistent mononuclear TSC-like morphologies, but comprehensive characterization of karyotype and marker expression of later passages has not yet been performed.

Trophoblast differentiation was performed after the TSC cultures reached passage 10 as was described for human TSCs^21^. TSCs were differentiated to syncytiotrophoblasts (ST) in 2-dimensional (ST-2D) and 3-dimensional (ST-3D) paradigms by either plating TSCs as adherent cultures on collagen IV (col IV)-coated dishes or by culturing in suspension, respectively (Fig. 2B). Adherent ST-2D cells displayed a more raised structure compared to TSCs, with the presence of both multinuclear and mononuclear cell regions indicative of incomplete syncytialization (Fig. 2C). TSCs cultured in suspension with ST medium (ST-3D) largely formed multinuclear aggregates that increased in size as cells aggregated throughout the 6 day differentiation regimen. The presence of syncytia was confirmed by staining the cell membrane with wheat germ agglutinin (WGA) and Hoescht 33342 to visualize the nuclei (Fig. 2D). WGA staining clearly distinguished individual mononuclear TSCs, whereas ST-3D aggregates exhibited multinuclear structures with centrally clustered nuclei. TSCs continually proliferated in culture, as demonstrated by widespread Ki-67 positive staining, a macaque trophoblast marker of cellular proliferation^38^, observed in nearly all TSCs (Fig. 2E). The presence of Ki-67 positive mononuclear cells within ST-2D cultures further suggested that the ST-2D culture paradigm only partially commits cells to differentiate, whereas the ST-3D aggregates displayed fewer Ki-67 positive cells that were predominantly located at the periphery of the aggregate.

### TSCs and differentiated trophoblasts express trophoblast genes

**Figure 3:**
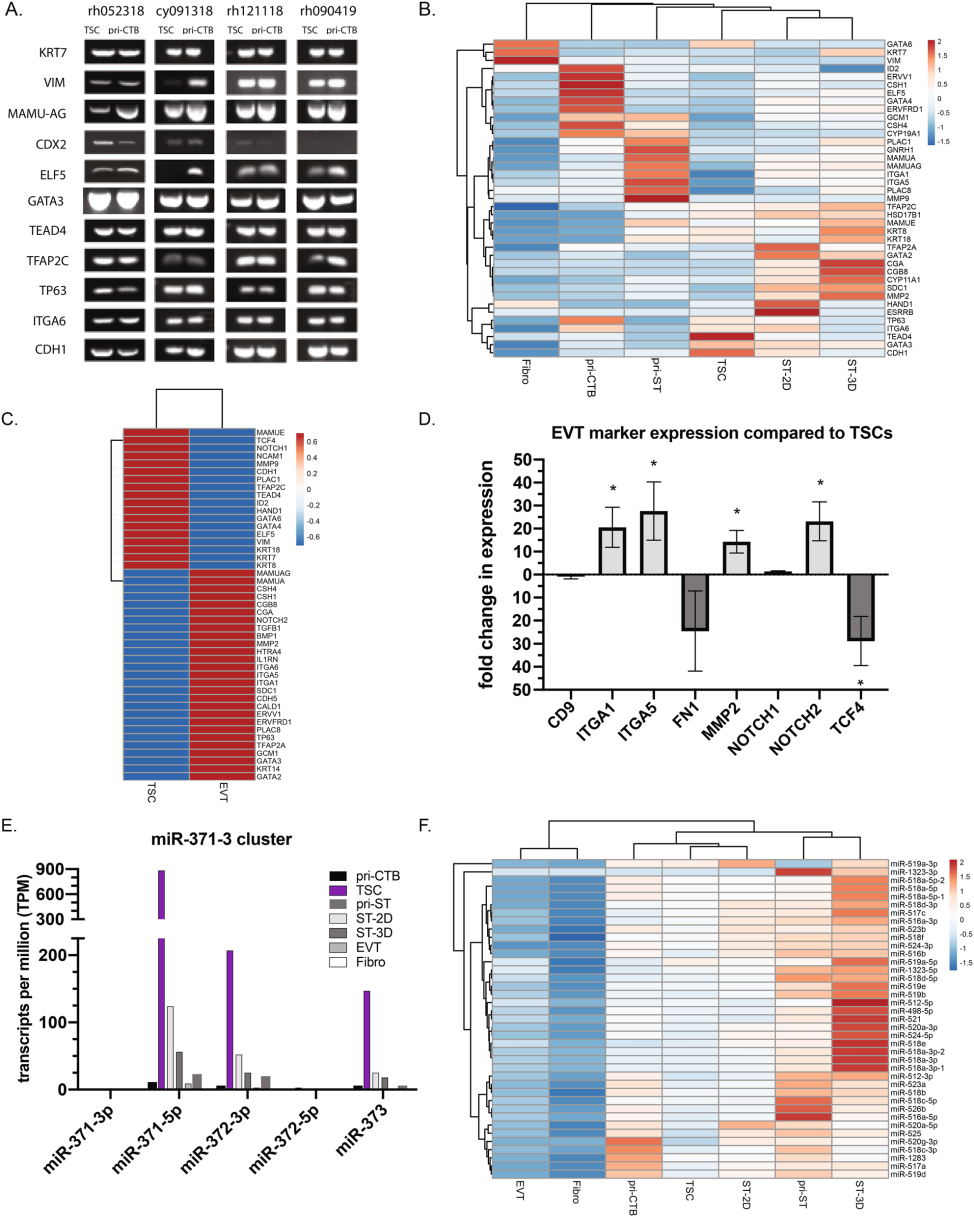
Expression of trophoblast marker mRNAs and miRNAs in parental primary villous cytotrophoblasts, reprogrammed TSC, and differentiated derivatives of TSC. (**A**) RT-PCR products for trophoblast markers in four TSC lines versus isolated pri-CTB. (**B**) RNA-seq gene expression profile of primary cells and fibroblasts compared to derived rh121118 trophoblasts (n = 1 replicate per cell type). For panels, (**B**), (**C**) and (**F**) the rows are mapped by correlation and columns by Euclidean distance and the number of transcripts per million is scaled across rows with the red portion of the color scale bar representing higher values and blue showing lower values. (**C**) RNA-seq gene expression profile of rh121118 TSCs compared to TSC-derived EVTs (n = 1 replicate per cell type). (**D**) Fold change in gene expression of EVTs compared to TSCs. Asterisk denotes a significant difference of p < 0.05 in expression between EVTs and TSCs with genes above the x-axis being more highly expressed in EVTs and those below the x-axis higher in TSCs. (**E**) miR-seq expression profile represented by transcripts per million (TPM) of miR-371-3 cluster miRNAs. (**F**) miR-seq expression profile of chromosome 19 microRNA cluster (C19MC) miRNAs of primary cells and fibroblasts compared to derived rh121118 trophoblasts (n = 1 replicate per cell type).

Classic trophoblast gene expression markers were evaluated for their presence in TSCs and pri-CTB by RT-PCR (n = 4 cell lines). Both pri-CTB and TSCs expressed the hallmark epithelial trophoblast gene, *KRT7*, as well as transcription factors *GATA3*, *TEAD4*, *TFAP2C*, and *TP63* (Fig. 3A). The expression of *VIM*, a mesenchymal marker expected to be absent in trophoblast epithelial cells, was observed in pri-CTB likely due to a low number of non-trophoblastic cells recovered during the initial placental dissociation. While *VIM* mRNA was expressed in TSCs, protein expression analysis (see below) provides evidence for minimal protein expression in TSCs relative to fetal fibroblasts. Low *CDX2* expression was observed in three of the four TSC and pri-CTB cultures evaluated. The absence of expression in rh090419 cells might be attributable to the gestational age (GD75) of the pri-CTB isolate. *ELF5* gene expression was observed in all pri-CTB and TSC cultures, with the exception of undetectable levels in TSCs of cy091318. mRNA expression of the macaque homolog of human *HLA-G*, the placental MHC class I molecule *Mamu-AG*^29^, was detected in all cells. The genes *CDH1* and *ITGA6* are highly expressed in human TSCs^21^ and mononuclear villous cytotrophoblasts^39–41^, and in the present study, were also expressed in macaque pri-CTB and TSCs.

To survey gene expression on a broader scale, RNA-seq was performed on a single set of rh121118 cells including primary trophoblasts, reprogrammed TSCs and differentiated TSCs. Deep-sequencing for the rh121118 line revealed distinct gene expression profiles for each trophoblast type (Fig. 3B, Supplementary Table S2). The rh121118 TSCs more highly expressed the genes *TP63*, *ITGA6*, *TEAD4*, *GATA3*, *GATA6* and *CDH1*, whereas pri-CTB more highly expressed *ID2*, *CSH1* and *4*, *ELF5*, *GATA4, GCM1* and syncytium induction genes, *ERVV1* and *ERVFRD1*. The pri-ST cells that were differentiated in vitro displayed expression of classic ST markers including *PLAC1*, *PLAC8*, *MMP9* and *Mamu-AG*. By comparison, the ST-3D cells were enriched in genes representative of an early post-implantation placenta, as *CGA* and *CGB* expression was highly represented in comparison to pri-ST. ST-2D cells had expression profiles similar to both TSCs and ST-3Ds, with markedly high expression of *TFAP2A*, *GATA2*, *HAND1* and *ESRRB*, suggesting that these transcription factors may underlie initial commitment to ST differentiation.

The evaluation of known human EVT markers^6,21,41^ revealed distinct gene expression profiles between macaque TSCs and TSC-derived EVTs. The RNA-seq analysis showed upregulation of EVT markers *Mamu-AG*, *NOTCH2*, *MMP2* and *ITGA5* in TSC-derived EVTs compared to TSCs (Fig. 3C, Supplementary Table S3). EVT marker expression was also validated in five cell lines of TSCs and their derived EVTs by reverse transcription quantitative PCR (RT-qPCR). The EVT markers *ITGA1*, *ITGA5*, *MMP2* and *NOTCH2* were significantly differentially expressed with higher expression in EVTs compared to TSCs; the mean fold changes in expression were 20.58 (p = 0.005), 27.63 (p = 0.010), 14.27 (p = 0.03) and 23.15 (p = 0.007), respectively (Fig. 3D). In comparison, *TCF4* was significantly elevated in TSCs with a mean fold change in expression of 28.85 (p = 0.002) compared to EVTs. There were no differences in expression between EVTs and TSCs for *CD9* and *FN1*, genes shown to be more highly expressed in EVTs as described by Okae et al.^21^, or *NOTCH1,* a marker highly expressed in human EVT progenitor cells^42^.

### Placental miRNA cluster expression

Primate trophoblasts express pregnancy-associated miRNA clusters, including the miR-371-3 cluster, C19MC, and chromosome 14 microRNA cluster (C14MC)^43,44^. miR-seq analysis was performed on cells derived from the rh121118 line to comprehensively survey miRNA expression. The miR-371-3 cluster is conserved in mammals and predominantly expressed in the placenta and embryonic stem cells with roles in cell cycle regulation^43,45–49^. miR-seq analysis of the miR-371-3 cluster in rh121118 trophoblast cell types revealed elevated expression of this cluster’s miRNAs in TSCs compared to pri-CTB, differentiated trophoblasts and rhesus fetal fibroblasts (Fig. 3E, Supplementary Table S4). miR-371-5p expression was ~ 800-times higher in TSCs compared to pri-CTB, with expression also detected in ST-2D and ST-3D cells. When assessing C19MC expression, the pri-CTB clustered with TSC and ST-2D expression levels while the pri-ST and ST-3D clustered together (Fig. 3F). Thus, ST cells, regardless of origin, highly expressed members of the C19MC (Fig. 3F). Rhesus fetal fibroblasts were also sequenced in parallel, and as expected, placenta-specific C19MC miRNAs were detected at very low to undetectable levels (0 to < 5 TPM; Supplementary Table S4). C14MC miRNAs were not detected in TSC or differentiated trophoblasts, whereas primary trophoblasts and fibroblasts weakly expressed C14MC miRNAs (Supplementary Table S4).

### TSCs display methylation signatures characteristic of trophoblasts

**Figure 4:**
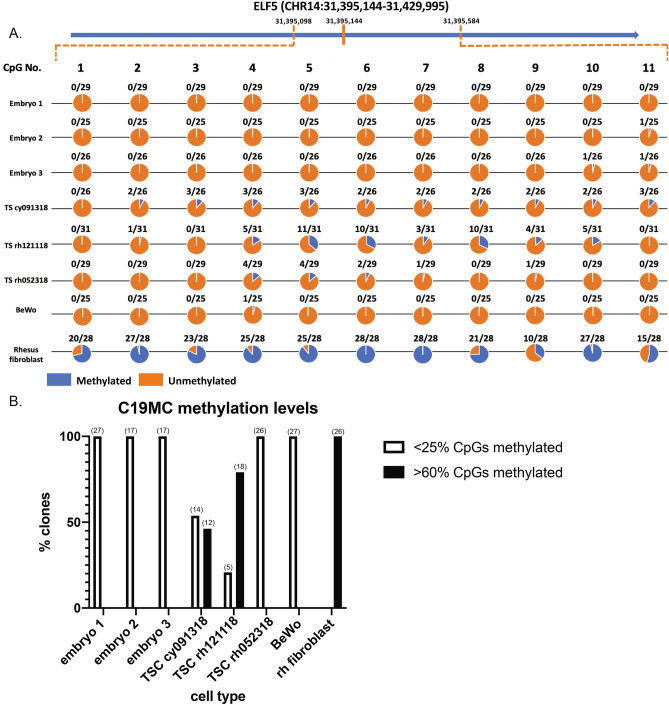
DNA methylation of ELF5 and C19MC gene regions. Methylation was evaluated in macaque blastocyst stage embryos (embryos 1–3), TSC lines, BeWo trophoblasts, and rhesus macaque fetal fibroblasts. (**A**) ELF5 methylation at 11 CpG sites for each cell type. Circles are colored to indicate the proportion of clones either methylated (blue) or unmethylated (orange) at each CpG site. (**B**) C19MC methylation at 34 CpG sites represented as the number of clones with either < 25% or > 60% methylation. The number of clones analyzed is indicated in brackets above each bar. All clones analyzed were either < 25% or > 60% methylated, representing an either hypo- or hyper-methylated allele, respectively.

DNA hypomethylation near the *ELF5* transcription start site is a feature of human trophoblasts^50,51^, and the lack of methylation coincides with ELF5 expression^50^. Methylation profiles were established for three TSC lines, three macaque blastocyst stage embryos, human BeWo choriocarcinoma cells and a rhesus fetal fibroblast cell line within a 440 base pair amplicon containing an island of 11 CpG sites surrounding the macaque *ELF5* start site (Fig. 4A). Relative hypomethylation was observed in all three TSC lines, embryos and BeWo cells, while fibroblasts displayed a hypermethylated profile as expected. The lack of ELF5 methylation near the transcription start site with ELF5 mRNA expression in macaque TSCs further supports that these cells maintain features previously described for human primary trophoblasts^50^.

Imprinted genes are vital to embryonic and placental development, and are monoallelically expressed from one parental allele while the other parental allele is methylated and not expressed^52^. The C19MC is positioned within an imprinted gene region, in which an imprinted methylation signature at the C19MC DMR1 has been described in human term placentas^53^. Here, methylation of the C19MC DMR1 was assessed similar to ELF5 to evaluate the imprinting status of macaque TSCs. An imprinted gene contains one hypermethylated allele and one hypomethylated allele based on the parent of origin, hence it would be expected that 50% of PCR clones would display either pattern in an imprinted state. TSC lines varied in the degree of methylation: rh052318 cells were nearly all hypomethylated, while the cells of cy091318 and rh121118 displayed both hypo- or hyper-methylated alleles (Fig. 4B). In comparison, hypomethylation was observed in macaque blastocyst stage embryos and BeWo cells, whereas rhesus fibroblasts which do not express C19MC miRNAs were predominantly hypermethylated.

### Trophoblasts and differentiated ST protein expression

**Figure 5:**
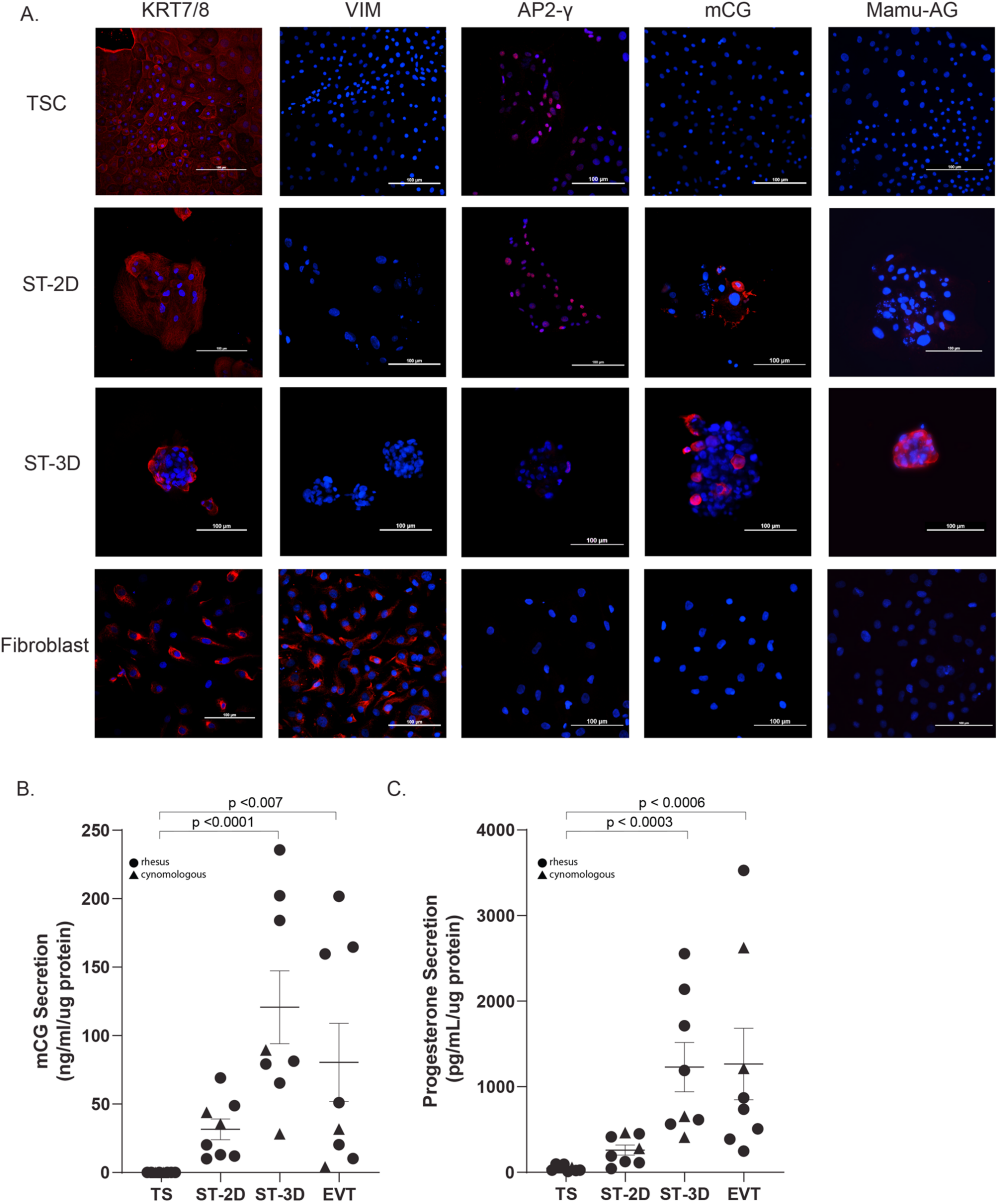
Differentiated trophoblast display protein markers of syncytium formation. (**A**) Representative observations for immunocytochemistry against KRT7, Vimentin, AP2-γ, mCG and Mamu-AG is represented in TSC, ST-2D, ST-3D and rhesus fetal fibroblasts. For all panels, the specific marker of interest is shown in red and the nuclei are stained with DAPI in blue. Scale bar denotes 100 µM. Secretion of mCG (**B**) and progesterone (**C**) by TS, ST2D, ST3D, and EVT cells was normalized to the amount of protein. Data is represented as the mean ± the standard error of the mean (SEM). Data for rhesus macaque cells is indicated by circles, whereas data for cynomolgus macaque cells is indicated by a triangle. Significance was determined using a Kruskal–Wallis test with Dunn’s correction.

Trophoblast protein marker expression was evaluated by immunocytochemistry. Trophoblasts highly expressed KRT7/8, while vimentin was not detected (Fig. 5A, see Supplementary Fig. S2 for IgG control images). Rhesus fetal fibroblasts also unexpectedly stained positive for KRT 7/8, however, differing cell culture conditions have been shown to support cytokeratin 8 expression in human fetal fibroblasts^54^. Interestingly, a classic trophoblast transcription factor, AP2-γ, encoded by the TFAP2C gene, was present in both TSC and ST cell types. More intense staining of macaque chorionic gonadotropin (mCG) and Mamu-AG, macaque ST differentiation markers, was observed in ST-3D aggregates compared to ST-2D cells (Fig. 5A). Differentiated trophoblast marker expression further supports the induction of TSC differentiation to ST.

### TSC-derived trophoblasts secrete pregnancy hormones

The secretion of CG and progesterone by placental cells, including ST and EVT cells^55^, is essential for the establishment and maintenance of primate pregnancy^56^. Secretion of mCG was significantly upregulated in ST-3D (120 ng/mL/µg protein) and EVT (80 ng/mL/µg protein) media, at ~ 4000-fold higher levels in comparison to TSC-conditioned media (0.02 ng/mL/µg protein) (Fig. 5B, p < 0.007 and p < 0.0001, respectively). ST-2D mCG secretion (31 ng/mL/µg protein) was ~ 1500-fold higher than TSCs, approaching statistical significance (p = 0.07). The levels of mCG detected in TSC-conditioned media were near the lower limit of detection. Of note, the quantity of mCG secreted by EVTs varied quite substantially across cell lines. Progesterone secretion was also ~ 1000-fold higher in ST-3D (1230 pg/mL/µg protein) and EVT (1265 pg/mL/µg protein) cultures compared to TSCs (48 pg/mL/µg protein; p < 0.0003 and p < 0.0006, respectively) and ~ 5-fold higher in ST-2D (260 pg/mL/µg protein) media compared to TSC media (Fig. 5C). As with mCG secretion, the levels of progesterone secretion by EVTs were similar to that of differentiated ST-3D cells. Compared to human pregnancy where CG levels peak in the 10th week of gestation^56,57^, CG secretion in macaques is restricted to an earlier stage of pregnancy with detection between 8–12 days post-implantation^58,59^. Thus, the ST differentiated from TSCs reestablish mCG secretion, a feature of early macaque pregnancy.

### Differentiation of TSCs to EVTs

**Figure 6:**
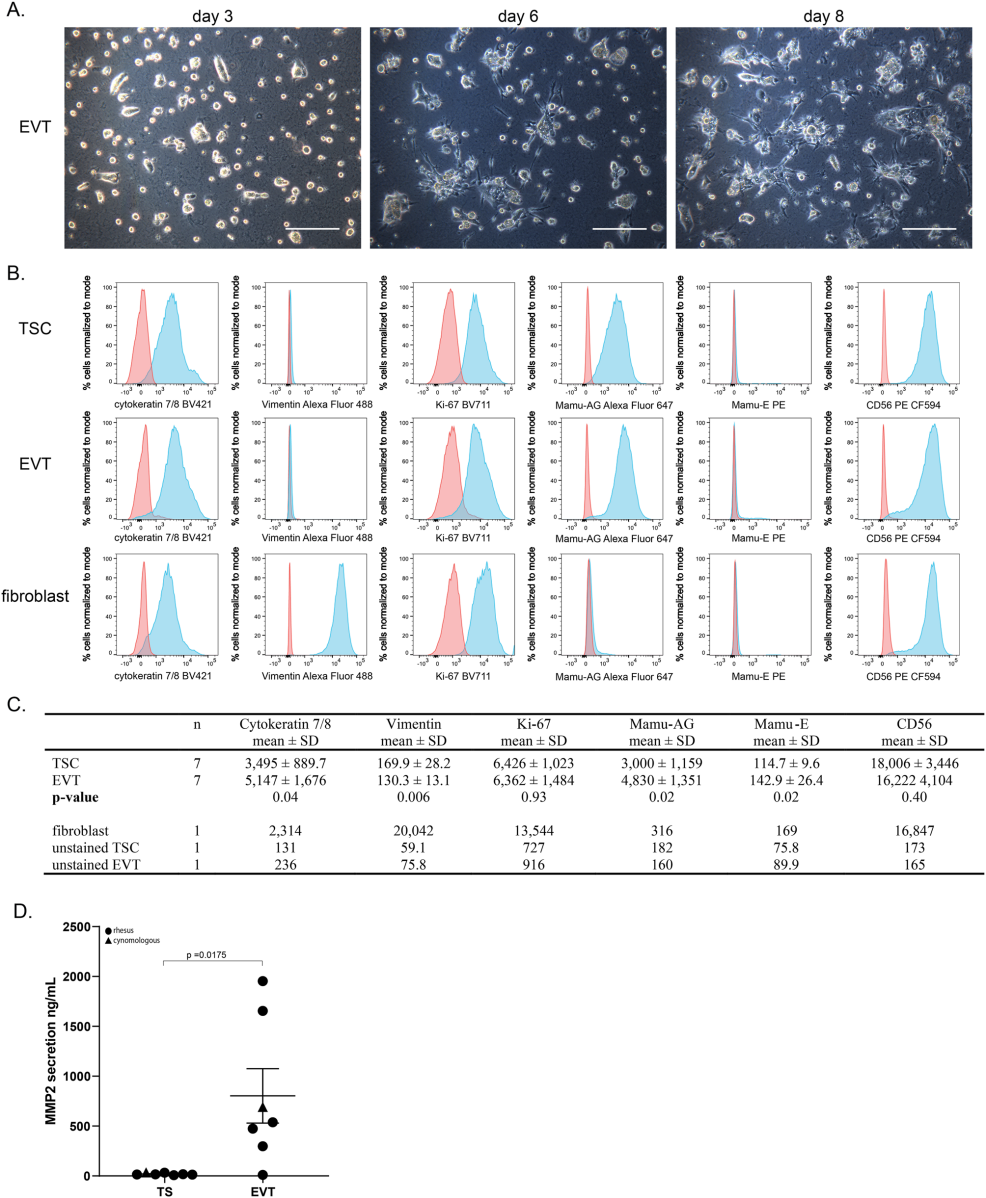
TSCs differentiate to an EVT-like cell type. (**A**) Morphology representative of EVTs derived from rh121119 TSCs on day 3, 6 and 8 of culture. Scale bar denotes 250 µM. (**B**) Representative flow cytometry histograms for TSCs, EVTs and rhesus fetal fibroblasts against the following proteins: cytokeratin 7/8, vimentin, Ki-67, Mamu-AG, Mamu-E and CD56. The proportion of cells that are stained (blue) or unstained (red) are represented as the % cells normalized to mode and the x-axis is the fluorescent intensity of the fluorophore. (**C**) Summary of the flow cytometric analysis represented as the mean and standard deviation (SD) of the median of fluorescence intensity across 7 TSC and EVT lines. A t-test was performed to assess differences in the median of fluorescence intensity between TSCs and EVTs. Control data including stained rhesus fetal fibroblasts and unstained TSCs and EVTs are also shown for comparison. (**D**) Secretion of MMP2 by EVTs compared to TSCs.

The ability of macaque TSCs to differentiate to EVTs was assessed by applying EVT culture conditions as described by Okae et al.^21^. Culture of macaque TSCs in EVT medium resulted in small round colonies of cells with limited clonal expansion or outgrowth throughout the duration of culture (Fig. 6A). EVT morphology varied slightly across lines, but was more consistent within an individual line (Supplementary Fig. S3).

Whereas the TSC and ST cultures were supported on col IV-coated glass coverslips for immunocytochemistry evaluation, EVT cell growth and adherence was not well-supported. Previous studies have demonstrated positive staining for Mamu-AG^28,29^, Mamu-E^28^ and CD56 (NCAM1)^60^ in macaque EVTs within the trophoblastic shell and in those that have invaded maternal vessels, thus these markers were evaluated by flow cytometry to assess TSC-derived EVT marker expression. Flow cytometry analysis was performed for seven cell lines of TSCs and EVTs, and rhesus fetal fibroblasts were assessed to serve as a negative control for placenta-specific markers (Fig. 6B,C; Supplementary Fig. S5). The mean percentage of TSCs and EVTs that displayed positive staining for cytokeratin 7/8, vimentin, Ki-67, Mamu-AG, and CD56 was similar between TSCs and EVTs, however, the proportion of Mamu-E positive cells was doubled in EVTs compared to TSCs (14.9% ± 4.5 SD vs. 7.0% ± 2.1 SD, respectively; Supplementary Table S5). Although the proportion of positively stained cells for each marker was similar between EVTs and TSCs, the differences in the mean of the median fluorescence intensity revealed protein expression differences between EVTs and TSCs. The mean of the median fluorescence intensities of Mamu-AG, Mamu-E, and cytokeratin 7/8 were significantly increased in EVTs compared to TSCs, whereas there were no differences in the median fluorescence intensities of Ki-67 and CD56 (Fig. 6C). Additionally, the mean of the median fluorescence intensity of vimentin was significantly increased in TSCs relative to EVTs (Fig. 6C).

Human EVTs express proteases, such as matrix metalloproteinases (MMPs), which act to degrade the extracellular matrix of the decidua allowing for cellular invasion^61–63^. More specifically, MMP2 is highly expressed in early first trimester human EVTs^64^, where it is thought to have a role in trophoblast invasion^62,64^. In the present study, the secretion of MMP2 in EVT and TSC media was assayed by a MMP2 ELISA. The secretion of MMP2 was significantly increased by ~ 40-fold in EVT-conditioned media with a mean of 803.0 ng/mL ± 721.3 SD compared to 19.62 ng/mL ± 10.41 SD in TSC-conditioned media (Fig. 6D). The elevated secretion of an invasion-promoting MMP by EVTs suggests that TSCs are capable of differentiation to an EVT-like cell.

### Single Cell RNA-sequencing of early and established TSC passages

Single-cell RNA-sequencing (scRNA-seq) was performed to assess the heterogeneity of TSC cultures. An early (p2) and a later (p10) TSC passage were assessed to determine gene signature changes with passage, and to define the transcriptome of the p10 cells that were used for initiating TSC differentiation. TSCs of rh121118 were sequenced to allow for interpretation of results relative to the full survey of the transcriptome obtained by RNA-seq as described above. A total of 11,311 cells (p2: 6052 cells, p10: 5259 cells) were sequenced, in which 73,091 mean reads were detected per cell and the median number of genes per cell was 1468. The gene expression of p2 cells clustered more tightly with no differentially expressed genes between clusters, indicative of a more homogeneous population than that of p10 cells (Supplementary Fig. S4). Comparatively, p10 cell gene expression segregated into 11 clusters with differentially expressed genes between seven clusters (Supplementary Fig. S4, Supplementary Table S6).

**Figure 7:**
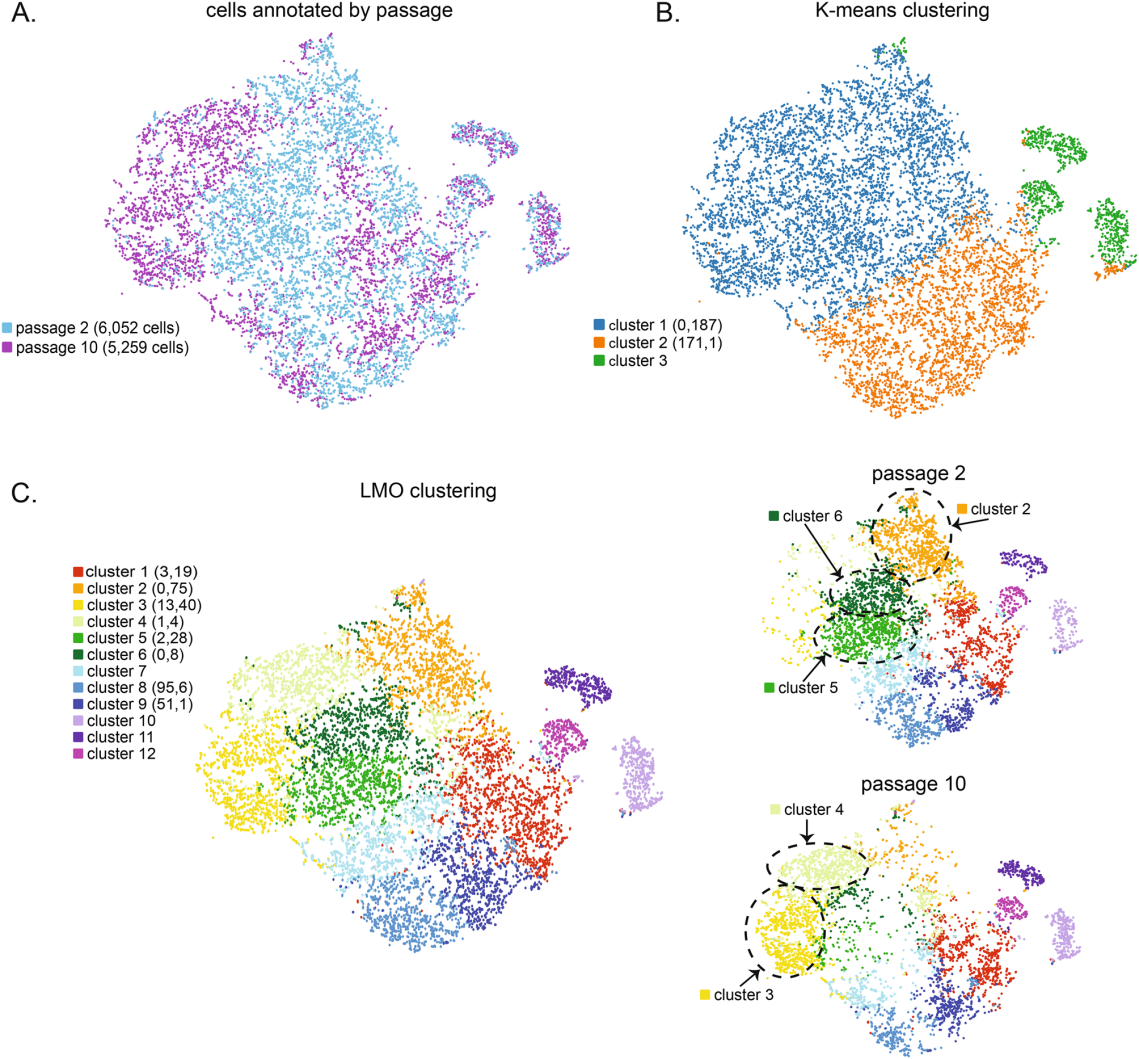
TSC gene expression evaluated by scRNA-Seq. (**A**) Identification of individual p2 and p10 TSCs of rh121118 within the t-SNE distribution with the number of individual cells sequenced for each passaged denoted in parentheses. (**B**) Gene expression by K-means clustering analysis of combined p2 and p10 cells. The number of significantly up or down regulated genes in this and panel C appear in parentheses next to the cluster number. (**C**) Clustering of p2 and p10 cells upon analysis of gene expression levels using the LMO algorithm. Individual p2 and p10 cells from the combined analysis are shown to the right. Clusters where cells were more highly represented from one of these two passages are indicated by gray circles.

To assess differences in expression profiles between passages, a K-means clustering analysis and a Louvain Modularity Optimization (LMO) algorithm^65^ were used to cluster cells on the number of nearest neighbors (based on cellular gene expression levels) and plotted using a dimensionality reduction method, t-Stochastic Neighbor Embedding (t-SNE). The t-SNE plot revealed that cells of both passages clustered together as well as segregated by passage (Fig. 7A). The K-means analysis of combined TSCs of p2 and p10 revealed three distinct cell clusters (Fig. 7B) with an inverse differential expression profile between clusters 1 and 2 (Supplementary Table S7). Genes that were up-regulated in cluster 2 were down-regulated in cluster 1 cells, including highly differentially expressed genes *NUCB2*, *UBB*, *TECR*, *PMEL*, *UBC*, *APOE*, *MRPS31*, *PRDX2*, and *LDHA*; see Supplemental Figure S5 for individual cell marker expression. This would suggest that two distinct populations are present regardless of passage.

Application of the LMO algorithm further segregated cells into twelve clusters, where the cells that comprised clusters 3 and 4 were predominantly cells of p10 and cells of clusters 2, 5, and 6 were largely of p2 (Fig. 7C). Differentially expressed genes were shared among clusters (Supplementary Table S8), such as the downregulation of *TOP2A*, *CDK1*, *GTSE1* and *UBE2C* in Clusters 1, 2 and 4 with upregulation of *TOP2A* and *GTSE1* in Cluster 5; see Supplementary Fig. S5 for individual cell marker expression. Gene ontology (GO) of biological processes for downregulated genes of cluster 2, 4, and 6 featured mitotic cell cycle transition as a common term between them, whereas alteration of the Wnt signaling pathway was associated with the downregulated genes of clusters 2, 3, 4 and 5. The similarities in biological processes by multiple clusters is likely a reflection of genes similarly differentially expressed across clusters, and these clusters are more likely segregating by subtle changes in expression patterns of many genes rather than by large differences in individual genes.

**Figure 8:**
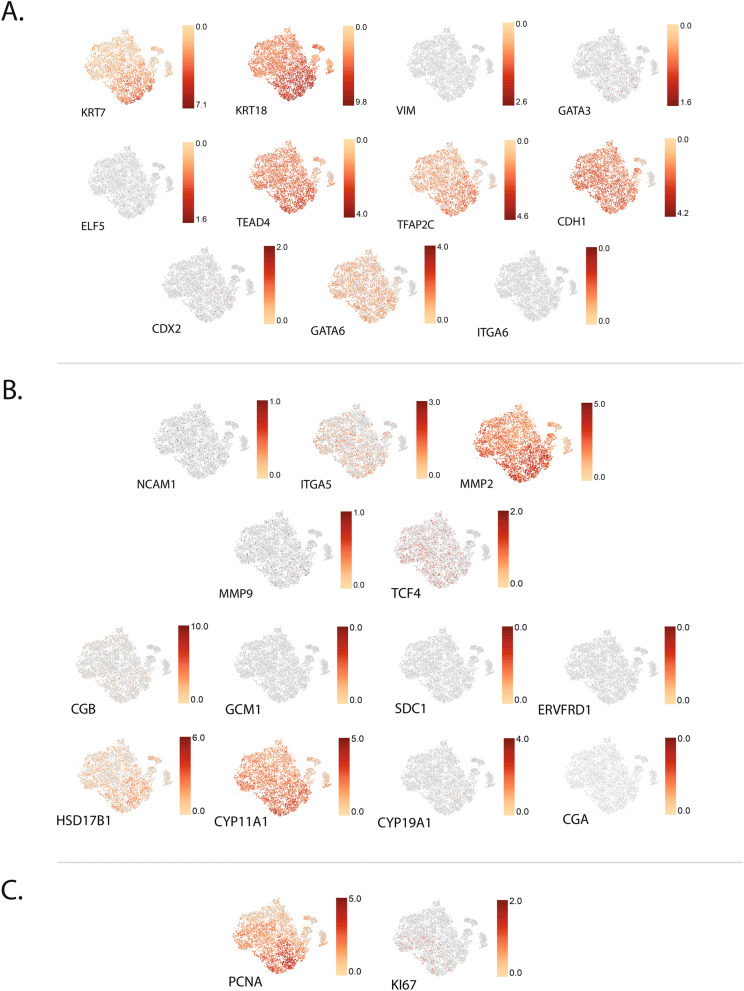
scRNA-seq expression of individual genes associated with trophoblasts and cellular proliferation in TSCs. (**A**) Representation of known genes expressed in human villous cytotrophoblasts or TSCs, (**B**) ST or EVT differentiation and (**C**) cell proliferation. Scale bar represents the range in Log_2_ expression for the marker for all panels.

When evaluating expression of classic trophoblast markers in individual cells, the expression of these markers was not significantly different between clusters and rather their expression was represented throughout the t-SNE distribution (Fig. 8). TSCs highly express known human TSC markers such as *KRT7*, *CDH1*, *TEAD4*, *TFAP2C*, and *GATA6* (Fig. 8A) and lowly express ST or EVT differentiation markers (Fig. 8B). A limited number of transcript variants were represented in the analysis software, and the single variant depicted here may be a more lowly expressed variant (Fig. 8A). In addition, no annotation was provided for macaque *CDX2* or *Mamu-AG*. The TSCs widely expressed cell proliferation markers *PCNA* and *Ki67* (Fig. 8C).

## Discussion

The present study demonstrates the derivation of macaque TSCs that not only express key trophoblast features, but also are capable of continued proliferation and can be differentiated to STs and putative EVTs. Using the paradigm established for the derivation of human TSCs, we have shown that macaque TSCs can be derived from early placentas that show similar features to human trophoblasts^51^, including expression of trophoblast transcription factors and C19MC miRNAs as well as ELF5 promoter DNA hypomethylation. Macaque TSC-derived STs and EVTs displayed differentiation markers characteristic of macaque trophoblasts, such as Mamu-AG expression and elevated secretion of pregnancy hormones. Altogether, the macaque trophoblast cell-type specific marker expression shows that the human TSC culture conditions can be applied to reproducibly derive macaque TSCs and differentiated trophoblasts.

Under standard culture conditions, macaque pri-CTB do not proliferate but will spontaneously differentiate within 48–72 h to multinuclear syncytia^35,66^, similar to human trophoblasts^17^. A molecular switch occurs under TSC culture conditions to support cell self-renewal rather than terminal differentiation as the pri-CTB quickly assume a mononuclear, proliferative state within a few days of culture. Single-cell RNA-seq of an early and later passage of TSCs revealed similarities in differentially expressed genes across clusters rather than exclusive expression within a cluster. The differentially expressed genes were predominantly those that regulate the cell-cycle, and thus likely reflect cell populations that are at different stages of the cell cycle. The present study did not synchronize the cell cycle, but this should be considered for future scRNA-seq experiments. Interestingly, the K-means analysis revealed a distinct axis of up or down- regulated genes, including *NUCB2*, *APOE*, *PRDX2*, and *LDHA*, that have previously been shown to have roles in trophoblast function^67–71^. The roles of these genes in TSC function and maintenance, however, remains unclear. Genes known to be expressed in human undifferentiated trophoblasts, regardless of clustering method, were expressed across macaque TSCs with minimal expression of differentiation markers, indicating a more homogenous TSC population.

Macaque TSCs display trophoblast marker expression and DNA hypomethylation features similar to human TSCs. Based on flow cytometry and immunocytochemistry findings, all TSC lines were predominantly cytokeratin-positive and vimentin-negative. Macaque TSCs expressed the genes *TEAD4*, *TP63* and *TFAP2C* as similarly reported for human TSCs^21,22^. The expression of *CDX*2 was observed in pri-CTB and TSCs by RT-PCR but not in the pri-CTB or TSCs derived from a later gestational day 75 placenta. *CDX2* expression in human placentas declines as gestation progresses^50^, but the expression in the macaque placenta throughout gestation and the role of *CDX2* in TSCs remains elusive. Similarly, *ELF5* displayed lower mRNA expression in TSCs compared to pri-CTB by RNA-seq. It is important to note that at the genomic level, a hypomethylated pattern was observed near the *ELF5* gene start site, similar to observations in human TSCs^21–23^ and placentas^50^. Due to the lack of antibodies validated for macaque ELF5 or CDX2, the expression of these proteins was not confirmed here. There is limited characterization of gene or protein expression of trophoblast transcription factors in the macaque, hence future studies with histological and quantitative gene expression analysis of human trophoblasts markers in macaque placentas and TSCs are needed to confirm conservation of trophoblast transcription factor expression.

An important hallmark of human and nonhuman primate trophoblasts is the expression of placental miRNA clusters^30,43,44^. This is the first study to evaluate C19MC methylation in macaque cells and embryos, and unexpected methylation patterns were observed. As the C19MC is a maternally imprinted gene region, it was expected that the maternal allele would be hypermethylated while the paternal allele would be hypomethylated^53^. Although a 1:1 ratio of hyper- to hypo- methylated DNA clones from TSCs was anticipated, the methylation at the C19MC DMR1 locus varied across TSC lines ranging from a more hypomethylated to a near 1:1 ratio of hypo- to hyper-methylated DNA clones. Moreover, in vitro fertilized whole embryos that contained both inner cell mass and trophectoderm cells were analyzed in parallel. The unexpected imprinting pattern of the C19MC locus in both embryos and trophoblasts may be explained by differences in imprinting between humans and macaques, or the failure of the in vitro derived embryos evaluated here to maintain imprinting at this region. Alterations in genomic imprinting have been associated with in vitro embryo culture^72,73^. The survey of methylation at the C19MC DMR1 in expanded macaque blastocysts is a preliminary finding with only three embryos evaluated in this study, and it is difficult to interpret our findings as there are no reports of the imprinting status of C19MC DMR1 in human or nonhuman primate whole embryos. The variation in C19MC methylation across TSC lines also warrants future investigation as to whether the degree of methylation correlates directly with the cluster’s expression.

The miR-371 cluster is also predominantly expressed in the mammalian placenta^47^ and is highly expressed in ESCs^49,74^ with decreased expression upon ESC differentiation^49^. The expression of the miR-371 cluster tends to be higher in the first trimester macaque placenta^30^. In the present study, macaque TSCs exhibited an upregulation of the miR-371 cluster miRNAs compared to primary cytotrophoblasts and differentiated trophoblasts. Knock-out of the orthologous mouse miRNA cluster, the miR-290 cluster, suggested that the miRNAs of this cluster have a role in the cell cycle and in maintenance of mitosis in trophoblast progenitor cells^75^. Zhou et al.^76^ demonstrated a dynamic relationship between the miR-371 cluster and the Wnt/ß-catenin signaling pathway, whereupon stimulation of Wnt/ß-catenin signaling the expression of the miR-371 increased. Moreover, the upregulation of miR-371 cluster miRNAs induced the expression of cell cycle regulators *c-Myc*, *c-Jun*, and *cyclin-D1*^76^. The human and macaque TSC media were supplemented with the GSK-3 inhibitor, CHIR99021, to activate Wnt signaling. Here, we show that activation of the Wnt signaling pathway promotes placental miR-371 cluster expression, in which the interaction between Wnt signaling and the miR-371 cluster plausibly acts to regulate the stemness and continued proliferation of primate TSCs.

Upon culture of TSCs with forskolin, ST differentiation occurs with elevated secretion of mCG, syncytium formation and expression of ST markers. ST-3D aggregates demonstrated higher RNA expression for CGA and CGB and trended towards greater secretion of mCG compared to ST-2D cultures. ST-2D cultures retained a subset of mononuclear TSCs and expressed a gene profile that may reflect initial or partial commitment to ST differentiation. Importantly, the observance of mCG secretion is significant as it indicates a “reprogramming” to an early pregnancy trophoblast phenotype, and thus, macaque TSCs may be suitable for modeling events of early placental development.

Macaque TSCs were capable of differentiating to cells that display an expression profile similar to early gestation in vivo macaque EVTs. Human EVTs exclusively express HLA-G^77^, allowing for a definitive characterization of a differentiated EVT. Comparatively, there are currently no known markers to uniquely characterize macaque EVTs, although markers such as Mamu-AG and the cell adhesion molecule, CD56 (NCAM1), are highly expressed in differentiated macaque trophoblasts of the trophoblastic shell and endovascular EVTs^28,29,60^. Unlike human HLA-G, the MHC class I ortholog, Mamu-AG, is not exclusive to macaque EVTs but rather the protein is expressed in both ST and EVT cell populations^28^. Slukvin et al.^27,29^ previously showed weak in situ hybridization signal for Mamu-AG in villous cytotrophoblasts, thus the expression of Mamu-AG in TSCs is not surprising. Interestingly, Mamu-AG protein expression has also been demonstrated in rhesus blastocyst-derived trophoblast outgrowths^26,32^, similar to the expression of HLA-G in human blastocysts^78^. In the present study, the presence of Mamu-AG protein in TSCs was detectable by flow cytometry, however, the fluorescence intensity of the staining was significantly upregulated in TSC-derived EVT cells. Elevated staining intensity of Mamu-E was also observed in TSC-derived EVT cells. Dambaeva et al.^28^ previously demonstrated that endovascular EVTs of the macaque first trimester placenta (gestational day 36) display highly positive staining for Mamu-E, whereas the cells of the trophoblastic shell are weakly positive. Moreover, co-expression of Mamu-E and Mamu-AG were noted in endovascular EVTs. Hence, the small proportion of Mamu-E positive TSC-derived EVTs may be indicative of a more differentiated EVT population. Macaque trophoblasts of the distal cell columns, trophoblastic shell and endovascular EVTs express CD56^60^, and in the present study the staining of CD56 was elevated in TSC-derived EVTs. Regardless of cell line, EVTs highly expressed Mamu-AG and CD56 and the significant elevation in staining intensity of these markers supports the directed differentiation of TSCs to a macaque EVT-like cell.

To provide further evidence that macaque EVTs display a differentiated expression profile, in vitro derived macaque EVTs were also assessed for known human EVT markers. Macaque EVTs demonstrated upregulation of the genes *ITGA1*, *ITGA5*, and *MMP2*, markers that are highly expressed and characteristic of human EVTs^6,21,40,41^. It is unclear whether the macaque EVTs are reprogrammed to an earlier gestational age, such as was observed with TSC-derived ST. TSCs and EVT-derived cells expressed similar levels of *NOTCH1*, a key regulator of EVT differentiation expressed in human progenitor cells that give rise to EVTs^79^. In comparison, the higher expression of *NOTCH2*, a marker of fully differentiated human EVTs, was observed in macaque TSC-derived EVTs compared to TSCs. The increased expression of *NOTCH2* and integrins associated with an invasive trophoblast cell type, in conjunction with high secretion of MMP2 and elevated protein expression of macaque Mamu-AG, provides evidence that macaque TSCs are capable of directed differentiation to EVTs.

Notably, Ki-67, a proliferation marker, was also observed in the TSC-derived EVTs, although, this finding was not unexpected. Blankenship and King^38^ described positive Ki-67 staining in some cells of the macaque trophoblastic shell at gestational days 25 and 45, with diminished presence of Ki-67 by gestational day 100. In the study by Okae et al.^21^, human TSC-derived EVTs were passaged on day 6 of differentiation, whereas the macaque EVTs comparatively had reduced confluency despite higher plating densities on day 0 of differentiation initiation. Identification of macaque trophoblastic shell and endovascular EVT markers are needed to confirm optimal conditions for the derivation of macaque EVTs in vitro. Until such macaque markers are identified, studies using macaque in vivo specimens are needed to assess the conservation of human EVT markers in nonhuman primate placentas.

In conclusion, macaque pri-CTB can be driven to TSCs utilizing derivation conditions previously described by Okae et al.^21^ for human embryonic trophectoderm and placental pri-CTB. The present study describes the generation of both rhesus and cynomolgus macaque TSCs from placental villous cytotrophoblasts, which is a significant advance since placentas are a more widely available macaque resource for derivation compared to blastocysts. The capacity of macaque TSCs to self-renew and differentiate to ST and EVT under a tightly-controlled culture system offers a platform for modeling early primate trophoblast differentiation and development in vitro. A unique finding in the present study was the reestablishment of CG expression in the TSC-derived ST, which presents an opportunity to model the earliest stages of placental development. Experimental perturbations can first be evaluated within the in vitro TSC model with subsequent translation to macaque embryos and in vivo pregnancy, allowing for comprehensive study at the cellular and organismal level. For instance, the macaque TSC model can be directly used to perturb gene functions underlying trophoblast differentiation processes, evaluate trophoblast response to experimental infection, or assess the efficacy of placental therapeutics tailored to specific trophoblast cell types. Implementing a macaque TSC model with subsequent translation to in vivo pregnancy studies could be pivotal to understanding mechanisms of early placental development and allow for the development and/or evaluation of treatments or therapeutics to improve human pregnancy health.

## Methods

### Subjects

Rhesus and cynomolgus macaques were obtained from the colony maintained at the Wisconsin National Primate Research Center (WNPRC). This study was performed in strict accordance with the recommendations by the National Research Council Guide for the Care and Use of Laboratory Animals in an AAALAC accredited facility (WNPRC). Experimental procedures were approved by the University of Wisconsin-Madison Institutional Animal Care and Use Committee (protocols: g005061, g005691, g005592). Macaque placentas were collected between gestation day 40–75.

### Placenta cell dissociation

First trimester placental tissue was dissociated to obtain primary villous cytotrophoblasts (pri-CTB) utilizing a trypsin/DNase and Percoll gradient isolation method previously described without any modifications^66^. Briefly, the placenta was surgically collected at uterotomy and the decidua was removed from the placental discs. The fresh placental tissue was rinsed in sterile phosphate-buffered saline, minced with sterile scissors, and subjected to three rounds of cell dispersion by shaking for 30 min in a trypsin/DNAse solution. Cells liberated from each round of enzymatic dispersion were pooled, resuspended in DMEM without serum, and layered onto a discontinuous 5–50% Percoll gradient (Percoll, Sigma-Aldrich, cat no: P4937) followed by centrifugation at 2200 × *g* for 25 min. A representative image of the Percoll gradient and cell layers following the centrifugation step is shown in Golos et al.^66^. The trophoblast layer was isolated using a serological pipette and transferred to a new tube. The trophoblast suspension comprised of pri-CTB was washed in 4 volumes of DMEM, spun at 240 × *g* for 10 min, and the cell pellet was resuspended in DMEM. The pri-CTB were then counted using a hemocytometer. Pri-CTB were either cultured as TSC or were differentiated into pri-ST by plating ~ 1.5–2 × 10^6^ cells per 60 mm dish and grown in DMEM with 10% fetal bovine serum (FBS, Peak Serum, cat no: PS-FB1) for 72 h. The pri-ST and pri-CTB were collected into TRIzol Reagent (Invitrogen, ThermoFisher Scientific, cat no: 15596018) for RNA isolation.

### Generation of trophoblast stem cells

Macaque TSCs and differentiated trophoblast cells were derived utilizing similar methods as those described by Okae et al.^21^ to generate human TSCs and subsequently differentiated trophoblast cell populations. Of note, primary macaque trophoblasts were not purified based on ITGA6 marker selection as described by Okae et al.^21^, however, the isolation method described above for macaque villous cytotrophoblasts has previously shown to consistently isolate trophoblast cells with > 90% cytokeratin-positive staining^35,66^. Primary-CTBs were initially plated at a density of 2–5 × 10^5^ cells per well of a 6-well plate coated with 5 µg collagen IV (col IV, Corning, cat no: 354233) and maintained in trophoblast stem cell (TSC)-medium described by Okae et al.^21^ consisting of DMEM/F12 supplemented with 0.1 mM 2-mercaptoethanol (Gibco, ThermoFisher Scientfic, cat no: 31350010), 0.2% FBS (Peak Serum, cat no: PS-FB1), 0.5% Penicillin–Streptomycin, 0.3% BSA (Sigma-Aldrich, cat no: A19335G), 1% ITS-X supplement (ThermoFisher Scientific, cat no: 51500056), 1.5 µg/mL l-ascorbic acid (Wako Chemical USA, cat no: 013-12061), 50 ng/mL EGF (ThermoFisher Scientific PHG0313), 2 µM CHIR99021 (Tocris, cat no: 4423), 0.5 µM A83-01 (Tocris, cat no: 2939), 1 µM SB431542, 0.8 mM VPA (Sigma-Aldrich, cat no: P4535) and 5 µM Y27632 (Tocris, cat no: 1254). Cell cultures were maintained at 37 °C and 5% CO_2_ and medium was replaced every 1–2 days. Once cells reached 80–90% confluency, cells were passaged by incubating with TrypLE Select (Gibco, ThermoFisher Scientific, cat no: 12604021) at 37 °C for 15 min to lift the cells. For the first few passages, cells were plated at a density of 1–2.5 × 10^5^ cells per well of a 6-well plate coated with 5 µg col IV and then in subsequent passages 0.5–1 × 10^6^ cells per T75 flask coated with 25 µg col IV.

### In vitro trophoblast differentiation

TSCs were differentiated to ST utilizing either two-dimensional (ST-2D) or a three-dimensional (ST-3D) differentiation protocols as described by Okae et al.^21^, with modifications to the cell plating density and concentration of col IV used to coat the plates. To obtain ST-2D cells, TSCs were plated at a density of ~ 2 × 10^5^ cells per well of a 6-well plate coated with 5 µg collagen IV and maintained in ST-2D medium consisting of DMEM/F12 supplemented with 0.1 mM 2-mercaptoethanol, 0.5% Penicillin–Streptomycin, 0.3% BSA, 1% ITS-X supplement, 2.5 µM Y27632, 2 µM forskolin (Cayman Chemical, cat no: 66575-29-9) and 4% Knockout Serum Replacement (KOSR; ThermoFisher Scientific, cat no: 10828028). Medium was replaced on day 3 of differentiation. On day 6, cellular morphology was assessed and cells were collected. To obtain ST-3D cells, 2.5 × 10^5^ TSCs were seeded into a low binding T25 flask (Nunc, ThermoFisher Scientific, cat no: 169900). Suspended cells were maintained in 3 mL of ST-3D medium containing DMEM/F12 supplemented with 0.1 mM 2-mercaptoethanol, 0.5% Penicillin–Streptomycin, 0.3% BSA, 1% ITS-X supplement, 2.5 µM Y27632, 50 ng/mL EGF, 2 µM forskolin and 4% KOSR. An additional 3 mL of ST-3D medium was added on day 3 of differentiation. On day 6 of differentiation, the cell suspension was passed through a 40 µM filter to remove dead cells and debris. ST-3D aggregates were washed from the filter with DMEM/F12 medium and collected. For both ST-2D and ST-3D cultures, media were collected on day 6 of differentiation for hormone analysis.

To differentiate TSCs to EVTs, TSCs were plated at a density of ~ 1 × 10^5^ cells per well of a 6-well plate coated with 5 µg collagen IV per well. After adding the cell suspension to the well, Matrigel (Corning, cat no: 354234) was added at a final concentration of 2%. From day 0–3, cells were cultured in 2 mL of EVT medium comprised of DMEM/F12 supplemented with 0.1 mM 2-mercaptoethanol, 0.5% Penicillin–Streptomycin, 0.3% BSA, 1% ITS-X supplement, 100 ng/mL NRG1 (Cell Signaling, cat no: 5218SC), 2.5 µM Y27632, 7.5 µM A83-01 and 4% KOSR. On day 3 of culture, medium was exchanged with EVT medium lacking NRG1, and Matrigel was supplemented at a final concentration of 0.5%. On day 6 of culture, medium was replaced with 2 mL of EVT medium lacking NRG1 and KOSR. Matrigel was supplemented at a reduced final concentration of 0.5%. On day 8 of culture, cells were collected for analysis. EVT media were collected from cells on day 8 for hormone analysis.

### Rhesus fetal fibroblast cell culture

A rhesus fibroblast line was derived from a fetal skin biopsy of gestational age day 75. Fibroblasts were plated at a density of 1.0 × 10^6^ cells per T75 flask in DME/F12 medium (Fisher Scientific, cat no: sh20023fs) supplemented with 5% FBS (Peak Serum, cat no: PS-FB1), 1% sodium pyruvate (Lonza, cat no: BEBP13-115E) and 1% non-essential amino acids (Gibco, cat no: 11140-50). At 90% confluency, cells were passaged using 0.25% trypsin solution (2.5% Trypsin (Corning cat no: 25-053-Cl), 500 mM EDTA (Sigma-Aldrich, cat no: E5134), 100 mM sodium chloride (Sigma-Aldrich, cat no: S7653), and 20 mM TRIZma-base (Sigma-Aldrich cat no: T6066) or collected for nucleic acid extraction by freezing the cell pellet alone or in 1 mL TRIzol reagent (Invitrogen, cat no: 15596018, ThermoFisher Scientific) for subsequent DNA or RNA extraction, respectively.

### Cytogenetics

Karyotyping was performed by Cell Line Genetics (Madison, WI) using methods previously described^80^. In brief, cells were treated with 0.1 μg/mL colcemid for 40 min and then enzymatically treated to produce a single cell suspension. Cells were then incubated in 0.075 M KCl, followed by fixation using Carnoy’s fixative (3:1 methanol:glacial acetic acid). Fixed cells were dropped and dried onto slides using a Percival environmental chamber and baked at 90 °C for 60 min. Chromosomes were then banded by utilizing a trypsin treatment followed by staining with Geimsa. Images were taken of metaphase cells using a GSL scanner (Leica) and analyzed with the aid of CytoVision software (Leica).

### Immunocytochemistry

For TSC and ST-2D cultures, cells were cultured on col IV coated glass coverslips within a 6-well plate. TSCs were grown for 3–4 days prior to fixation, whereas ST-2D were fixed on day 6 of the differentiation paradigm. ST-3D aggregates were grown in suspension and on day 6 were plated on to col IV coated coverslips for 1–2 h to allow for attachment, immobilizing the aggregates for ease of handling and imaging. At the appropriate time points the cells were rinsed with PBS and fixed with 2% PFA for 10 min at room temperature. The coverslips with adhered cells were then washed twice with PBS and stored in PBS at 4 °C until processed. The samples were rinsed with PBS, permeabilized with 0.1% Triton-X 100 in PBS for 5 min, blocked using Background Punisher (Biocare Medical, cat no: BP974) for 10 min, rinsed and exposed to primary antibodies for 1 h at room temperature by floating on a 75 µL drop, see Supplementary Table S9 for antibody and fluorescent conjugate information. The coverslips were then washed with PBS three times for 5 min each, and floated on drops containing the appropriate conjugated secondary antibodies for 45 min at room temperature followed by three washes for 10 min each at room temperature with TBST (Tris buffered saline containing 0.1% Tween-20). The coverslips were then treated with DAPI (Invitrogen, cat no: D3571; 1:10,000 dilution) for 5 min at room temperature, rinsed with water, and mounted onto slides using Prolong Diamond Antifade Mountant (Invitrogen, cat no: 36970, ThermoFisher Scientific). Cells were imaged on either a Nikon A1R confocal or a Nikon Eclipse Epifluorescent microscope.

To visualize nuclei and cell membranes, cells were stained with wheat germ agglutinin (WGA) which binds to the glycoconjugates on the cell surface. Cells on coverslips were generated as described above, and fixed samples were rinsed with PBS but not permeabilized. The samples were then stained with WGA conjugated with Texas Red (5 µg/ml) and Hoescht 33342 for 10 min at room temperature, rinsed three times in PBS for 10 min each, followed by one rinse in water to removed salts prior to mounting with ProLong Diamond Antifade Mountant (Invitrogen, ThermoFisher Scientific, cat no: p36961) onto a glass slide. Cells were imaged on a Nikon A1R confocal microscope.

### RNA isolation

Cells collected for RNA isolation were resuspended with 1 mL of TRIzol reagent (Invitrogen, cat no: 15596018, ThermoFisher Scientific) and frozen until RNA extraction. The TRIzol-cell mixture was layered onto a Phasemaker Tube (Invitrogen, ThermoFisher Scientific, cat no: A33248) and incubated at room temperature for 3 min followed by addition of 200 µL chloroform (MP Biomedicals, ThermoFisher Scientific, catalog no: ICN19400280) and spun at 16,000*g* at 4 °C for 15 min to allow separation. The aqueous layer was recovered, 500 µL 70% ethanol was added, and then transferred a RNeasy Mini Kit (Qiagen, ThermoFisher Scientific, cat no: 74104) spin column and proper buffer washing steps were carried out following manufacturer recommendations including a 15 min on-column DNAse (Qiagen, ThermoFisher Scientific, cat no: 79254) treatment. RNA concentration was then quantified via Qubit Fluorometer using the RNA BR Assay Kit (Qiagen, ThermoFisher Scientific, cat no: Q10210) and quality was evaluated using an Agilent 2100 BioAnalyzer (Agilent, cat no: G2939BA).

### cDNA synthesis, RT-PCR and RT-qPCR

cDNA was synthesized using a SuperScript III First-Strand Synthesis System (Invitrogen, ThermoFisher Scientific, cat no: 18080-051). RNA from four cell preparations of each pri-CTB and corresponding TSC were combined with kit reagents and reactions were performed following manufacturer recommendations. PCR was performed by combining cDNA, GoTaq Hot Start Colorless Master Mix (Promega, cat no: M513B), RNAse-free water, and gene-specific primers (Supplementary Table S7). No cDNA control and no reverse transcriptase control reactions were run in parallel for each primer pair and cell type. The RT-PCR reactions were performed using a BioRAD C1000 Touch instrument. The PCR products were then run on a 2% agarose gel and the bands were analyzed to determine gene expression. The original gel images are shown in Supplemental Figure S6.

To quantify gene expression between TSCs and EVT, RT-qPCR analysis was performed for known human EVT markers^6,21,41^. RNA (500 ng) from TSCs and EVTs from 5 lines was reverse transcribed as described above. cDNA was combined with iQ SYBR green master mix (BioRad, cat no: 1708882), gene-specific primers (Supplementary Table S10) and RNase-free water. All primers were designed to span exon-exon junctions. Reactions were performed in triplicate with no cDNA negative controls and run on a Roche LC 96 instrument. An internal control, beta-actin (ACTB), was used a reference for comparison of expression levels. The fold change in expression was calculated using the 2^−ΔΔCt^ method^81^. A paired t-test was performed on the ΔCt values between TS and EVT for each cell line and gene using Prism 8 software (https://www.graphpad.com/scientific-software/prism/).

### Next-generation RNA sequencing

Total RNA was isolated from rh121118’s primary trophoblasts, TSCs and differentiated TSCs to survey which transcripts and miRNAs are present within each cell type (n = 1 replicate/cell type). For transcriptome analysis, ~ 1 µg total RNA was used as input for each cell type (pri-CTB, TSC, pri-ST, ST-2D, ST-3D, EVT and fibroblast) to generate cDNA libraries using a TruSeq Stranded mRNA library preparation kid (Illumina). Single-end 100 bp reads (1 × 100) were sequenced on an Illumina HiSeq 2500 instrument using a rapid run mode. Reads were trimmed using Skewer^82^, and then aligned to the rhesus macaque genome (BCM Mmul_8.0.1/rheMac8) using Spliced Transcripts Alignment to a Reference (STAR) software^83^, a splice junction aware aligner. Expression estimation was performed using RSEM (RNA-Seq by Expectation Maximization)^84^ and reported as transcripts per million (TPM). To survey miRNA expression, ~ 100 ng of total RNA from each cell type was used as input to generate miRNA sequencing libraries using a QIAseq miRNA library kit (Qiagen). Single-end 100 bp reads (1 × 100) were sequenced on an Illumina HiSeq 2500 using a rapid run mode. To estimate known miRNA abundance, the miRNA-Seq workflow in miARma-Seq was used^85^. Accessions for known miRNAs were obtained from miRbase v22 (October 2018). Library construction and sequencing steps were performed by the University of Wisconsin Biotechnology Center.

### Rhesus macaque in vitro fertilized embryos

Rhesus macaque in vitro fertilized embryos were generated using methods previously described^32,86^. Three blastocyst stage embryos were individually collected and the DNA was amplified from whole embryos using a Repli-G Single Cell kit (Qiagen, cat no: 150343).

### DNA methylation by bisulfite sequencing

DNA was extracted from rhesus fibroblasts and TSCs using a FlexiGene DNA Kit (Qiagen, cat no: 51206) and quantified using a Nanodrop™ One Microvolume UV–Vis Spectrophotometer (ThermoFisher Scientific). The DNA from three embryos, rhesus fibroblasts (n = 1), and TSCs (n = 3 rh121118, rh052318, cy091318) was bisulfite converted using an EZ DNA Methylation-Lightning™ Kit (Zymo Research Corporation, D5030T). PCR was performed to amplify the ELF5 and C19MC gene regions, see Supplementary Table S10 for bisulfite treated DNA primers. PCR products were purified using a QIAquick PCR Purification Kit (Qiagen, cat no: 28104) and were then cloned using a pGEM^®^-T Easy Vector System (Promega, cat no: A1380). For each sample, DNA was isolated from 30 clones using a QIAprep Spin Miniprep Kit (Qiagen, cat no: 27106). Clone DNA was then Sanger sequenced at the University of Wisconsin-Madison Biotechnology Center using the T7 primer of the vector. Methylation marks were identified for each CpG site within the gene region (ELF5 11 CpG sites; C19MC 34 CpG sites) each cell type. ELF5 methylation was analyzed by determine the percentage of clones with methylated CpGs at each site.

### Hormone secretion assays

Cell conditioned media were collected from confluent TSCs or at the completion of cell differentiation and frozen at – 20 °C for subsequent hormone analysis. Cell pellets were also collected in parallel and lysed in 250 µL RIPA buffer (Pierce, ThermoFisher Scientific, cat no: P18990). Protein extracts were diluted in PBS and quantified in duplicate using a Micro BCA Protein Assay Kit (ThermoFisher Scientific, cat no: 23235). Bovine serum albumin (BSA) of known protein concentrations was used as the standard curve to determine protein concentrations for the cell protein extracts. To quantify mCG secretion, a radioimmunoassay was used as previously described^86,87^. Samples were diluted in PBS and run in duplicate. The inter-assay variation (CV) was 6.0%. An enzyme immunoassay (EIA) (Cayman Chemical, cat no: 582601) was used to quantify progesterone secretion in cell conditioned media as previously published^86^. Briefly, samples were diluted in enzyme-linked immunosorbent assay (ELISA) buffer and run in duplicate. A standard curve of known concentration was used to determine the sample concentrations. The minimum level of detection was 0.1 ng/mL. For statistical analyses, samples below the lower limit of detection (0.1 ng/mL) were assigned 0.1 ng/mL. Secretion was normalized to µg cell protein and for statistical analysis a log transformation was performed followed by a nonparametric Kruskal–Wallis test with Dunn’s correction was used to test significance (p < 0.05).

### Flow cytometry

Cryopreserved cells were thawed at room temperature and washed twice with 10 mL of FACS buffer (PBS and 2% FBS) and filtered through a 100 µM filter (BD BioSciences, cat no: 352360). Cell concentrations were determined using a hemocytometer. Viability was assessed by staining with Ghost Red 780 (Tonbo Biosciences, cat no: 13-0865) dye for 30 min at 4 °C. Cells were then washed and stained for surface cell markers, 25D3 (Mamu-AG) Alexa 647, Mamu-E PE and CD-56 PE-CF594 for 20 min at room temperature and then washed, see Supplementary Table S11 for flow cytometry antibody information. In house antibody 25D3 (Mamu-AG)^29^, was conjugated to Alexa 647 using the Biotium Mix-n-Stain CF 647 kit (Biotium Inc, cat no: 92238) and the Mamu-E antibody (MEM-E/06^28^) was conjugated to PE using the Biotium Mix-n-Stain R-PE kit (Biotium Inc, cat no: 92298) following the manufacturer’s protocol. Cells were then fixed and permeabilized using the Foxp3 transcription factor staining buffer kit (eBioscience, cat no: 00-552-3-00) according to the manufacturer’s guidelines. Fixation and washing were followed by intracellular staining using the following antibodies: Ki-67 Brilliant Violet 711, Cytokeratin 7/8 Brilliant Violet 421, Vimentin Alexa Flour 488. Intracellular staining was done for 30 min at 4 °C. Unstained cells, control cell lines and compensation controls using cell lines or UltraComp eBeads (eBioscience, cat no: 01-2222-42) were prepared in parallel. TSCs and EVTs from 7 lines were analyzed concurrently on a five laser BD LSRII instrument. One replicate of stained rhesus fetal fibroblasts and unstained TSCs and EVTs were also analyzed. Positive staining was confirmed using positive and negative control cell lines. Results were analyzed using FlowJo v10.6 software (FlowJo LLC). Cells were gated to eliminate doublets and dead cells. The median fluorescent intensity was calculated for live, single cells in each channel.

### MMP2 secretion

A monkey MMP2 ELISA (Abcam, cat no: ab269561) was used to quantify MMP2 secretion in TSC and EVT conditioned media following the manufacturers protocol. TSC media were collected after 48 h of culture with cell confluency ranging from 60–90%, and similarly, EVT media were collected on day 8 of differentiation after 48 h of conditioning. Conditioned media were spun at 500 × *g* for 5 min to remove dead cells and debris and then 1 mL of medium for each sample was filtered through a 0.22 µM syringe filter. Samples were diluted in Sample Diluent NS, run in duplicate, and absorbance was measured at 450 nm. A standard curve of known MMP2 concentrations was used to determine the presence of MMP2 in the samples.

### Single cell RNA-sequencing

Cryopreserved passage 2 and passage 10 TSCs of rh121118 were thawed and cultured for 72 h. TSCs were then lifted with TrypLE by incubation for 30 min at 37 °C. An equal volume of DMEM/F12 was then added and cells were triturated by passage through various sizes of pipette tips. The cell suspension was pelleted, supernatant removed and resuspended in 1 mL DMEM/F-12 and cells were then strained through a 10 µm cell strainer followed by additional trituration. Single cell suspensions were then evaluated for viability and cell count by using a Countess II FL Automated Cell Counter (Invitrogen, ThermoFisher). Cells were then diluted to a concentration of 600 cells/µL for a target cell range of ~ 5000 cells to be individually encapsulated into nanoliter-scale droplets containing reverse transcription reagents and uniquely barcoded Gel Bead-in EMulsions (GEMS). cDNA quality was assessed using an Agilent HS DNA chip and libraries were prepared using a Chromium Single Cell 3′ v2 kit (10 × Genomics, USA). Libraries were quantified using a Qubit Fluorometer (Invitrogen) with a Qubit dsDNA HS kit (Invitrogen, ThermoFisher Scientific, cat no: Q33230) and evaluated for quality by sequencing on a MiSeq Nano platform (Illumina). Libraries were then sequenced across two lanes of a Novaseq 6000 SP flow cell (lllumina).

Single-cell RNA sequencing (scRNA-seq) data was analyzed by the UW Bioinformatics Resource Center. Sequencing data was demultiplexed using the Cell Ranger Single Cell Software Suite, mkfastq command wrapped around Illumina's bcl2fastq. The MiSeq balancing run was quality controlled using calculations based on UMI-tools^88^. Samples’ libraries were balanced for the number of estimated reads per cell and run on an Illumina NovaSeq system. Cell Ranger software was then run to perform demultiplexing, alignment, filtering, barcode counting, UMI counting, and gene expression estimation for each sample according to the 10 × Genomics documentation (https://support.10xgenomics.com/single-cell-gene-expression/software/pipelines/latest/what-is-cell-ranger). Reads were aligned to the rhesus macaque reference genome MacaM_v7.8.2. The gene expression estimates from each sample were then aggregated using Cellranger (cellranger aggr) to compare experimental groups with normalized sequencing-depth and expression data. Cell expression profiles were analyzed by K-means clustering using Davies-Bouldin Index to identify the ideal number of preset number of clusters. The Louvain Modularity Optimization (LMO) algorithm^65^ was used to cluster cells on the number of nearest neighbors (based on cellular gene expression levels) and the clusters were plotted using a dimensionality reduction method, t-Stochastic Neighbor Embedding (t-SNE). t-SNE plot hierarchically clusters the nearest neighbors in 2-dimensional space^89^. For each t-SNE cluster, the differential expression algorithms are run to compare to all other cells, yielding a list of differentially expressed genes in that cluster compared to the rest of the sample. The up or down regulated significantly differentially expressed genes (p < 0.05, > twofold change expression) were imputed into the web-based tool, ENRICHR^90,91^, to identify Human KEGG pathways and Gene Ontology Biological Processes altered by the gene set.

## Supplementary information

Supplementary Information.
