# Supplementary material for: Placenta-derived macaque trophoblast stem cells: differentiation to syncytiotrophoblasts and extravillous trophoblasts reveals phenotypic reprogramming
Source: Sci Rep. 2020 Nov 5;10:19159. doi: 10.1038/s41598-020-76313-w (PMC7644694; doi:10.1038/s41598-020-76313-w)
Supplement: Supplementary file 1 — Supplementary Information. [file 41598_2020_76313_MOESM1_ESM.docx]

**Placenta-derived macaque trophoblast stem cells: differentiation to syncytiotrophoblasts and extravillous trophoblasts reveals phenotypic reprogramming**

Jenna Kropp Schmidt^1*^, Logan T. Keding^2^, Lindsey N. Block^1^, Gregory J. Wiepz^2^, Michelle R. Koenig^2^, Michael G. Meyer^1^, Brittany M. Dusek^1^, Kamryn M. Kroner^1^, Mario J. Bertogliat^1^, Avery R. Kallio^1^, Katherine D. Mean^1^ and Thaddeus G. Golos^1,2,3^

^1^Wisconsin National Primate Research Center, University of Wisconsin-Madison

^2^Department of Comparative Biosciences, University of Wisconsin-Madison

^3^Department of Obstetrics and Gynecology, University of Wisconsin-Madison

*Corresponding Author

Jenna Kropp Schmidt

Wisconsin National Primate Research Center

1220 Capitol Ct.

Madison, WI 53715-1299

jkropp@wisc.edu

**Supplemental Figure S1.** Time course of TSC and *in vitro* differentiation of pri-ST cultures. Immunocytochemistry for Ki-67 (pink, nuclear) and nuclear stain, DAPI (blue). Ki67 expression is lost within 48h of culture as pri-ST differentiate. Scale bars in all panels represent 100 µm.

**Supplemental Figure S2.** IgG control immunostaining of cultured cells used in this study.

**Supplemental Figure S3.** Extravillous trophoblast (EVT) morphology by phase contrast imaging. Representative images from EVTs derived from three TSC lines across 8 days in EVT differentiation medium. Scale bars represent 500 µm.

**Supplemental Figure S4**. t-SNE distribution of cellular gene expression determined by scRNA-seq at passage 2 (p2, A) and passage 10 (p10, B). The number of significantly up or down regulated genes in panel B appear in parentheses next to the cluster number. No genes were significantly differentially expressed across the clusters of passage 2 cells.

**Supplemental Figure S5.** scRNA-seq expression of individual genes within the t-SNE distribution. Top differentially expressed genes in common between clusters identified by K-means clustering analysis (A) or LMO algorithm (B).

**Supplemental Figure S6.** Gel electrophoresis images of RT-PCR products from Figure 3A. A 100 base pair (bp) step-wise ladder was used with 500 bp mark indicated on the gel image. All lanes in which a product was loaded are labeled by cell type and gene. No cDNA control and no reverse transcriptase control reactions were run in parallel for each cell type and primer pair (not shown).

Supplemental Figure S1

72 h

24 h

48 h


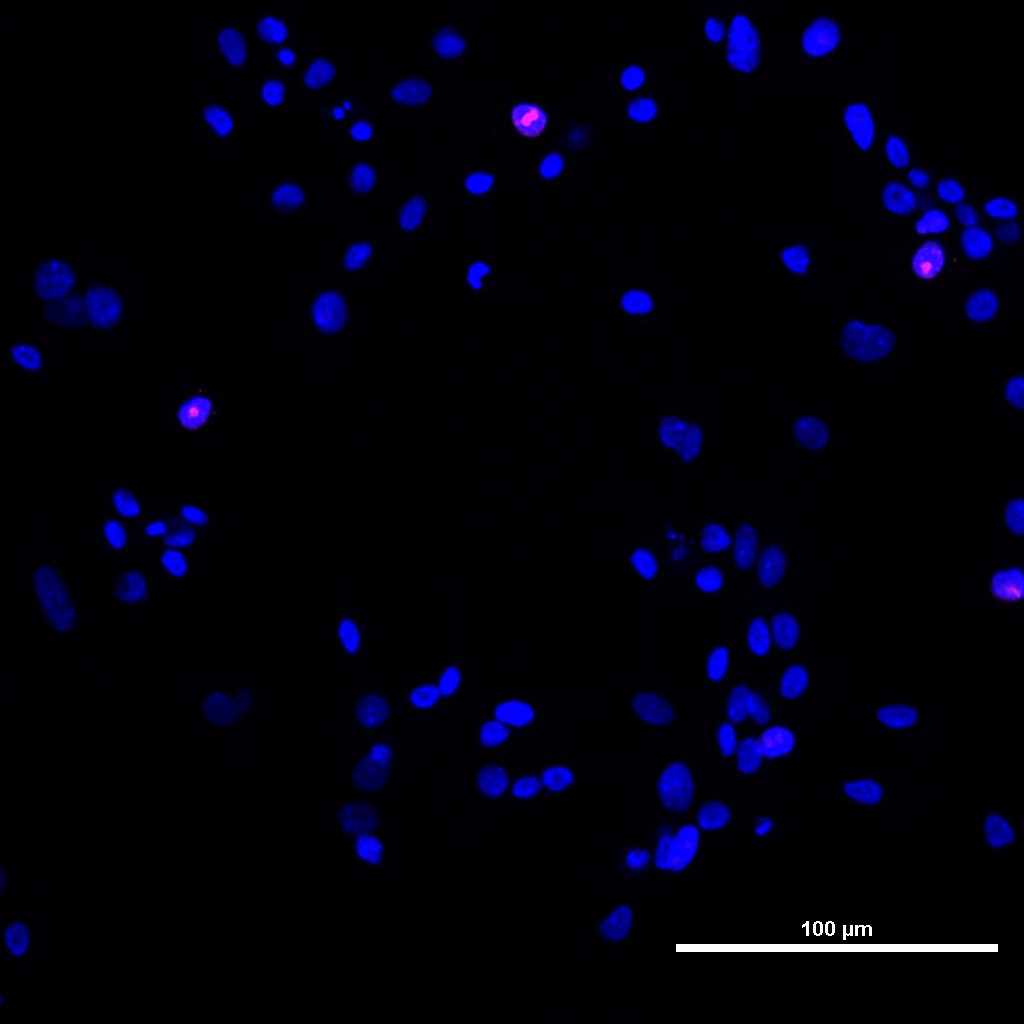

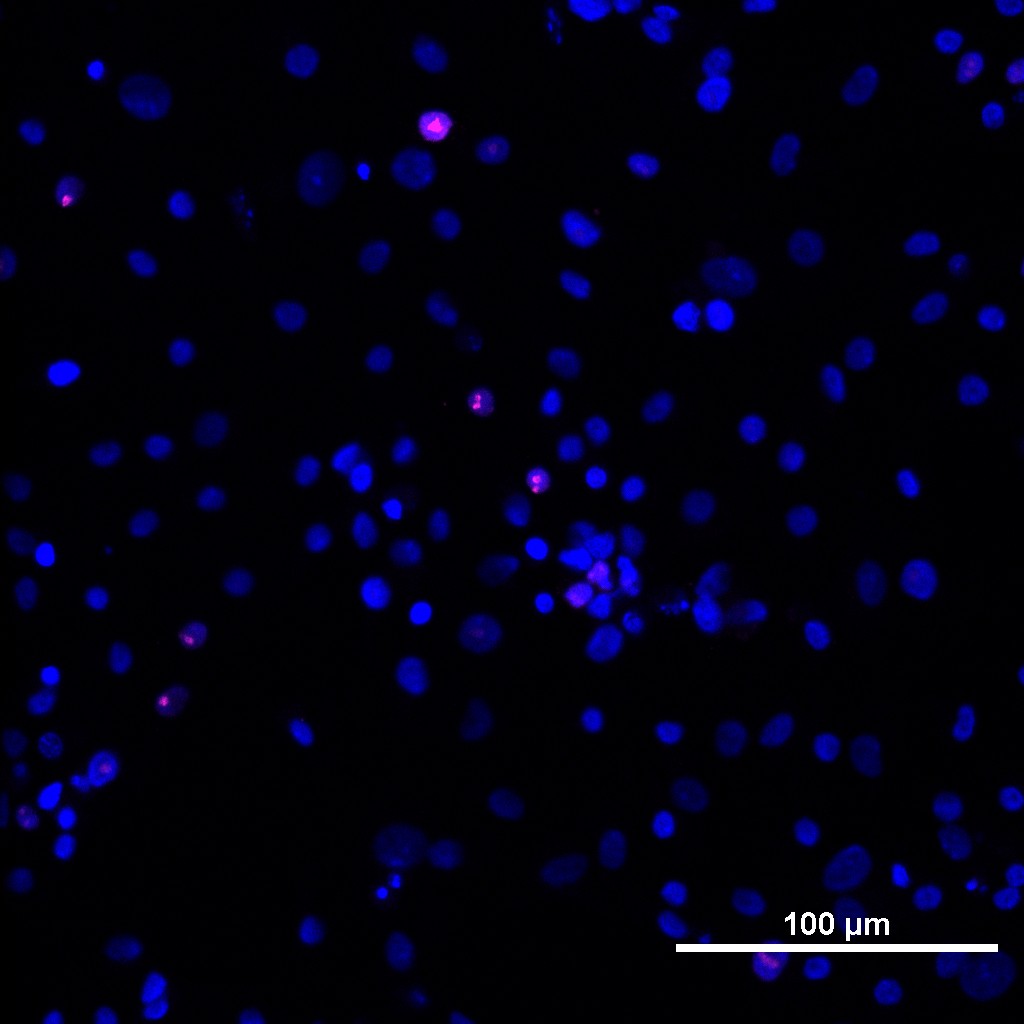

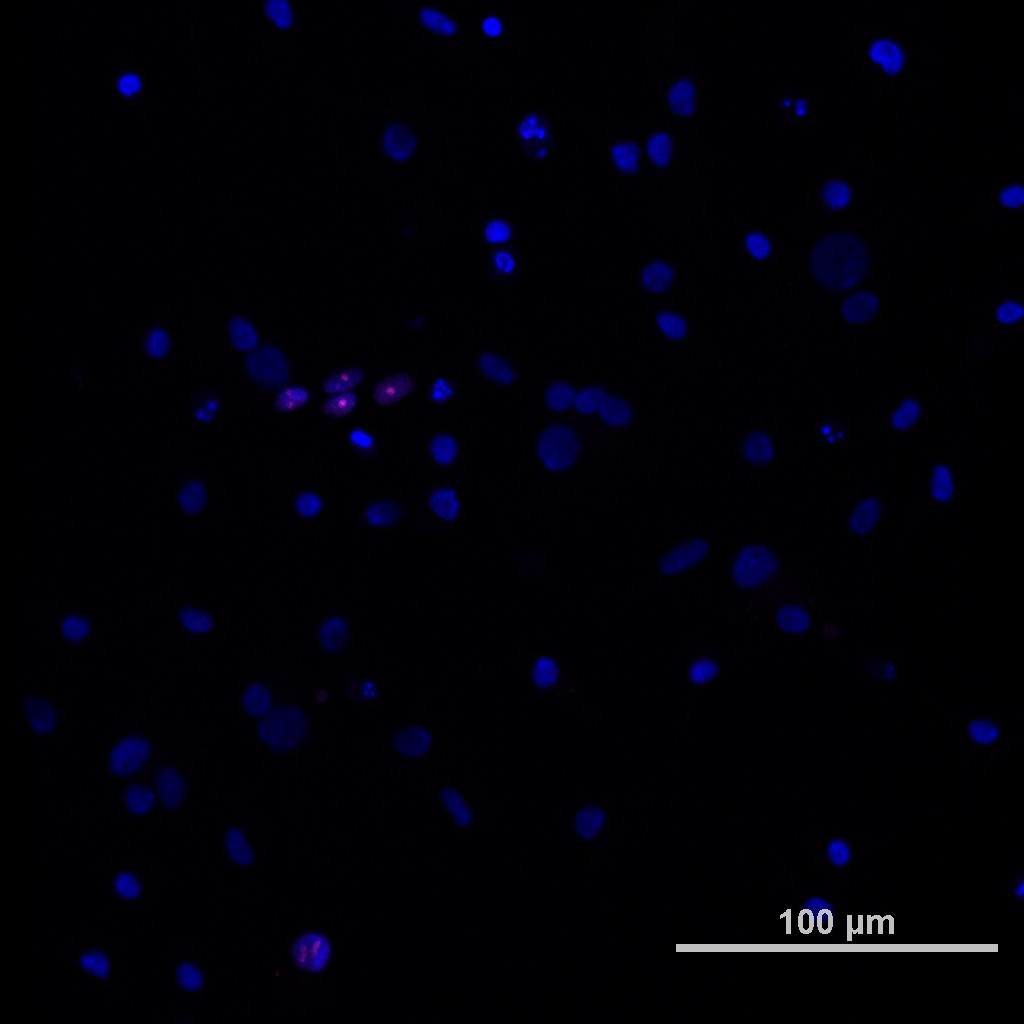


TSC

TSC


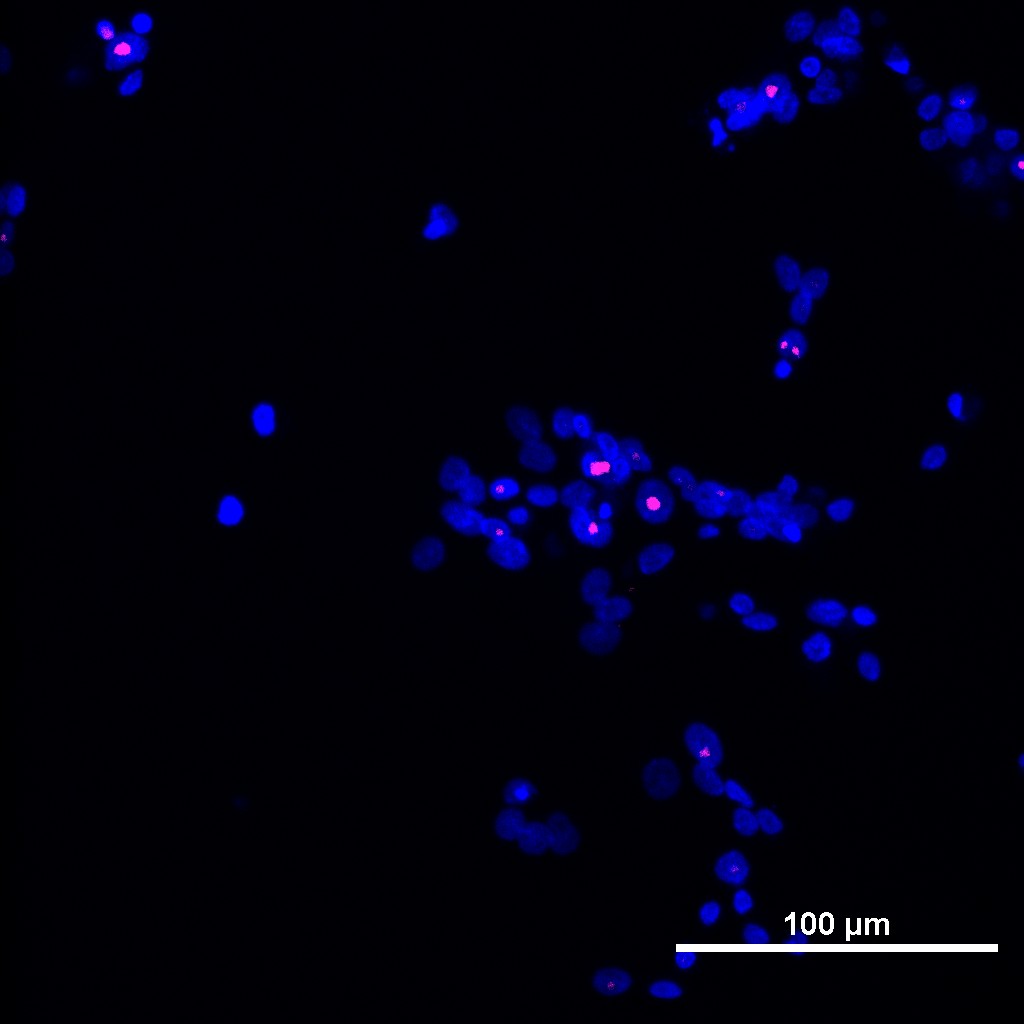

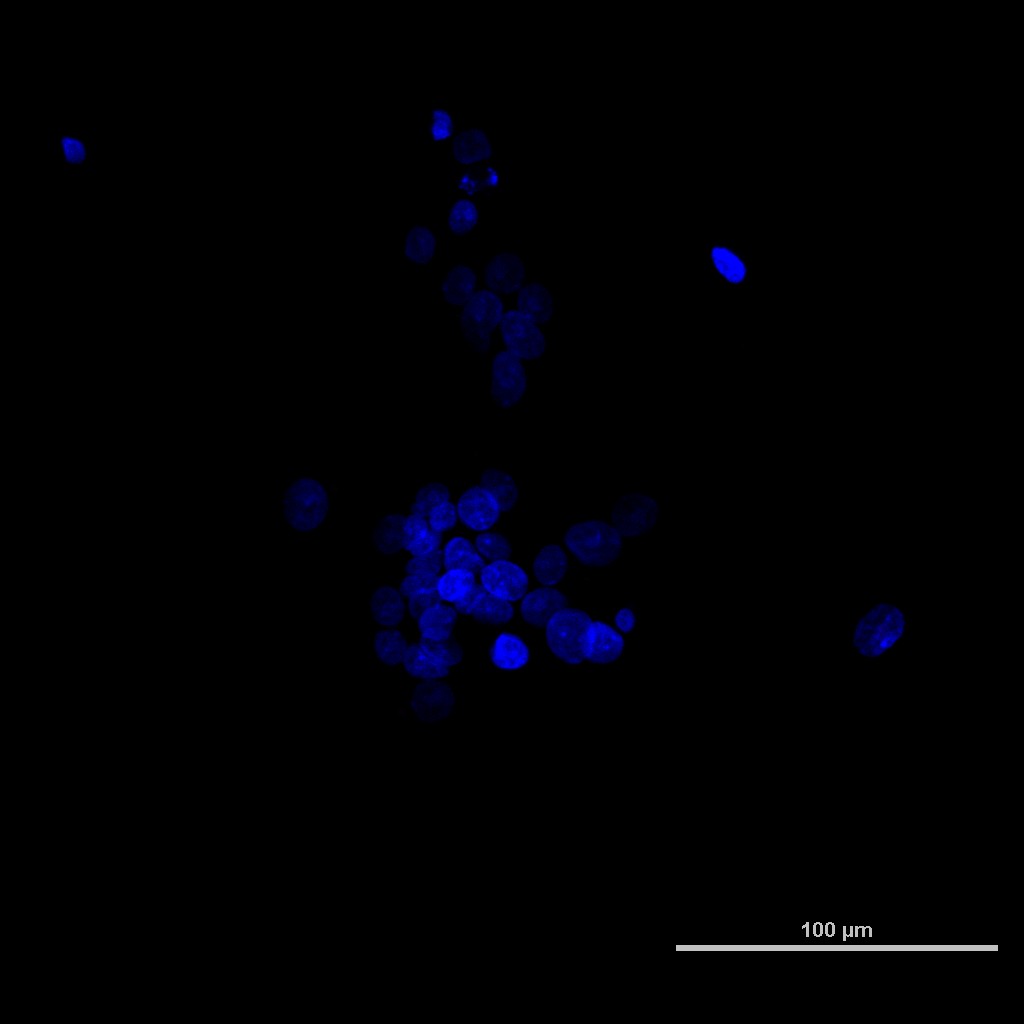

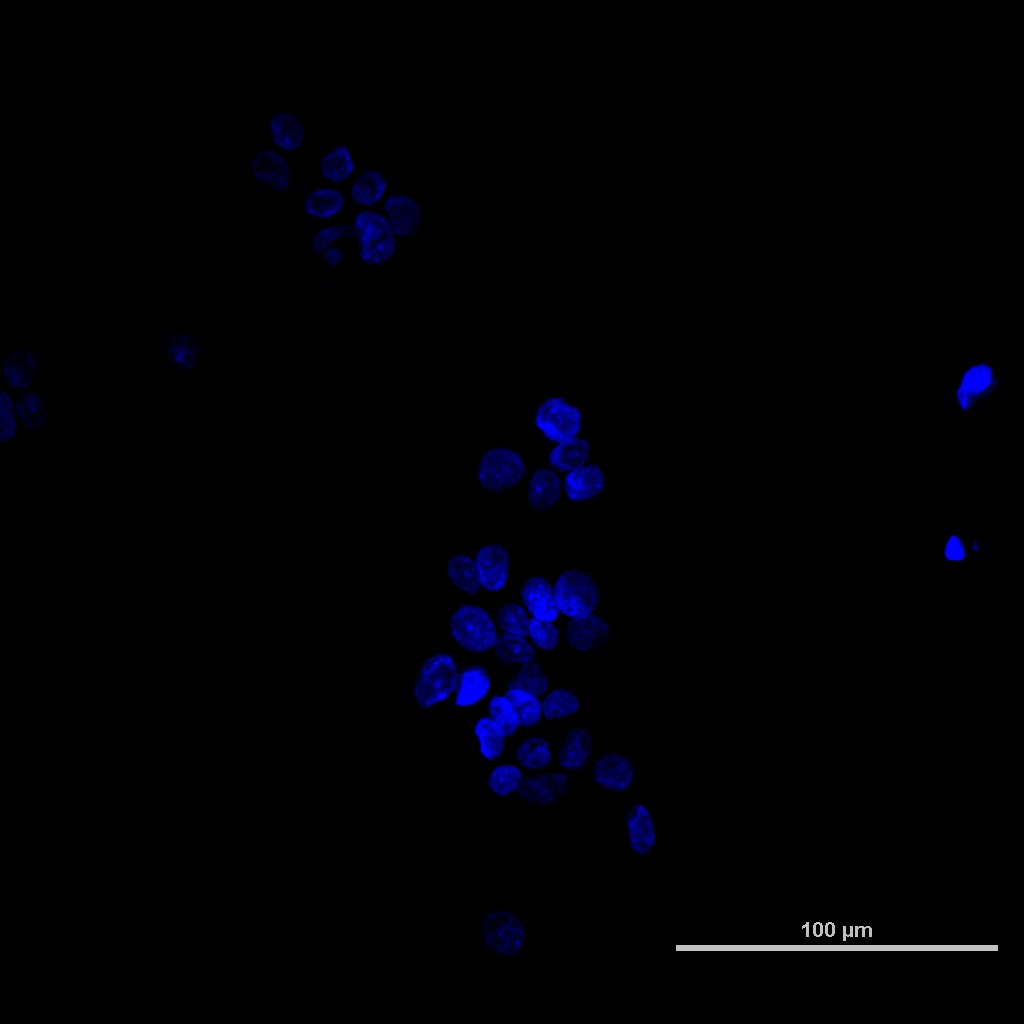


pri-ST

pri-ST


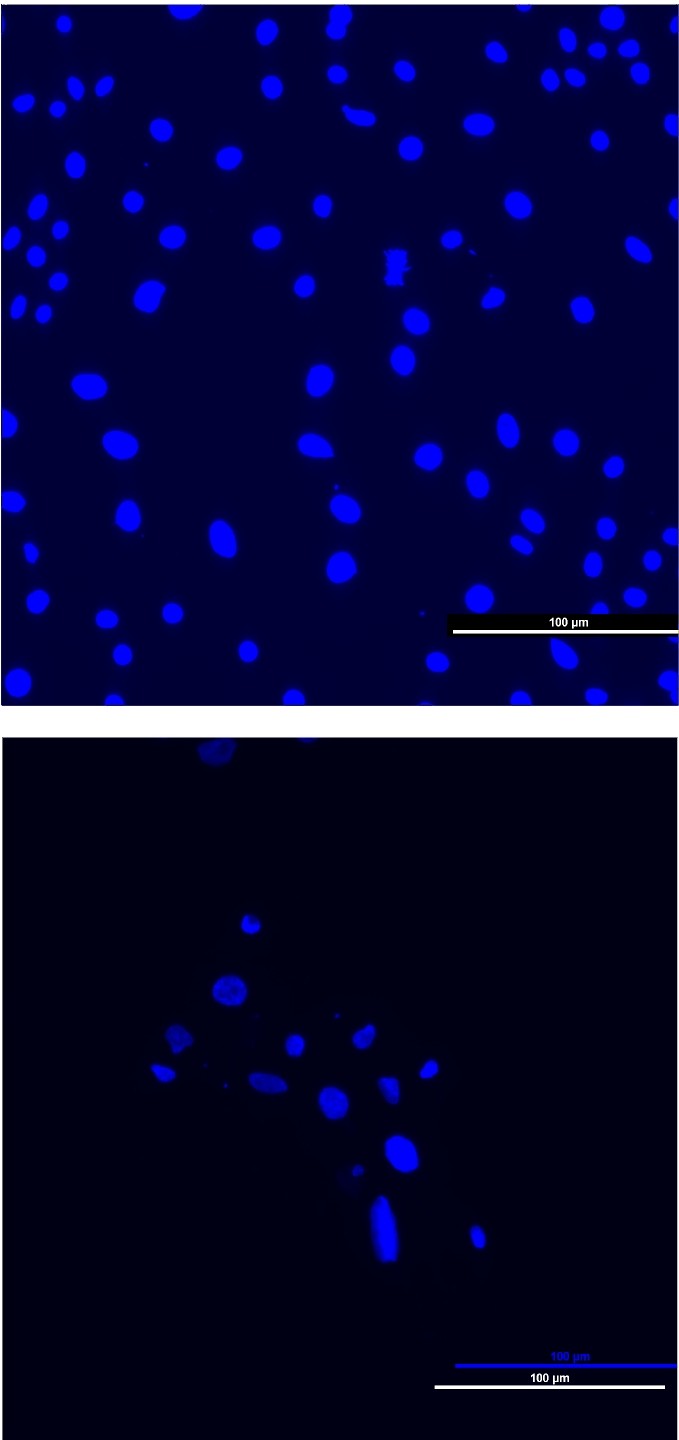

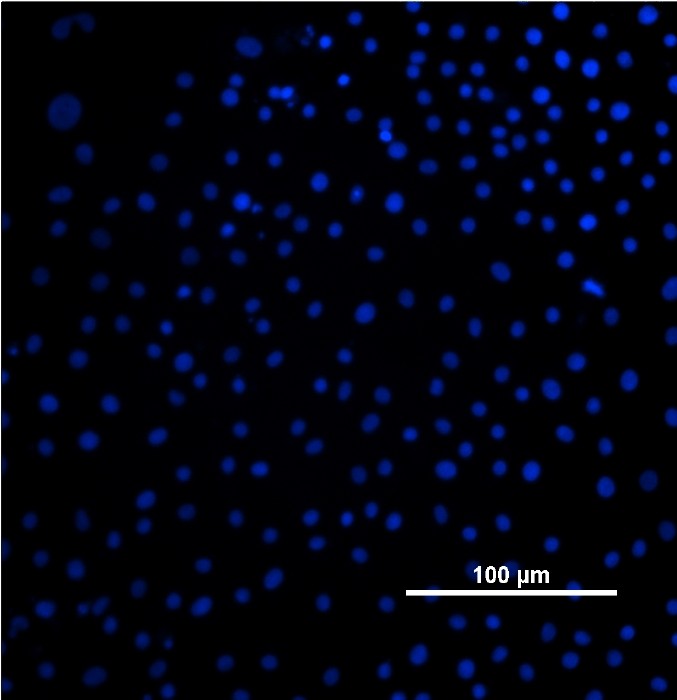

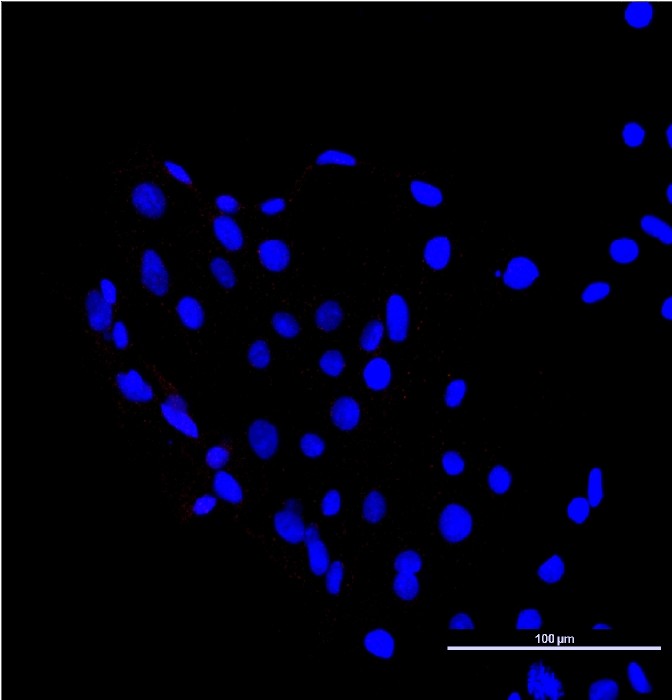

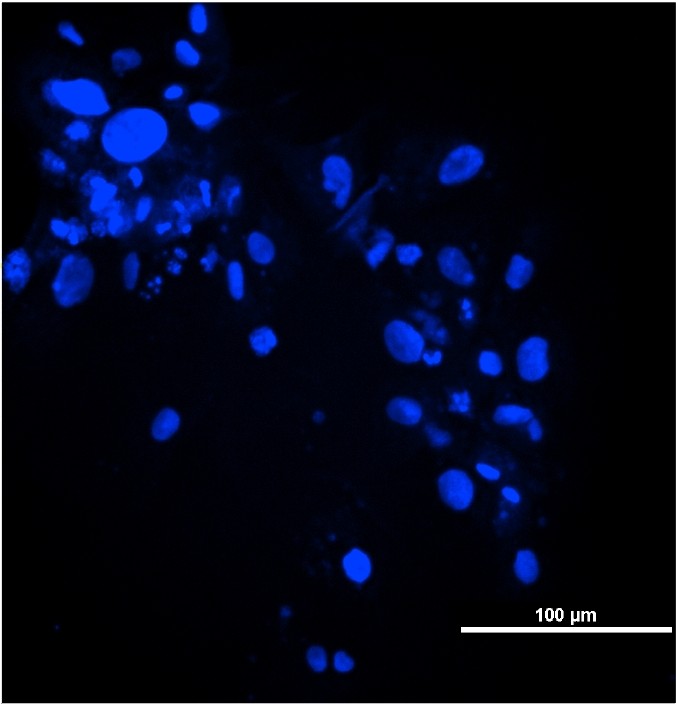

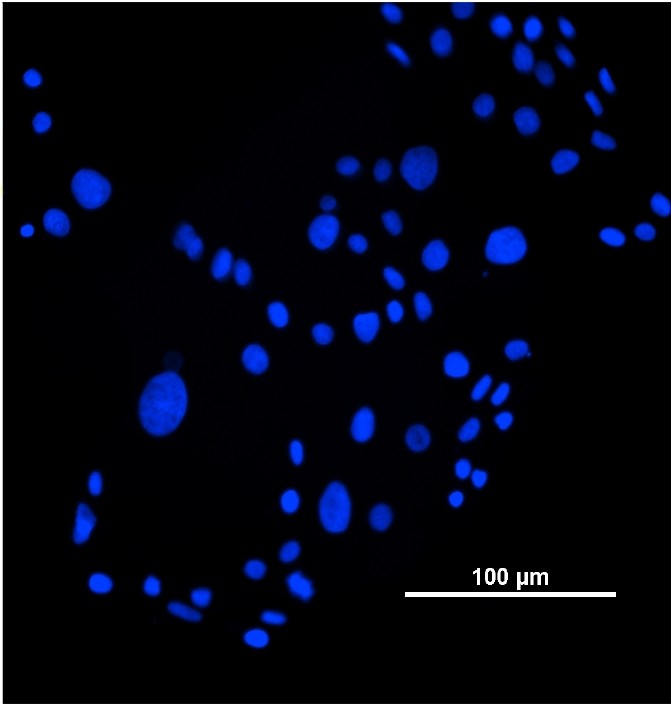

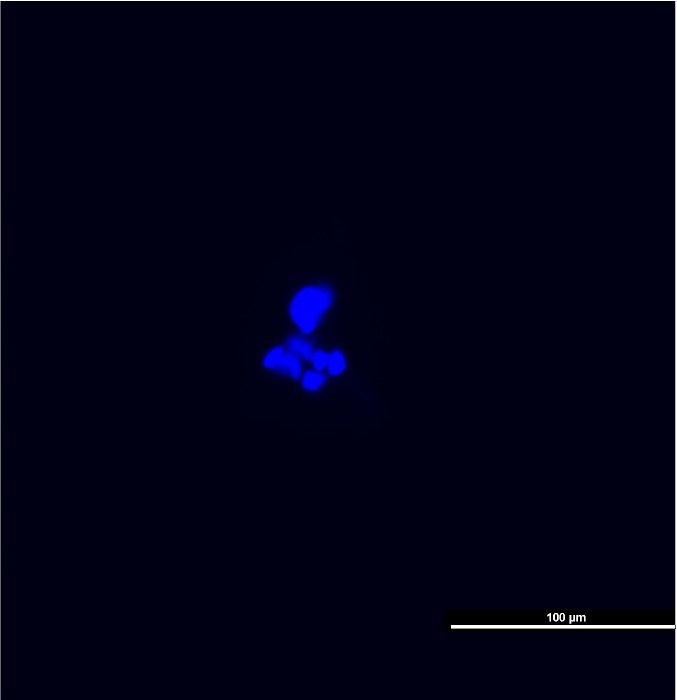

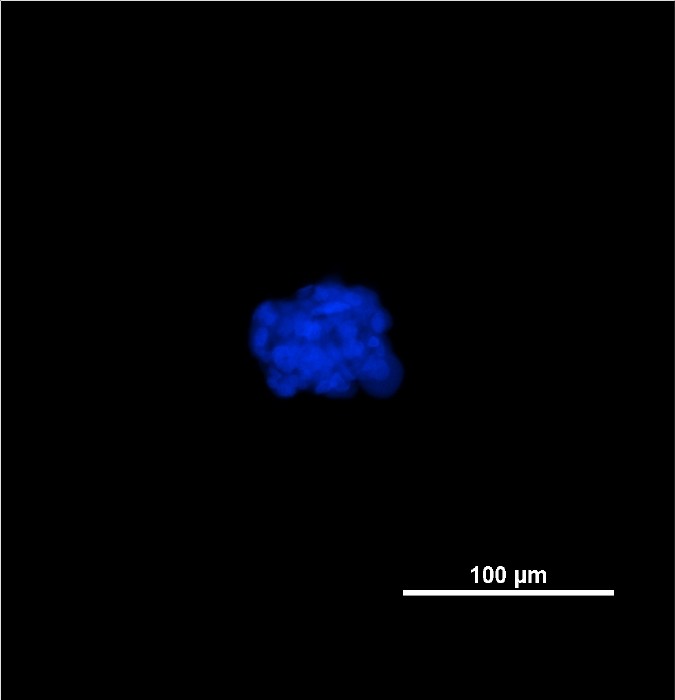

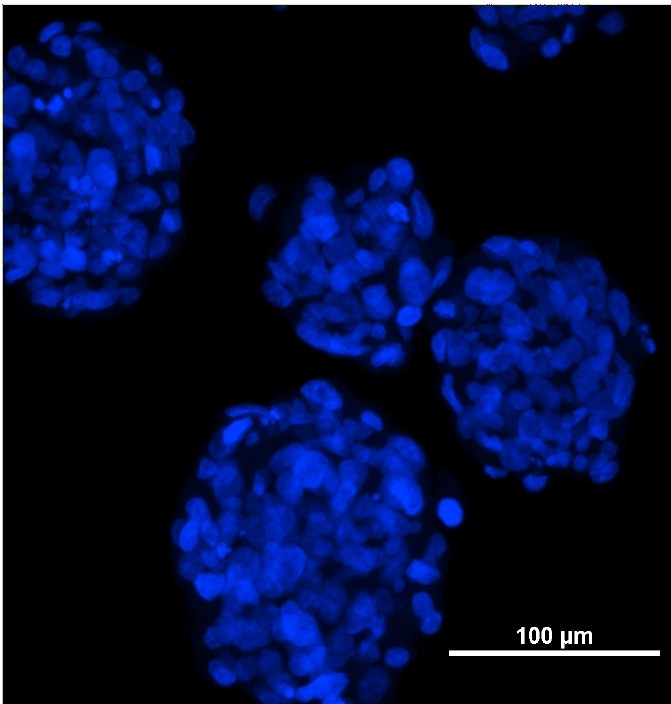

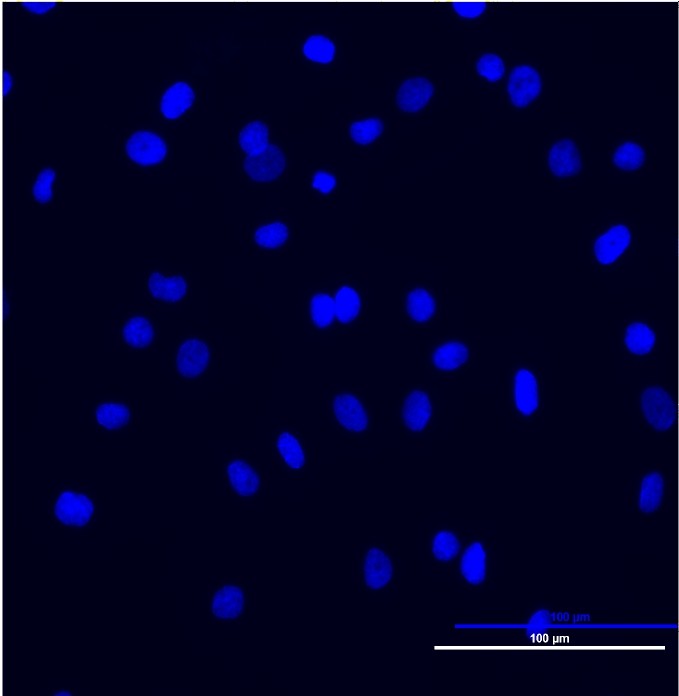

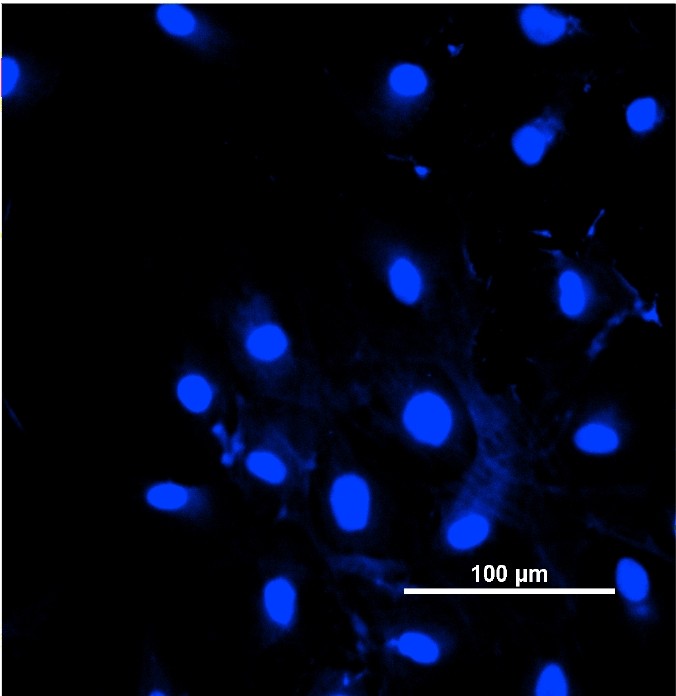

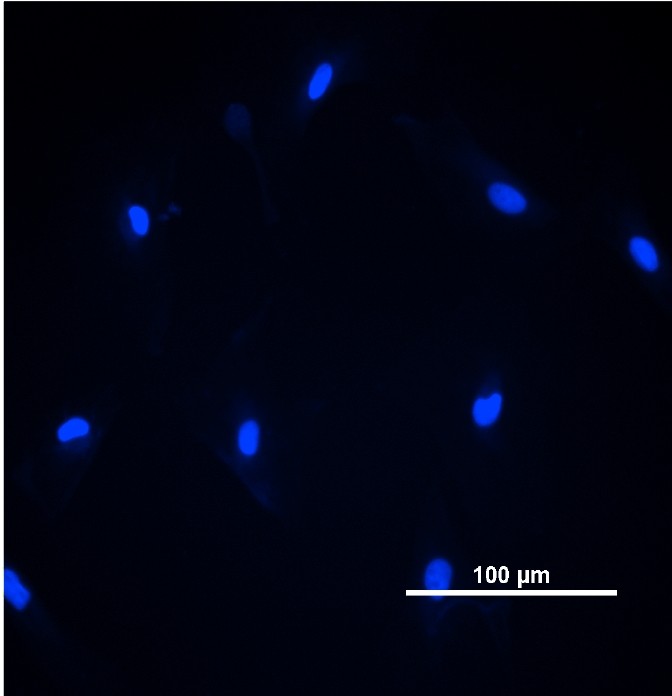


rab-IgG

ms-IgG2A

ms-IgG1

Supplemental Figure S2

TSC

TSC

ST-2D

ST-2D

ST-3D

ST-3D

fibroblast

Fibroblast

Supplemental Figure S3

day 8

day 6

day 3

rh052318

rh010319

rh020119


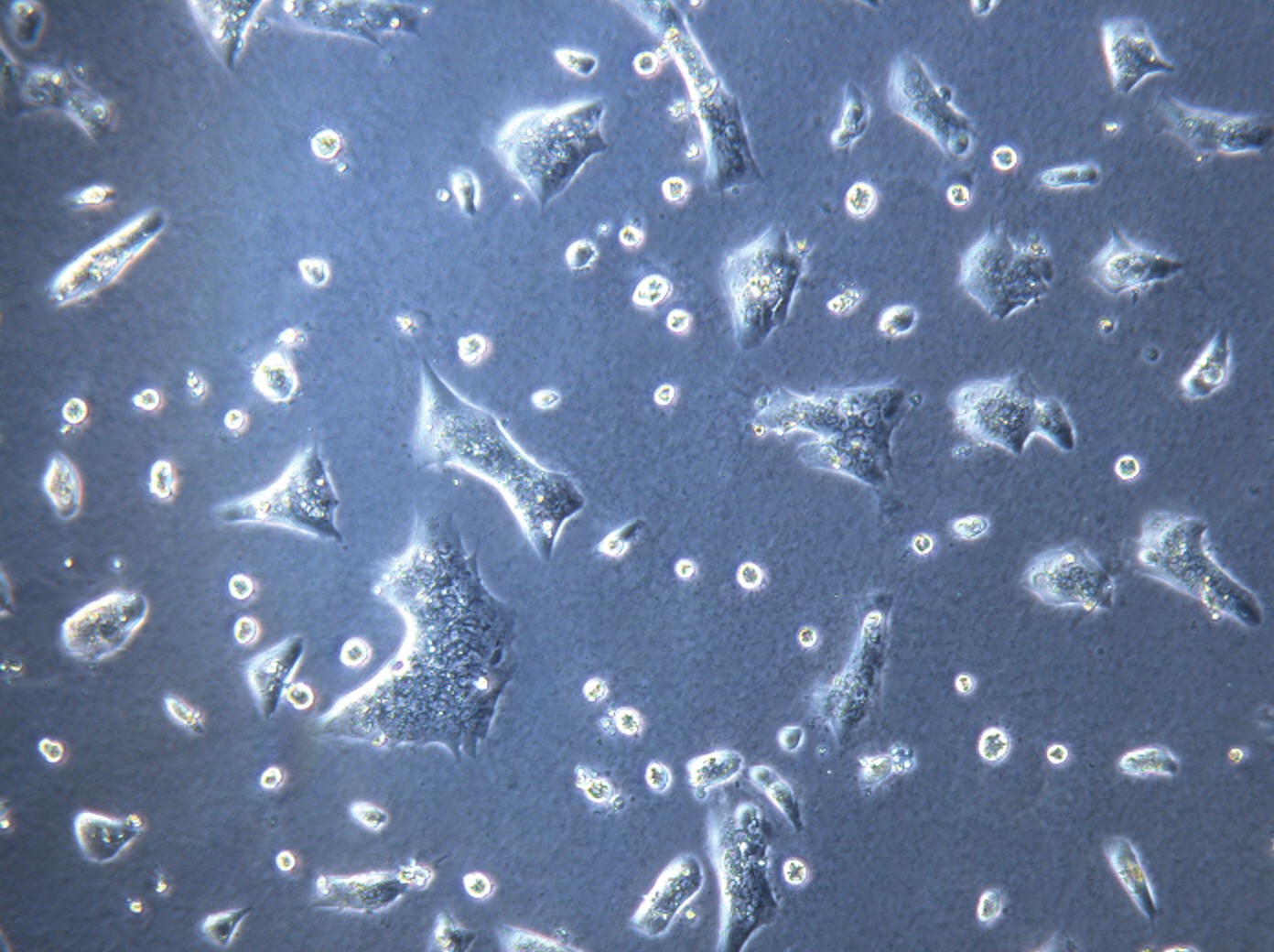

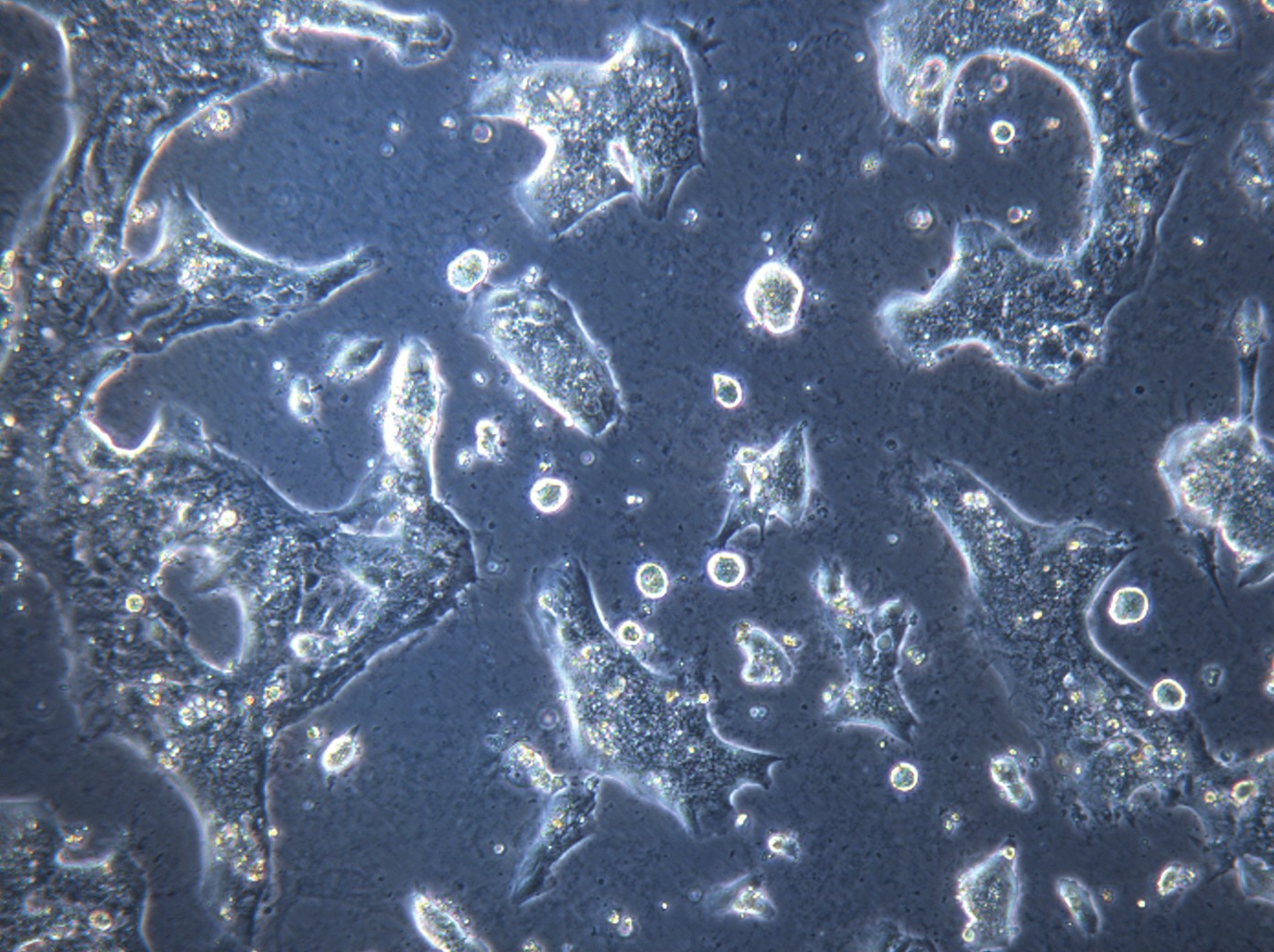

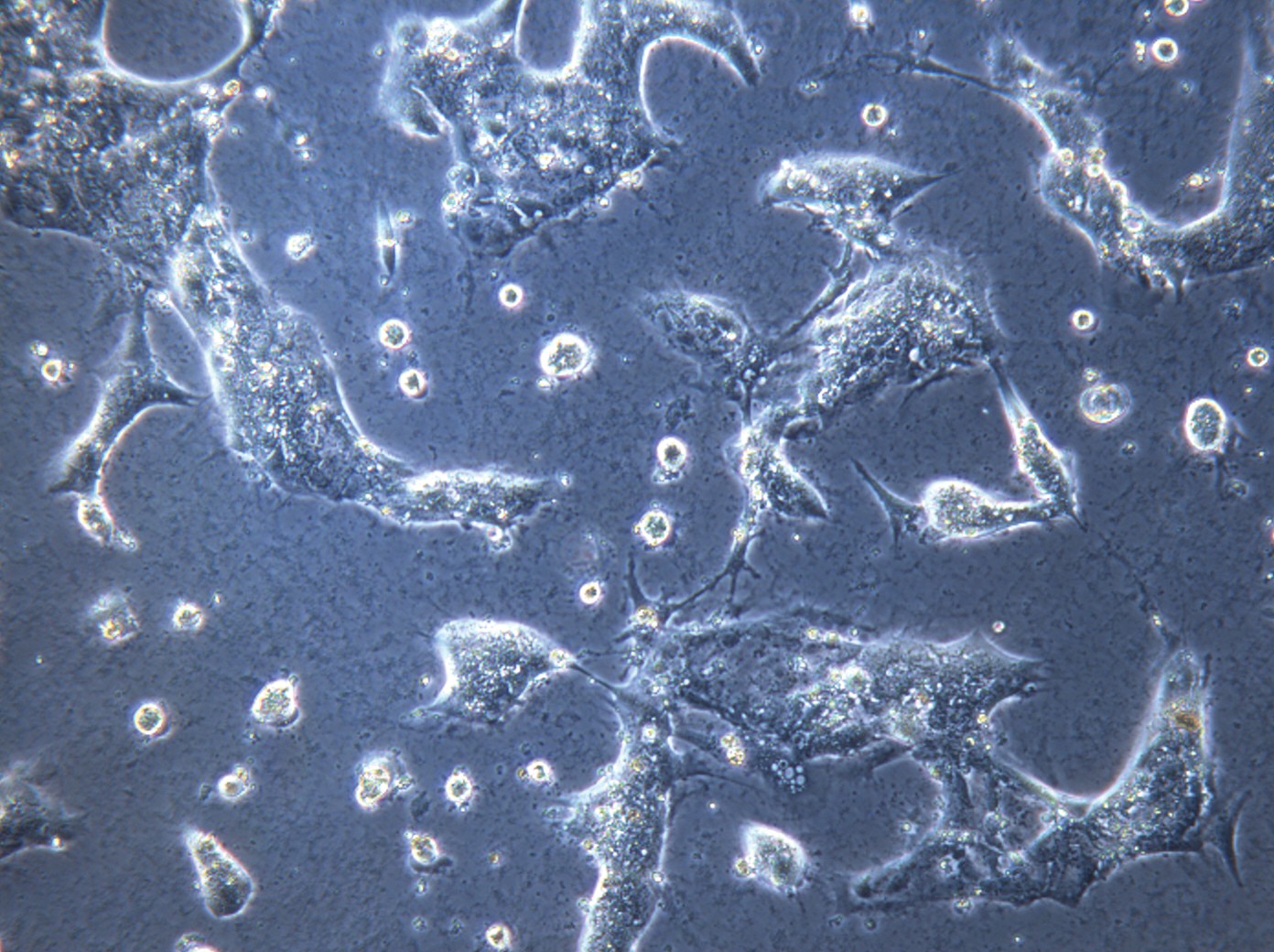

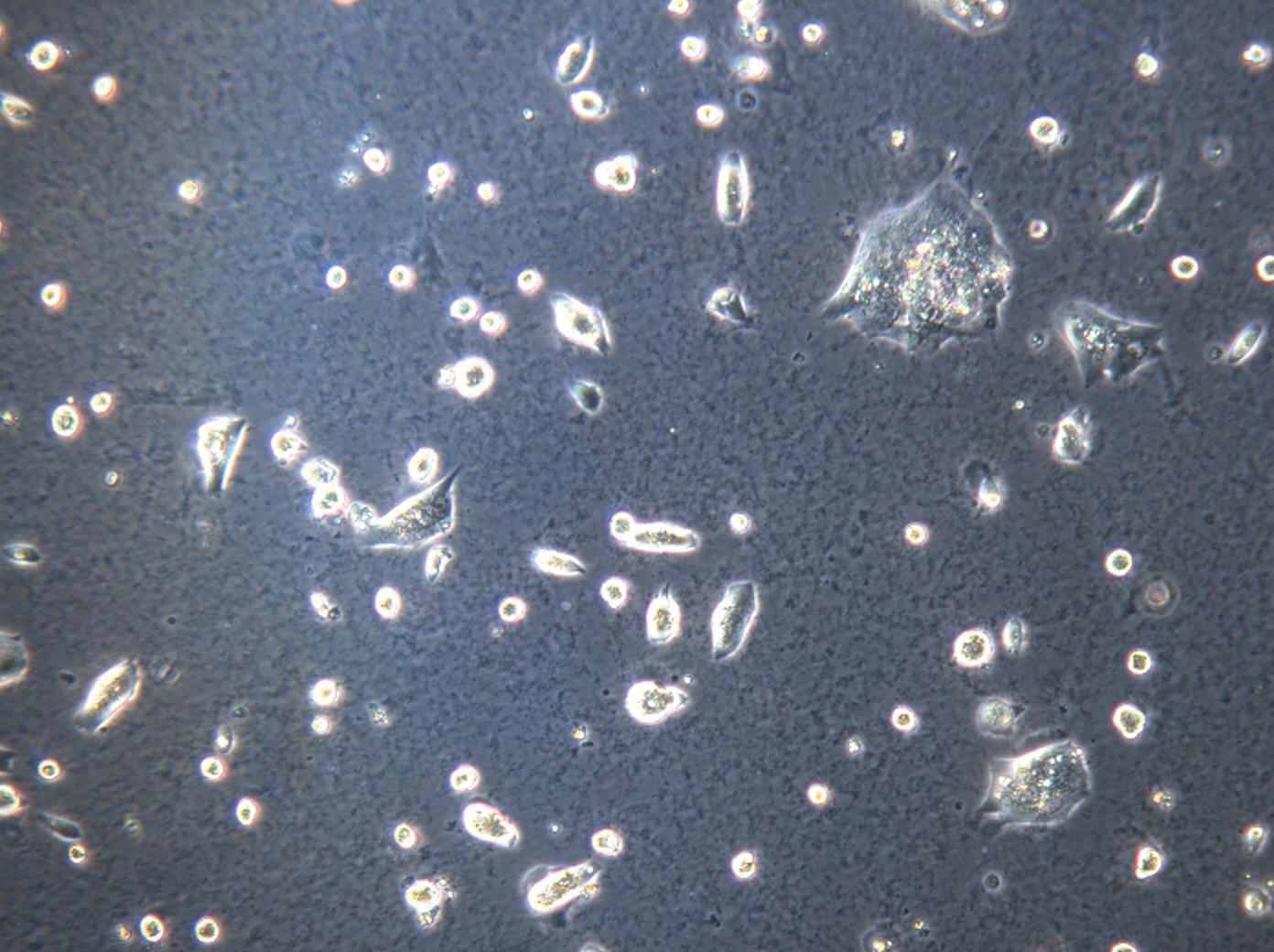

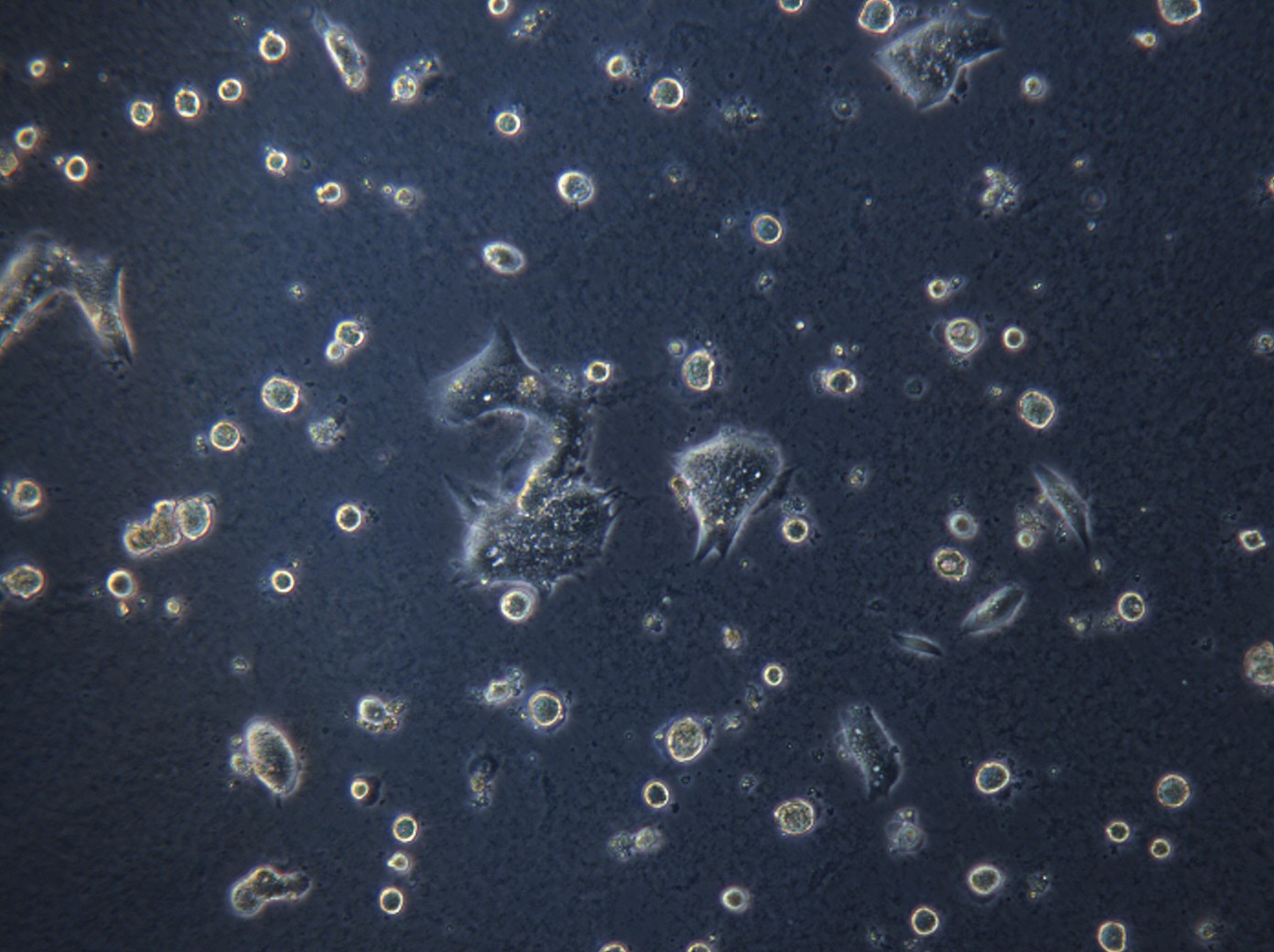

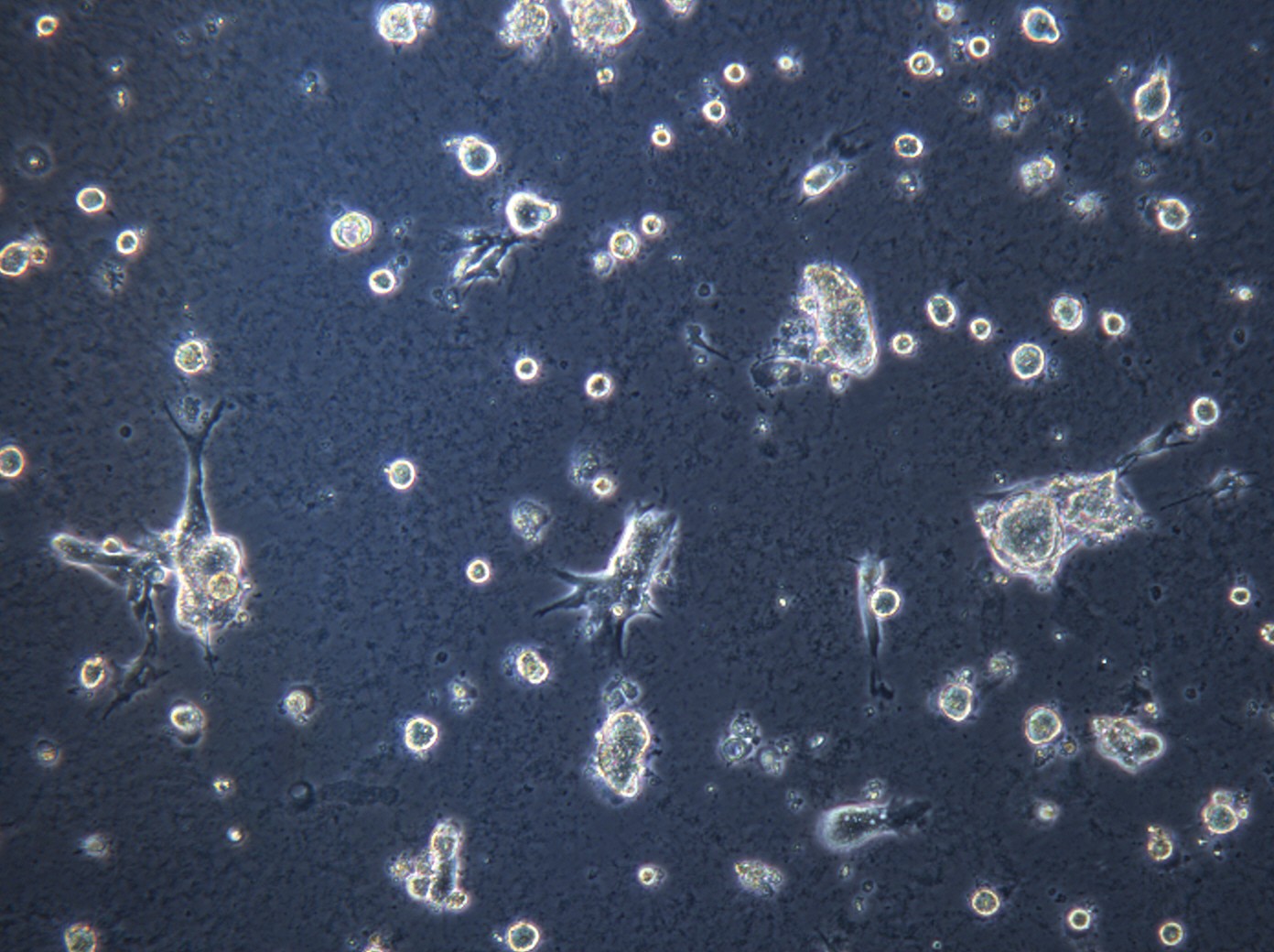

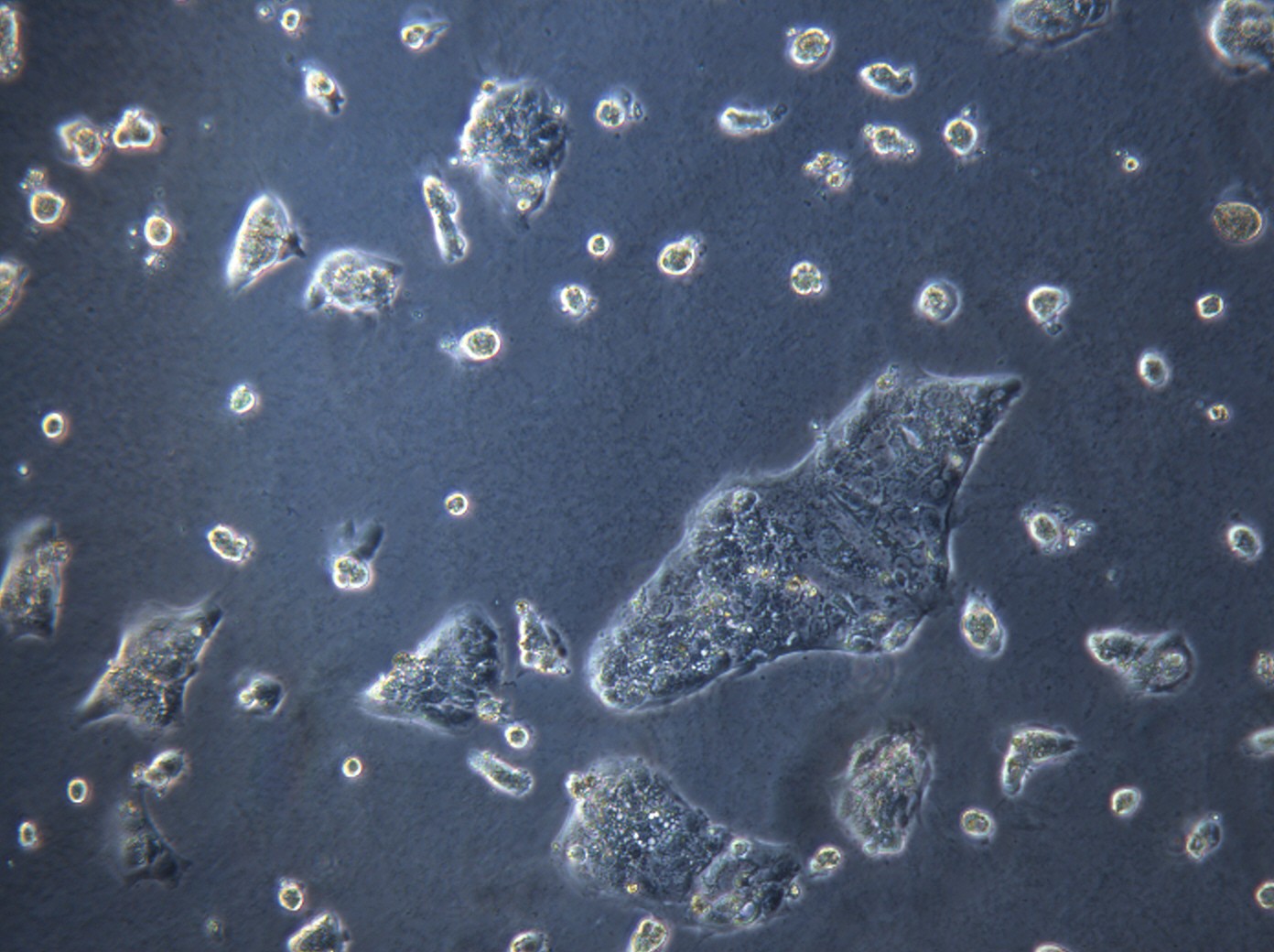

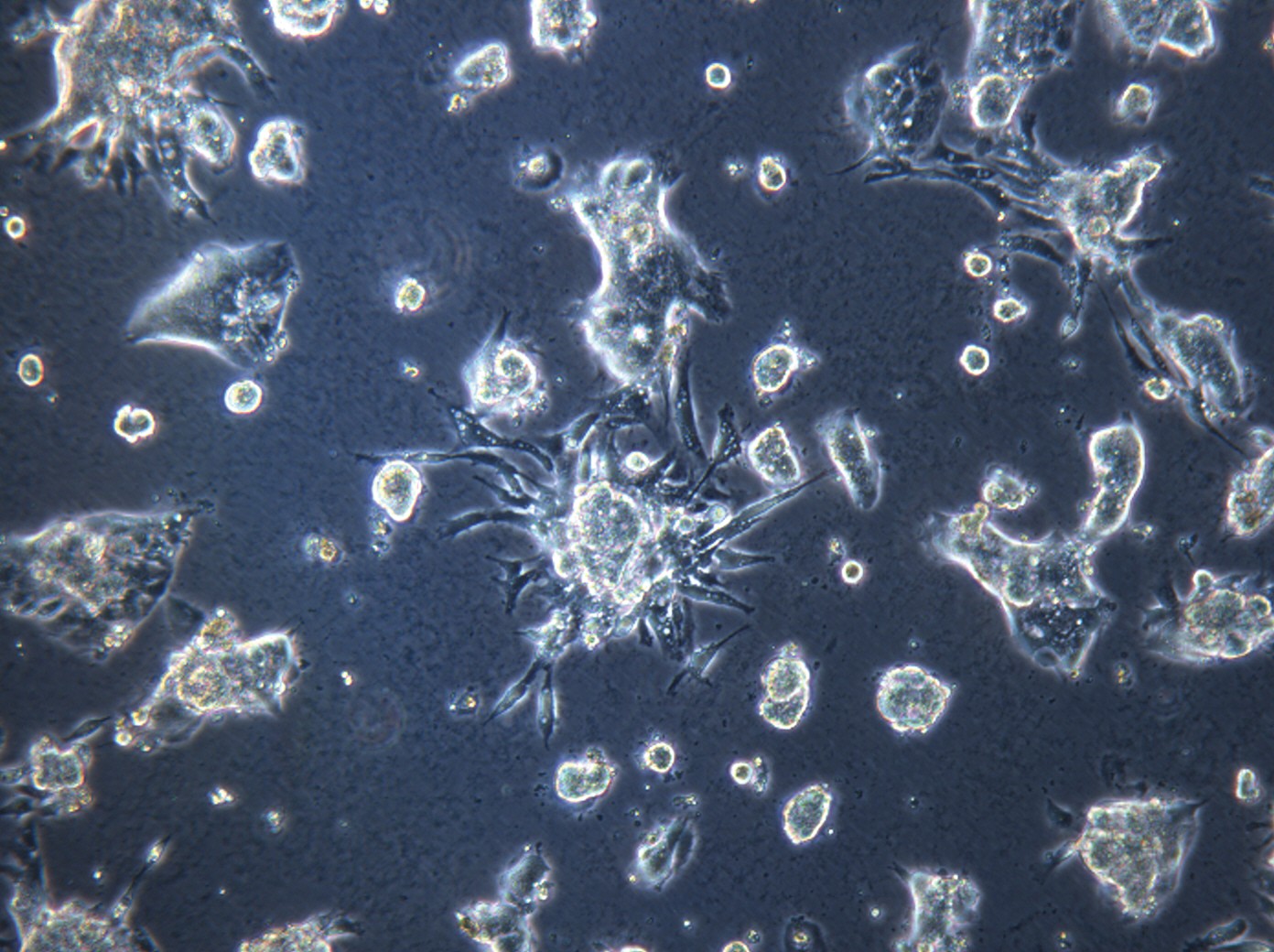

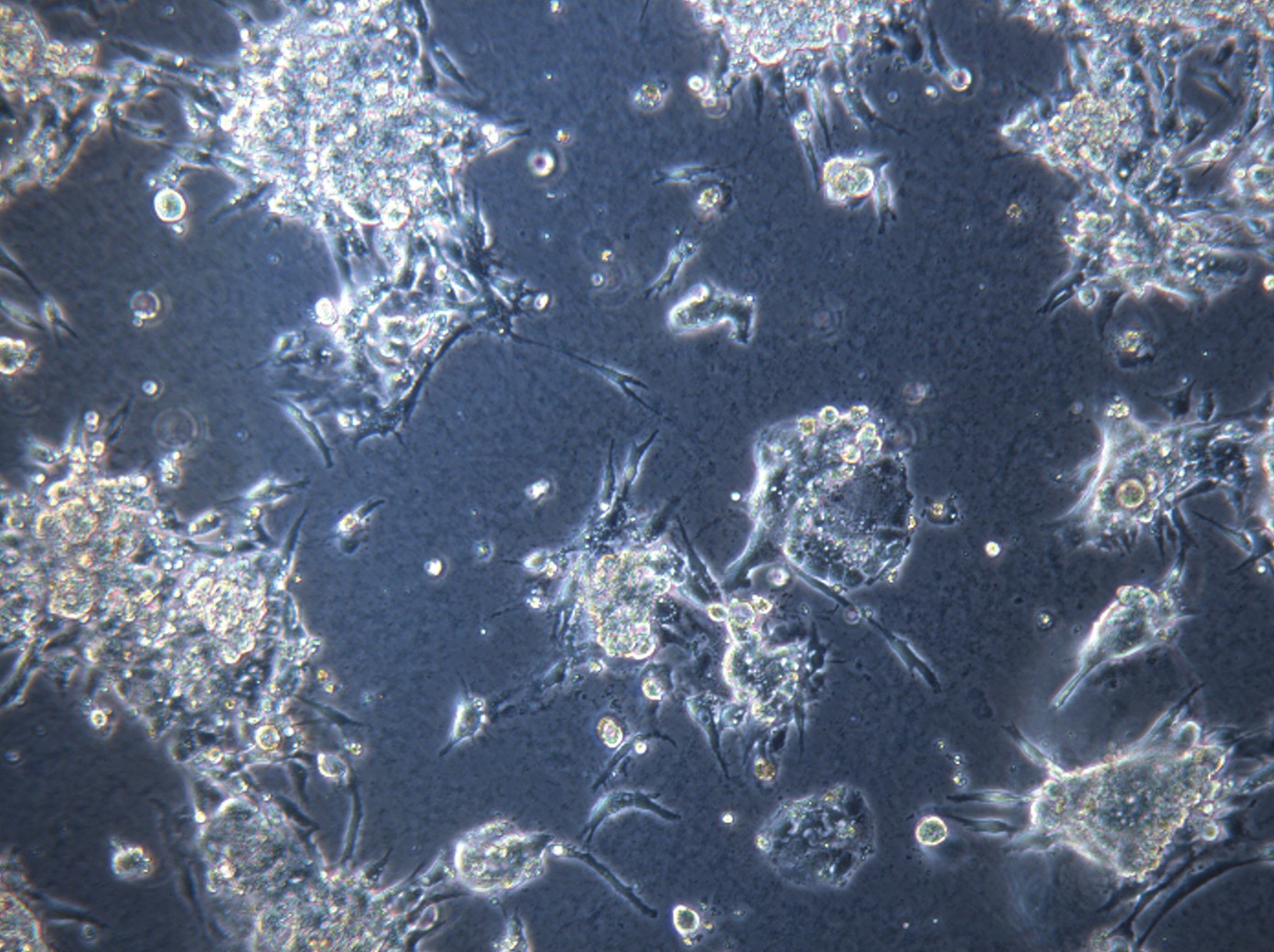


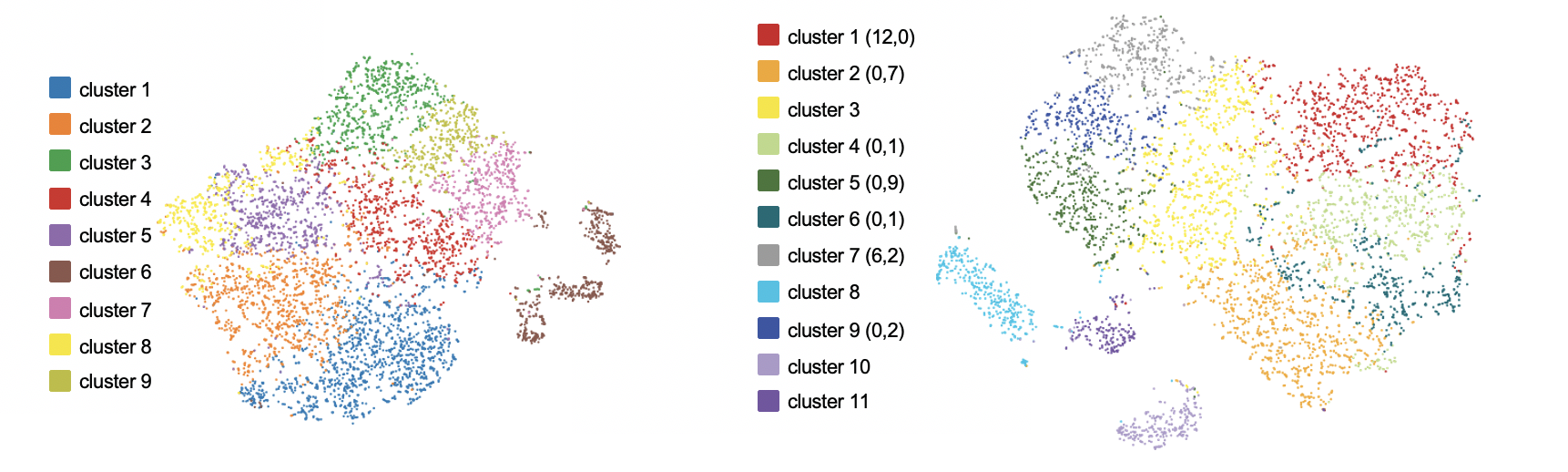


Supplemental Figure S4

passage 2

passage 10

B.

A.


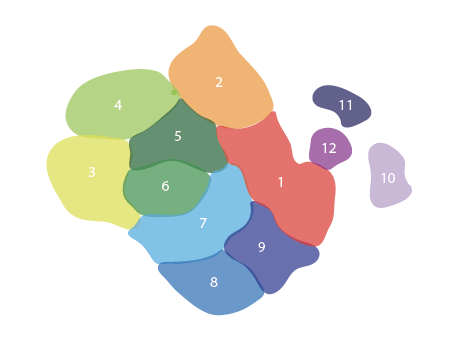

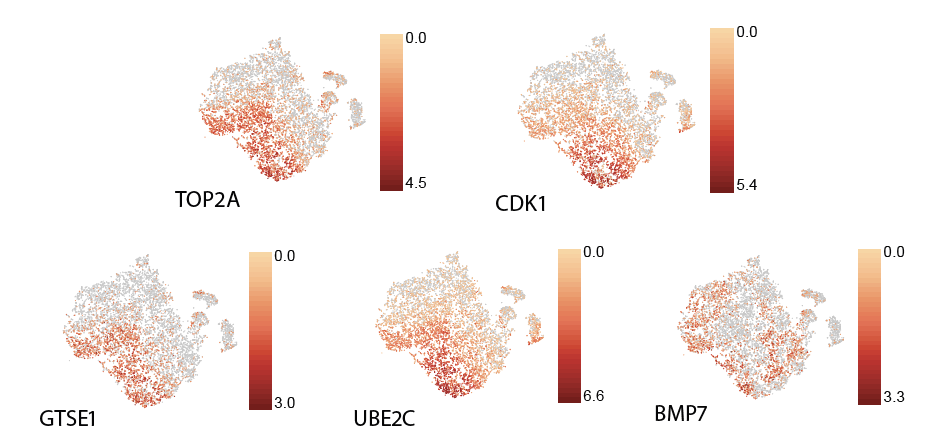

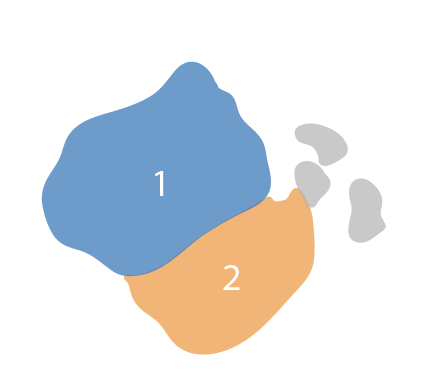

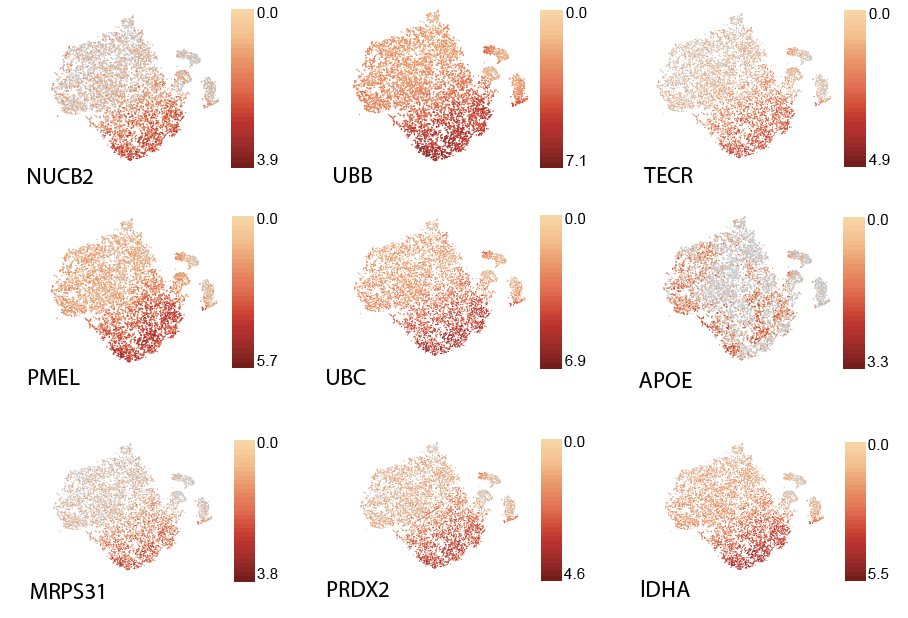


Supplemental Figure S5

B.

A.


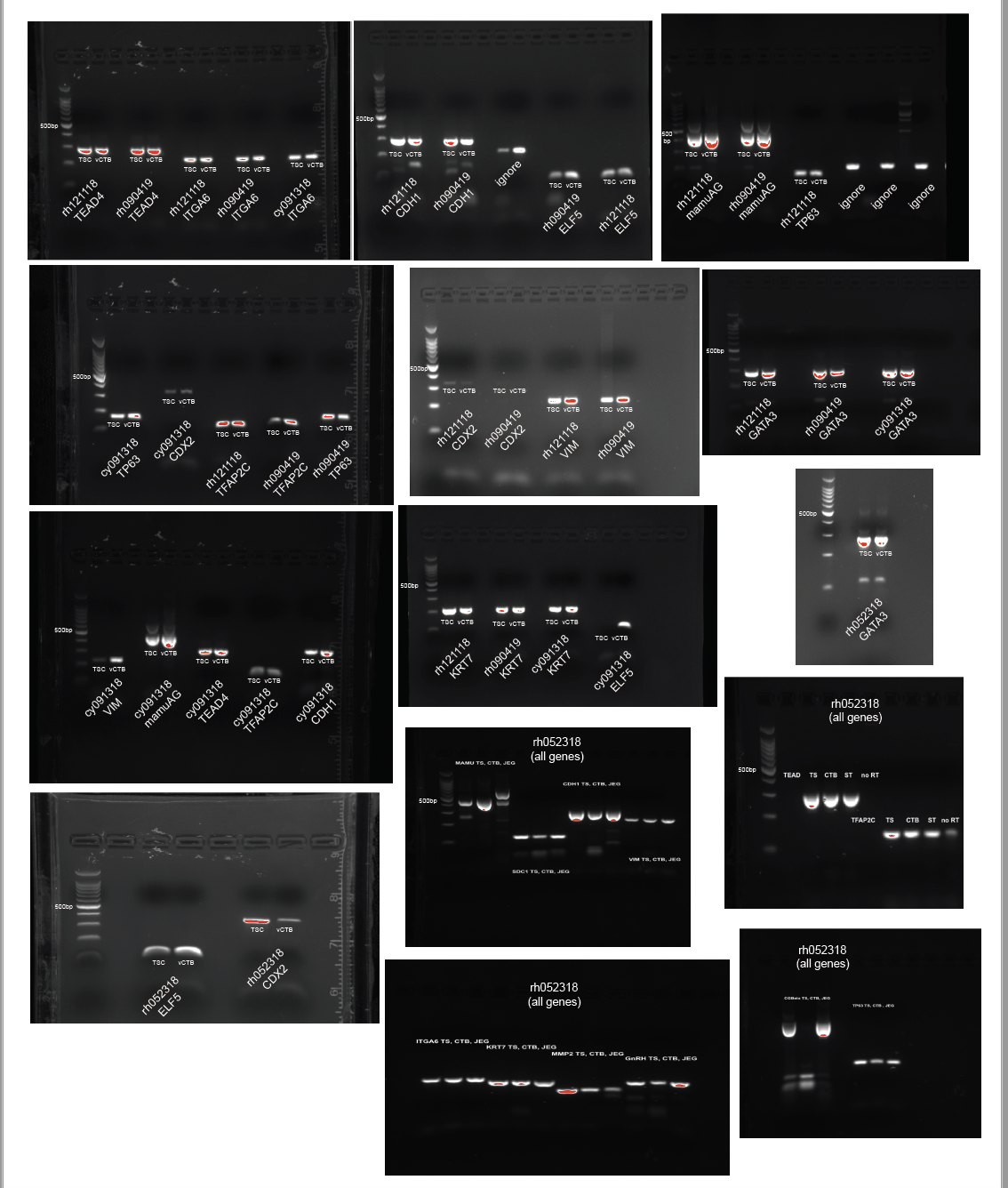


Supplemental Figure S6

**Supplemental Table S1.** Karyotype analysis of TSC lines

| TSC Line | Gestation  Date | Gender | Passage | Result | Summary |
| --- | --- | --- | --- | --- | --- |
| *rhesus* |  |  |  |  |  |
| rh052318 | 40 | XX | 35 | abnormal | 2 clones with translocations and inversion of chr 2, 3, 12. |
|  |  |  | 10a | normal | 17 normal cells, 2 non-clonal aberrations |
|  |  |  | 30a | abnormal | 10 normal cells, 4 cells trisomy chr 3, 6 cells non-clonal aberrations |
| rh121118 | 62 | XY | 13 | abnormal | 11 normal cells, 1 cell non-clonal aberrations, 3 abnormal clones: clone 1) 3 cells duplication chr 2, clone 2) 3 cells trisomy chr3, clone 3) 2 cells trisomy chr 3 |
| rh010319 | 58 | XY | 12 | normal | 18 normal cells, 2 cell non-clonal aberrations |
| rh012919 | 57 | XY | 7 | normal | 17 normal cells, 3 cells non-clonal aberrations |
| rh020119 | 74 | XX | 7 | normal | 17 normal cells, 3 non-clonal aberrations |
| rh090419 | 75 | XX | 8 | normal | 18 normal cells, 2 cell non-clonal aberrations |
| *cynomolgus* |  |  |  |  |  |
| cy091318 | 50 | XY | 7 | abnormal | 11 normal cells, 9 non-clonal aberrations. 33% of metaphase cells were polyploidy |
|  |  |  | 6a | abnormal | 3 cells analyzed, all were tetraploid |
| cy020519 | 57 | XX | 11 | abnormal | 11 normal cells, 5 cells trisomy chr 6, 4 cells non-clonal aberrations |

a: denotes a passage generated from a previous cryopreserved vial of cells; chr: chromosome

**Supplemental Table S2.** RNA-seq survey of gene expression in rh121118 cells. Gene expression values are represented as transcripts per million for one replicate of each cell type.

| **Gene Name** | **pri-CTB** | **TSC** | **pri-ST** | **ST-2D** | **ST-3D** | **Fibroblast** |
| --- | --- | --- | --- | --- | --- | --- |
| KRT7 | 64.78 | 96.88 | 98.26 | 67.61 | 325.36 | 424.47 |
| KRT8 | 917.26 | 3347.73 | 2610.18 | 2300.72 | 4488.45 | 667.67 |
| KRT18 | 1069.15 | 4627.46 | 4898.16 | 2728.91 | 6067.56 | 1042.26 |
| VIM | 313.73 | 2 | 377.77 | 0.49 | 0.27 | 2034.67 |
| ELF5 | 6.44 | 0.09 | 0 | 0.65 | 2.48 | 0 |
| GATA2 | 49.1 | 52.69 | 9.86 | 244.39 | 193.52 | 10.94 |
| GATA3 | 13.72 | 30.47 | 10.62 | 28.06 | 24.54 | 3.49 |
| GATA4 | 0.2 | 0.01 | 0.03 | 0.1 | 0.07 | 0 |
| GATA6 | 0.75 | 17.29 | 0.43 | 2.97 | 3.24 | 23.2 |
| GCM1 | 560.54 | 2 | 579.13 | 307.66 | 278.22 | 0 |
| HAND1 | 0.1 | 5.22 | 0 | 19.45 | 4.37 | 12.14 |
| ID2 | 52.64 | 23.68 | 21.15 | 17.95 | 3.76 | 29.88 |
| TEAD4 | 15.79 | 64.04 | 2.78 | 19.93 | 10.35 | 7.98 |
| TFAP2A | 65.77 | 36.41 | 47.59 | 123.35 | 73.36 | 3.75 |
| TFAP2C | 43.79 | 96.05 | 93.05 | 105.12 | 132.61 | 0.37 |
| TP63 | 27.48 | 18.9 | 0.58 | 14.82 | 5.93 | 0 |
| PLAC1 | 28.29 | 14.34 | 54.85 | 18.72 | 40.13 | 0.54 |
| PLAC8 | 1308 | 64.76 | 2948.95 | 739.73 | 1759.73 | 1355.35 |
| ERVFRD1 | 235.24 | 2.09 | 10.14 | 95.73 | 117.51 | 0.02 |
| ERVV1 | 324.6 | 0.64 | 81.3 | 95.4 | 110.26 | 0 |
| CDH1 | 47.96 | 180.59 | 66.65 | 126.72 | 60.03 | 0 |
| SDC1 | 25.18 | 12.25 | 50.2 | 131.52 | 143.01 | 12.34 |
| ITGA1 | 215.9 | 16.71 | 456.42 | 291.16 | 263.43 | 117.56 |
| ITGA5 | 356.7 | 18.99 | 1076.73 | 467.91 | 542.53 | 218.67 |
| ITGA6 | 325.51 | 302.33 | 153.79 | 328.09 | 180.13 | 91.96 |
| MMP2 | 686.77 | 611.56 | 1831.27 | 4279.43 | 6015.36 | 351.72 |
| MMP9 | 2.14 | 0.09 | 41.93 | 0.08 | 1.38 | 0 |
| CGA | 1227.43 | 334.23 | 585.2 | 25852.41 | 50906.56 | 0.25 |
| CGB8 | 0 | 0.38 | 0 | 911.61 | 1628.96 | 0 |
| CSH1 | 706.85 | 0.08 | 91.66 | 178.29 | 38.23 | 0.58 |
| CSH4 | 146.58 | 0 | 82.01 | 24.66 | 38.83 | 1.17 |
| CYP11A1 | 57.64 | 171.47 | 245.54 | 451.19 | 666.96 | 0 |
| CYP19A1 | 9.16 | 0.5 | 7.49 | 0.7 | 0.88 | 0 |
| ESRRB | 0 | 0 | 0 | 0.11 | 0 | 0 |
| GNRH1 | 2.13 | 1.36 | 3.87 | 1.24 | 2.08 | 0.38 |
| HSD17B1 | 0.21 | 9.02 | 5.23 | 11.56 | 11.01 | 0 |
| MAMUA | 327.58 | 261.09 | 1115.74 | 631.94 | 632.46 | 49.63 |
| MAMUE | 99.55 | 199.96 | 299.25 | 224 | 312.51 | 81.31 |
| MAMUAG | 1061.53 | 342.37 | 3825.23 | 2181.64 | 2397.55 | 9.65 |

**Supplemental Table S3.** RNA-seq survey of gene expression in rh121118 TSCs and EVTs. Gene expression values are represented as transcripts per million for one replicate of each cell type.

| **Gene Name** | **TSC** | **EVT** |
| --- | --- | --- |
| KRT7 | 96.88 | 28.63 |
| KRT8 | 3347.73 | 1697.04 |
| KRT14 | 20.22 | 161.35 |
| KRT18 | 4627.46 | 1514.6 |
| VIM | 2 | 0.12 |
| ELF5 | 0.09 | 0 |
| GATA2 | 52.69 | 149.77 |
| GATA3 | 30.47 | 33.13 |
| GATA4 | 0.01 | 0 |
| GATA6 | 17.29 | 3.67 |
| GCM1 | 2 | 127.45 |
| HAND1 | 5.22 | 5.18 |
| ID2 | 23.68 | 22.87 |
| TEAD4 | 64.04 | 25.76 |
| TFAP2A | 36.41 | 89.45 |
| TFAP2C | 96.05 | 81.85 |
| TP63 | 18.9 | 33.32 |
| PLAC1 | 14.34 | 7.16 |
| PLAC8 | 64.76 | 79.76 |
| ERVFRD1 | 2.09 | 21.66 |
| ERVV1 | 0.64 | 23.46 |
| CALD1 | 109.09 | 289.36 |
| CDH1 | 180.59 | 177.08 |
| CDH5 | 0.17 | 67.94 |
| SDC1 | 12.25 | 54.1 |
| ITGA1 | 16.71 | 78.49 |
| ITGA5 | 18.99 | 146.42 |
| ITGA6 | 302.33 | 479.35 |
| IL1RN | 610 | 944 |
| HTRA4 | 0.21 | 0.57 |
| MMP2 | 611.56 | 1688.1 |
| MMP9 | 0.09 | 0.02 |
| NCAM1 | 249.57 | 169.75 |
| BMP1 | 6.38 | 56.72 |
| TGFB1 | 22.59 | 49.55 |
| NOTCH1 | 2.44 | 2.21 |
| NOTCH2 | 33.42 | 67.45 |
| TCF4 | 3.67 | 2.57 |
| CGA | 334.23 | 4247.01 |
| CGB8 | 0.38 | 110.45 |
| CSH1 | 0.08 | 37.84 |
| CSH4 | 0 | 9.15 |
| MAMUA | 261.09 | 445.96 |
| MAMUE | 199.96 | 119.33 |
| MAMUAG | 342.37 | 1694.32 |

**Supplemental Table S4.** miRNA expression of miRNAs within the pregnancy-associated miRNA cluster. miRNA expression levels represented as transcripts per million

|  | **pri-CTB** | **TSC** | **pri-ST** | **ST-2D** | **ST-3D** | **EVT** | **fibroblast** |
| --- | --- | --- | --- | --- | --- | --- | --- |
| **miR-371-3 cluster** | | | | | | | |
| miR-371-3p | 0 | 1 | 0 | 1 | 0 | 1 | 0 |
| miR-371-5p | 11 | 884 | 23 | 124 | 56 | 9 | 0 |
| miR-372-3p | 6 | 207 | 20 | 52 | 25 | 3 | 0 |
| miR-372-5p | 0 | 3 | 0 | 0 | 0 | 0 | 0 |
| miR-373 | 6 | 147 | 6 | 25 | 18 | 1 | 0 |
| **C19MC** | | | | | | | |
| miR-512-3p | 323 | 134 | 463 | 200 | 476 | 64 | 0 |
| miR-512-5p | 2706 | 3064 | 3329 | 1901 | 9167 | 1473 | 0 |
| miR-1323-3p | 0 | 0 | 3 | 0 | 2 | 0 | 0 |
| miR-1323-5p | 7263 | 9535 | 17798 | 10796 | 18964 | 7026 | 5 |
| miR-498-5p | 2548 | 2008 | 3321 | 1960 | 5295 | 1016 | 0 |
| miR-519e | 23361 | 23081 | 38525 | 24422 | 51396 | 8382 | 0 |
| miR-1283 | 583 | 320 | 492 | 338 | 352 | 80 | 0 |
| miR-520a-3p | 1455 | 1209 | 1882 | 1783 | 3497 | 465 | 0 |
| miR-520a-5p | 1863 | 939 | 1974 | 2228 | 1294 | 446 | 0 |
| miR-526b | 5524 | 2766 | 9295 | 3723 | 5678 | 1284 | 0 |
| miR-525 | 392 | 141 | 436 | 363 | 349 | 81 | 0 |
| miR-523a | 492 | 230 | 601 | 316 | 500 | 94 | 0 |
| miR-518f | 10919 | 11651 | 15307 | 13635 | 15787 | 4859 | 0 |
| miR-519a-3p | 46 | 47 | 8 | 69 | 58 | 3 | 0 |
| miR-519a-5p | 484 | 867 | 805 | 781 | 1355 | 457 | 0 |
| miR-518b | 7215 | 4904 | 8910 | 4982 | 8068 | 1942 | 1 |
| miR-518c-5p | 43 | 41 | 90 | 40 | 66 | 17 | 0 |
| miR-518c-3p | 4095 | 1256 | 3532 | 2119 | 1785 | 318 | 0 |
| miR-524-5p | 668 | 433 | 780 | 783 | 1414 | 160 | 0 |
| miR-524-3p | 28 | 24 | 37 | 32 | 38 | 1 | 0 |
| miR-517a | 2598 | 1148 | 2043 | 1701 | 2054 | 276 | 0 |
| miR-519d | 1036 | 472 | 997 | 751 | 782 | 130 | 0 |
| miR-518a-3p | 1632 | 1642 | 3748 | 2207 | 6296 | 523 | 0 |
| miR-518a-5p | 437 | 361 | 398 | 369 | 669 | 64 | 0 |
| miR-520g-3p | 8 | 1 | 5 | 5 | 4 | 0 | 0 |
| miR-518d-3p | 552 | 573 | 757 | 795 | 1035 | 131 | 0 |
| miR-518d-5p | 6598 | 7371 | 17889 | 8850 | 17281 | 4809 | 1 |
| miR-523b | 381 | 289 | 364 | 421 | 454 | 87 | 0 |
| miR-516b | 2116 | 1309 | 2990 | 2335 | 2943 | 380 | 0 |
| miR-518a-3p | 1632 | 1642 | 3748 | 2207 | 6296 | 523 | 0 |
| miR-518a-5p | 437 | 361 | 398 | 369 | 669 | 64 | 0 |
| miR-517c | 4474 | 5917 | 8006 | 7528 | 11482 | 2061 | 0 |
| miR-519b | 76 | 62 | 154 | 86 | 182 | 35 | 0 |
| miR-521 | 11 | 8 | 16 | 6 | 27 | 4 | 0 |
| miR-518e | 39 | 50 | 110 | 97 | 213 | 32 | 0 |
| miR-518a-3p | 1632 | 1642 | 3748 | 2207 | 6296 | 523 | 0 |
| miR-518a-5p | 437 | 361 | 398 | 369 | 669 | 64 | 0 |
| miR-516a-3p | 1085 | 1371 | 2165 | 1906 | 2489 | 531 | 0 |
| miR-516a-5p | 21483 | 10598 | 43883 | 15764 | 21448 | 4654 | 1 |
| **C14MC** | | | | | | | |
| miR-770-5p | 0 | 0 | 0 | 0 | 0 | 0 | 1 |
| miR-493-3p | 12 | 0 | 0 | 0 | 0 | 0 | 17 |
| miR-493-5p | 108 | 0 | 31 | 0 | 0 | 0 | 293 |
| miR-337-3p | 8 | 0 | 3 | 0 | 0 | 0 | 26 |
| miR-337-5p | 10 | 0 | 0 | 0 | 0 | 0 | 10 |
| miR-665 | 1 | 0 | 0 | 0 | 0 | 0 | 3 |
| miR-431 | 529 | 0 | 69 | 0 | 1 | 0 | 253 |
| miR-433-3p | 6 | 0 | 2 | 0 | 0 | 0 | 25 |
| miR-433-5p | 1 | 0 | 0 | 0 | 0 | 0 | 0 |
| miR-127-3p | 1571 | 0 | 203 | 0 | 0 | 0 | 2193 |
| miR-127-5p | 4 | 0 | 1 | 0 | 0 | 0 | 0 |
| miR-432-3p | 0 | 0 | 0 | 0 | 0 | 0 | 2 |
| miR-432-5p | 775 | 0 | 167 | 0 | 0 | 1 | 3776 |
| miR-136 | 5 | 0 | 0 | 0 | 0 | 0 | 0 |
| miR-370-3p | 65 | 0 | 3 | 0 | 0 | 0 | 173 |
| miR-370-5p | 3 | 0 | 1 | 0 | 0 | 0 | 16 |
| miR-379-3p | 5 | 0 | 0 | 0 | 0 | 0 | 18 |
| miR-379-5p | 217 | 0 | 22 | 0 | 0 | 0 | 309 |
| miR-411-3p | 2 | 0 | 0 | 0 | 0 | 0 | 11 |
| miR-411-5p | 363 | 0 | 19 | 0 | 0 | 0 | 280 |
| miR-299-3p | 88 | 0 | 7 | 0 | 0 | 0 | 50 |
| miR-299-5p | 427 | 0 | 82 | 0 | 2 | 3 | 992 |
| miR-380-3p | 0 | 0 | 0 | 0 | 0 | 0 | 4 |
| miR-380-5p | 0 | 0 | 0 | 0 | 0 | 0 | 2 |
| miR-323a-3p | 35 | 0 | 4 | 0 | 0 | 0 | 145 |
| miR-323a-5p | 0 | 0 | 0 | 0 | 0 | 0 | 0 |
| miR-758-3p | 2 | 0 | 0 | 0 | 0 | 0 | 5 |
| miR-758-5p | 1 | 0 | 0 | 0 | 0 | 0 | 0 |
| miR-329-3p | 13 | 0 | 1 | 0 | 0 | 0 | 56 |
| miR-329-1-5p | 0 | 0 | 0 | 0 | 0 | 0 | 2 |
| miR-329-2-5p | 0 | 0 | 0 | 0 | 0 | 0 | 0 |
| miR-494-3p | 15 | 0 | 4 | 0 | 0 | 0 | 50 |
| miR-494-5p | 0 | 0 | 0 | 0 | 0 | 0 | 1 |
| miR-543-3p | 13 | 0 | 2 | 0 | 0 | 0 | 38 |
| miR-543-5p | 4 | 0 | 0 | 0 | 0 | 0 | 13 |
| miR-495-3p | 10 | 0 | 0 | 0 | 0 | 0 | 11 |
| miR-495-5p | 5 | 0 | 1 | 0 | 0 | 0 | 9 |
| miR-376c-3p | 121 | 0 | 7 | 0 | 0 | 0 | 52 |
| miR-376c-5p | 6 | 0 | 0 | 0 | 0 | 0 | 1 |
| miR-376a-3p | 60 | 0 | 6 | 1 | 0 | 0 | 46 |
| miR-376a-1-5p | 3 | 0 | 1 | 0 | 0 | 0 | 0 |
| miR-376a-2-5p | 1 | 0 | 0 | 0 | 0 | 0 | 0 |
| miR-654-3p | 386 | 0 | 57 | 0 | 0 | 2 | 1631 |
| miR-654-5p | 0 | 0 | 0 | 0 | 0 | 0 | 0 |
| miR-376b-3p | 6 | 0 | 2 | 0 | 0 | 0 | 2 |
| miR-376b-5p | 3 | 0 | 0 | 0 | 0 | 0 | 1 |
| miR-376a-1-5p | 3 | 0 | 1 | 0 | 0 | 0 | 0 |
| miR-376a-2-5p | 1 | 0 | 0 | 0 | 0 | 0 | 0 |
| miR-376a-3p | 60 | 0 | 6 | 1 | 0 | 0 | 46 |
| miR-1185-3p | 3 | 0 | 1 | 0 | 0 | 0 | 10 |
| miR-1185-5p | 8 | 0 | 0 | 0 | 0 | 0 | 2 |
| miR-381-3p | 77 | 0 | 9 | 0 | 0 | 1 | 88 |
| miR-381-5p | 0 | 0 | 0 | 0 | 0 | 0 | 1 |
| miR-487b-3p | 110 | 0 | 18 | 0 | 0 | 0 | 183 |
| miR-487b-5p | 4 | 0 | 0 | 0 | 0 | 0 | 30 |
| miR-539 | 0 | 0 | 0 | 0 | 0 | 0 | 0 |
| miR-889-3p | 53 | 0 | 4 | 0 | 0 | 0 | 44 |
| miR-889-5p | 0 | 0 | 0 | 0 | 0 | 0 | 0 |
| miR-544 | 0 | 0 | 0 | 0 | 0 | 0 | 0 |
| miR-487a | 0 | 0 | 0 | 0 | 0 | 0 | 0 |
| miR-382-3p | 27 | 0 | 5 | 0 | 0 | 0 | 69 |
| miR-382-5p | 371 | 0 | 76 | 0 | 0 | 0 | 1164 |
| miR-134-3p | 0 | 0 | 0 | 0 | 0 | 0 | 1 |
| miR-134-5p | 95 | 0 | 12 | 0 | 0 | 2 | 544 |
| miR-668 | 4 | 0 | 1 | 0 | 0 | 0 | 9 |
| miR-485-3p | 46 | 0 | 14 | 0 | 1 | 0 | 1102 |
| miR-485-5p | 32 | 0 | 2 | 0 | 0 | 0 | 201 |
| miR-323b-3p | 28 | 0 | 6 | 0 | 0 | 0 | 279 |
| miR-323b-5p | 0 | 0 | 0 | 0 | 0 | 0 | 0 |
| miR-154-3p | 2 | 0 | 0 | 0 | 0 | 0 | 6 |
| miR-154-5p | 48 | 0 | 8 | 0 | 0 | 0 | 54 |
| miR-496 | 4 | 0 | 0 | 0 | 0 | 0 | 7 |
| miR-337-3p | 8 | 0 | 3 | 0 | 0 | 0 | 26 |
| miR-337-5p | 10 | 0 | 0 | 0 | 0 | 0 | 10 |
| miR-541-3p | 0 | 0 | 0 | 0 | 0 | 0 | 0 |
| miR-541-5p | 0 | 0 | 0 | 0 | 0 | 0 | 10 |
| miR-409-3p | 532 | 0 | 73 | 0 | 0 | 1 | 2165 |
| miR-409-5p | 34 | 0 | 5 | 0 | 0 | 0 | 115 |
| miR-412-3p | 0 | 0 | 0 | 0 | 0 | 0 | 1 |
| miR-412-5p | 33 | 0 | 5 | 0 | 0 | 0 | 16 |
| miR-369-3p | 78 | 0 | 1 | 0 | 0 | 0 | 45 |
| miR-369-5p | 166 | 0 | 15 | 0 | 0 | 0 | 335 |
| miR-410-3p | 2 | 0 | 0 | 0 | 0 | 0 | 3 |
| mIR-410-5p | 0 | 0 | 0 | 0 | 0 | 0 | 13 |
| miR-656-3p | 3 | 0 | 0 | 0 | 0 | 0 | 11 |
| miR-656-5p | 0 | 0 | 0 | 0 | 0 | 0 | 1 |
| miR-1247-3p | 7 | 0 | 0 | 0 | 0 | 0 | 0 |
| miR-1247-5p | 118 | 0 | 14 | 0 | 0 | 0 | 0 |

**Supplemental Table S5.** Summary of the mean percentage of cells expressing a marker by flow cytometry.

|  | Cytokeratin 7/8  mean % pos ± SD | Vimentin  mean % neg± SD | Ki67  Mean % pos ± SD | Mamu-AG  mean % pos ± SD | CD56  mean % pos± SD |
| --- | --- | --- | --- | --- | --- |
| TSC experiment | | | | | |
| TSC | 72.3 ± 6.76 | 99.28 ± 1.08 | 86.97 ± 0.24 | 84.6 ± 4.76 | 87.39 ± 0.17 |
| unstained TSC* | 0.00 | 100.00 | 0.00 | 0.00 | 0.00 |
| fibroblast* | 80.80 | 1.13 | 93.84 | 44.50 | 97.39 |
| EVT experiment | | | | | |
| EVT | 94.76 ± 2.74 | 99.37 ± 0.49 | 94.26 ± 3.86 | 95.05 ± 2.13 | 95.90 ± 3.26 |
| TSC* | 98.53 | 100.00 | 0.10 | 0.10 | 0.20 |
| unstained TSC* | 0.10 | 97.90 | 98.59 | 91.63 | 98.53 |
| Fibroblast* | 91.05 | 0.22 | 92.32 | 22.50 | 98.16 |

An individual flow cytometry experiment was performed for TSC replicates and EVT replicates due to differences in cell size. In the EVT experiment, one TSC and fibroblast replicate was run in parallel. *: denotes a line that was evaluated in the same run with 1 replicate per cell type.

Pos: positive, neg: negative.

**Supplemental Table S6.** Differentially expressed genes between passage 10 TSC clusters identified by scRNA-Seq t-SNE analysis. Parenthesis indicate number of genes either significantly down or upregulated (p <0.05) and greater than 2-fold change in expression.

| **Cluster 1** | **Cluster 2** | **Cluster 4** | **Cluster 5** | **Cluster 6** | **Cluster 7** | **Cluster 9** |
| --- | --- | --- | --- | --- | --- | --- |
| **Up (12)** | **Down (7)** | **Down (1)** | **Down (9)** | **Down (1)** | **Up (6)** | **Down (2)** |
| TOP2A | TOP2A | TOP2A | TOP2A | TOP2A | TOP2A | EDA2R |
| PRC1 | PRC1 |  | PRC1 |  | UBE2S | IRS2 |
| CHAC1 | H2AFX |  | CHAC1 |  | PRC1 |  |
| PRRG4 | UBE2S |  | BIRC5 |  | BIRC5 |  |
| EDA2R | BIRC5 |  | WEE1 |  | ARL6IP1 |  |
| RHNO1 | CKAP5 |  | UBE2S |  | CKAP5 |  |
| SUN2 | ARL6IP1 |  | IRS2 |  |  |  |
| UBE2S |  |  | SLC7A1 |  | **Down (2)** |  |
| MYADM |  |  | H2AFX |  | IRS2 |  |
| WEE1 |  |  |  |  | THBS1 |  |
| H2AFX |  |  |  |  |  |  |
| BIRC5 |  |  |  |  |  |  |

**Supplemental Table S7.** Differentially expressed genes between TSC clusters identified by scRNA-Seq K-means analysis of p2 and p10 cells combined. Parenthesis indicate number of genes either significantly down or upregulated (p <0.05) and greater than 2-fold change in expression.

| **Cluster 1** | **Cluster 2** |
| --- | --- |
| **Down (187)** | **Up (171)** |
| APOE | NUCB2 |
| NUCB2 | UBB |
| UBB | TECR |
| TECR | PMEL |
| PMEL | UBC |
| PLTP | APOE |
| UBC | MRPS31 |
| TUBA3C | PRDX4 |
| PRDX4 | PLTP |
| MRPS31 | LDHA |
| LDHA | TUBA3C |
| PRDX2 | PSMB3 |
| PSMB3 | ANXA2 |
| CTNNBL1 | PRDX2 |
| ANXA2 | CTNNBL1 |
| NSDHL | CDK1 |
| CDK1 | NSDHL |
| CKB | EIF4A3 |
| DRG1 | CKB |
| EIF4A3 | DRG1 |
| BLVRB | PSME2 |
| RBM42 | TYMS |
| PSME2 | PGK1 |
| SUGT1 | WDR61 |
| ISYNA1 | SUGT1 |
| PGK1 | BLVRB |
| TYMS | PSMC4 |
| PSMC4 | DDX39A |
| DDX39A | RBM42 |
| WDR61 | CD9 |
| KPNA2 | KPNA2 |
| CD9 | PSMD12 |
| PSMD12 | ISYNA1 |
| FOLR1 | PNP |
| C22orf28 | LDHB |
| LDHB | GHITM |
| PNP | ATP5B |
| GHITM | C22orf28 |
| ATP5B | FOLR1 |
| RUVBL2 | RUVBL2 |
| KRT18 | UFD1L |
| UFD1L | PSMA6 |
| PSMC5 | CLDN6 |
| CLDN6 | KRT18 |
| PDIA3 | TK1 |
| FAM50A | PDIA3 |
| PSMA6 | PRMT1 |
| PSMD7 | PSMC5 |
| PRMT1 | PSMD7 |
| PKM | NUDT5 |
| ACP5 | RAB11A |
| TK1 | UQCRC2 |
| PLD3 | PKM |
| RAB11A | FAM50A |
| NUDT5 | TUBA1B |
| UQCRC2 | KRT7 |
| PRPF19 | ITM2B |
| KRT7 | ACP5 |
| ITM2B | PRPF19 |
| AURKB | RDM1 |
| TUBA1B | MYL12B |
| RDM1 | AURKB |
| CTSA | AHSA1 |
| PSMC3 | LGALS3 |
| MYL12B | PSMC1 |
| AHSA1 | UBE2C |
| MVD | MVD |
| PSMC1 | PYGL |
| GRN | PSMA3 |
| UBE2C | NDUFA9 |
| LGALS3 | PA2G4 |
| XRCC6 | PLK1 |
| PYGL | PSMC3 |
| PA2G4 | MYL12A |
| WDR18 | XRCC6 |
| PLK1 | WDR18 |
| TMEM205 | CTSA |
| PSMA3 | GOT1 |
| NDUFA9 | PLD3 |
| HMOX1 | EML2 |
| GOT1 | CDC123 |
| MYL12A | RNASEH2A |
| EML2 | HMOX1 |
| PPP2R1A | TMEM205 |
| RNASEH2A | ATP5C1 |
| PPIB | PPP2R1A |
| NDUFV1 | SNRPB2 |
| COMMD4 | GRN |
| CDC123 | NDUFV1 |
| ATP6V0D1 | COMMD4 |
| CLDN7 | TPI1 |
| SNRPB2 | MAGEA4 |
| HSP90B1 | PPIB |
| PVRL2 | ATP6V0D1 |
| IRS2 | CLDN7 |
| TPI1 | MCTS1 |
| ADRM1 | TUBG1 |
| ATP5C1 | EIF2S1 |
| MCTS1 | MRPL46 |
| HEXA | ADRM1 |
| TUBG1 | HSP90B1 |
| MAGEA4 | MPV17L2 |
| EIF2S1 | RSU1 |
| GPS1 | ACAT1 |
| SLC16A3 | ATP5H |
| CYBA | SAR1A |
| ETFB | ORMDL2 |
| CD151 | CD151 |
| NOSIP | AP1M2 |
| MPV17L2 | GPS1 |
| RSU1 | SLC16A3 |
| MRPL46 | MYL6 |
| CDC37 | NSMCE1 |
| ORMDL2 | TALDO1 |
| ACAT1 | ESD |
| ATP5H | KRR1 |
| SAR1A | CDC37 |
| NSMCE1 | POLR2C |
| AP1M2 | HEXA |
| PHB | ANP32A |
| MYL6 | PSMB6 |
| HSD17B14 | PHB |
| PSMD3 | NOSIP |
| SF3B2 | PVRL2 |
| ANP32A | SF3B2 |
| GSTP1 | PSME1 |
| TALDO1 | GSTP1 |
| PSMB6 | HSD17B14 |
| POLR2C | ETFB |
| TIMM50 | EXOSC8 |
| ESD | TIMM50 |
| PAGE4 | TMBIM6 |
| TAC3 | NLRP2 |
| KRR1 | DPM1 |
| NLRP2 | ARPC3 |
| TMBIM6 | NDUFS3 |
| BCAP31 | BCAP31 |
| NDUFS3 | MTCH2 |
| FUS | CYBA |
| PELP1 | PSMD3 |
| NEMF | FUS |
| PSME1 | VGLL1 |
| PRSS8 | PAGE2B |
| PAGE2B | MAGOHB |
| EXOSC8 | PCNA |
| VGLL1 | RSL1D1 |
| DPM1 | TRAPPC4 |
| MFGE8 | TSG101 |
| PCNA | MRPL48 |
| ARPC3 | TAC3 |
| MVB12A | HSD17B10 |
| RSL1D1 | PAGE4 |
| VPS25 | NEMF |
| TSG101 | VPS25 |
| TUFM | SPCS2 |
| MTCH2 | HNRNPH3 |
| RBM25 | TXNL1 |
| MRPL48 | CHMP4A |
| HNRNPH3 | GLRX3 |
| HSD17B10 | PELP1 |
| SPCS2 | MVB12A |
| MAGOHB | PRSS8 |
| CHMP4A | RAN |
| TRAPPC4 | TUFM |
| BSG | STIP1 |
| TXNL1 | COMMD3 |
| ILK | ACAA2 |
| AMDHD2 | EIF4A1 |
| GLRX3 | DERA |
| TOMM40 | MFGE8 |
| STIP1 | RBM25 |
| ATP6AP1 |  |
| MRPL38 | **Down (1)** |
| RAN | IRS2 |
| TUBA1C |  |
| POLR2E |  |
| MTHFD1 |  |
| TIMM23 |  |
| DERA |  |
| DAD1 |  |
| EIF4A1 |  |
| SNRNP70 |  |
| ACAA2 |  |
| COMMD3 |  |
| STX8 |  |
| AKAP8L |  |
| JUNB |  |

**Supplemental Table S8.** Differentially expressed genes between TSC clusters segregated by the LMO algorithm applied to scRNA-seq gene expression levels of combined p2 and p10 cells. Parenthesis indicate number of genes either significantly down or upregulated (p <0.05) and greater than 2-fold change in expression.

| **Cluster 1** | **Cluster 2** | **Cluster 3** | **Cluster 4** | **Cluster 5** | **Cluster 6** | **Cluster 8** | **Cluster 9** |
| --- | --- | --- | --- | --- | --- | --- | --- |
|  |  |  |  |  |  |  |  |
| **Up (3)** | **Down (75)** | **Up (13)** | **Up (1)** | **Up (2)** | **Down (8)** | **Up (95)** | **Up (51)** |
| APOE | CDK1 | WEE1 | PLEKHF1 | TOP2A | PLK1 | CCNB2 | MCM5 |
| PMEL | UBE2C | THBS1 |  | GTSE1 | UBE2C | PLK1 | TK1 |
| FOLR1 | TOP2A | BMP7 | **Down (24)** |  | APOE | CDCA3 | RAD51 |
|  | PRC1 | FADS2 | CDK1 | **Down (28)** | CCNB2 | UBE2C | PCNA |
| **Down (19)** | GTSE1 | MT1E | UBE2C | PMEL | UBB | KPNA2 | FEN1 |
| TOP2A | CDCA3 | MYADM | TOP2A | TAC3 | PMEL | AURKB | PRIM1 |
| GTSE1 | TK1 | EDA2R | AURKB | APOE | CDCA3 | CDK1 | GOT1 |
| UBE2C | KPNA2 | CGB | CDCA3 | PLTP | UBC | TUBA3C | ORC6 |
| PRC1 | PLK1 |  | PRC1 | ACP5 |  | DLGAP5 | LDHA |
| CDK1 | RAD51 | **Down (40)** | TK1 | PAGE2 |  | NUP37 | NUCB2 |
| CDCA3 | AURKB | APOE | GTSE1 | CD9 |  | UBB | MPV17L2 |
| AURKB | UBB | TECR | PLK1 | PSMB3 |  | TUBA1B | PA2G4 |
| PLK1 | TPX2 | PSMB3 | KPNA2 | PRDX4 |  | DDX39A | NSDHL |
| TPX2 | KIF20B | PSME1 | TUBA3C | NUCB2 |  | KIF20B | MVD |
| BIRC5 | MCM5 | LDHB | KIF20B | PRDX2 |  | RBM42 | KRT7 |
| CASC5 | CTNNBL1 | BLVRB | RAD51 | BMP7 |  | MRPS31 | TECR |
| UBE2S | APOE | PRDX2 | NUP37 | CLDN6 |  | TYMS | PLTP |
| KIF20B | NUCB2 | PRDX4 | MCM5 | UBB |  | ARL6IP1 | CTNNBL1 |
| H2AFX | PMEL | NUCB2 | TECR | ATP5B |  | PSMD12 | UBC |
| MCM10 | UBC | FOLR1 | APOE | PSME2 |  | RDM1 | CLDN6 |
| BRCA2 | GOT1 | ANXA2 | NUCB2 | BLVRB |  | UBC | PRDX4 |
| TK1 | CKB | MRPS31 | UBB | TECR |  | NUCB2 | KRT18 |
| CHAF1A | TUBA3C | UBB | UBC | MAGEH1 |  | PRC1 | LGALS3 |
| RHNO1 | NUP85 | SLC25A5 | PKMYT1 | ANXA2 |  | CTNNBL1 | CLDN7 |
|  | CLDN6 | PSME2 | ANXA2 | PNP |  | TUBA1C | PMEL |
|  | PCNA | PSMC4 | TPX2 | UBC |  | CDKN3 | APOE |
|  | BIRC5 | PSMA3 | EIF4A3 | MCM5 |  | UBE2S | PKM |
|  | DRG1 | TUBA3C |  | FOLR1 |  | C22orf28 | CD9 |
|  | LDHA | KRT19 |  | MRPS31 |  | DRG1 | CKB |
|  | TAC3 | PMEL |  | LDHA |  | TBL3 | PPAN |
|  | PLTP | SUGT1 |  | CKB |  | PSMD7 | PRDX2 |
|  | ORC6 | ESD |  | ISYNA1 |  | TOP2A | PGK1 |
|  | FEN1 | UBC |  |  |  | RUVBL2 | EIF4A3 |
|  | BMP7 | KRT18 |  |  |  | LDHA | MCM10 |
|  | PSMD7 | ISYNA1 |  |  |  | SUGT1 | LDHB |
|  | ATP5B | NSDHL |  |  |  | PSMB3 | GINS2 |
|  | PRIM1 | PSMC5 |  |  |  | RAD51 | CHAF1A |
|  | RANGAP1 | WDR61 |  |  |  | NUP85 | DRG1 |
|  | ARL6IP1 | RPN2 |  |  |  | AHSA1 | NUDT5 |
|  | NUP37 | RNASEH2A |  |  |  | CKB | PRPF19 |
|  | PKM | RBM42 |  |  |  | SAAL1 | MPHOSPH8 |
|  | UBE2S | ACAT1 |  |  |  | MARS | NUP85 |
|  | PSMD12 | EIF4A3 |  |  |  | TECR | BLVRB |
|  | ANXA2 | PLTP |  |  |  | CKLF | UBB |
|  | MRPS31 | KRT7 |  |  |  | NSDHL | PNP |
|  | PKMYT1 | TUFM |  |  |  | PRDX4 | FOLR1 |
|  | RUVBL2 | RUVBL2 |  |  |  | PSMC4 | ANXA2 |
|  | CENPJ | MCTS1 |  |  |  | RNASEH2A | RAB11A |
|  | DLGAP5 | NUP37 |  |  |  | BIRC5 | HSP90B1 |
|  | H2AFX | PSMA6 |  |  |  | PGK1 | PYGL |
|  | MCM10 |  |  |  |  | FAM50A | TUBA3C |
|  | CASC5 |  |  |  |  | TPX2 |  |
|  | GHITM |  |  |  |  | CARS | Down (1) |
|  | TECR |  |  |  |  | EIF4A3 | EDA2R |
|  | PRDX4 |  |  |  |  | TK1 |  |
|  | NSDHL |  |  |  |  | PSMA6 |  |
|  | ACP5 |  |  |  |  | KRR1 |  |
|  | LGMN |  |  |  |  | PSMC5 |  |
|  | NLRP2 |  |  |  |  | PLTP |  |
|  | EIF4A3 |  |  |  |  | PSMC1 |  |
|  | CD9 |  |  |  |  | CIAPIN1 |  |
|  | TYMS |  |  |  |  | PRDX2 |  |
|  | PSMC3 |  |  |  |  | PMEL |  |
|  | C22orf28 |  |  |  |  | MYL12A |  |
|  | PDIA3 |  |  |  |  | GTSE1 |  |
|  | TUBA1B |  |  |  |  | NUDT5 |  |
|  | CTSA |  |  |  |  | FUS |  |
|  | TBL3 |  |  |  |  | PRMT1 |  |
|  | DDX39A |  |  |  |  | GHITM |  |
|  | MPV17L2 |  |  |  |  | PSMD3 |  |
|  | SERTAD1 |  |  |  |  | EIF2S1 |  |
|  | PLD3 |  |  |  |  | POLR2C |  |
|  | PSMC4 |  |  |  |  | PSMC3 |  |
|  | FAM50A |  |  |  |  | XRCC6 |  |
|  | DHCR7 |  |  |  |  | UQCRC2 |  |
|  |  |  |  |  |  | WDR18 |  |
|  |  |  |  |  |  | SNRPB2 |  |
|  |  |  |  |  |  | HMGB1 |  |
|  |  |  |  |  |  | PSME2 |  |
|  |  |  |  |  |  | UFD1L |  |
|  |  |  |  |  |  | RAB11A |  |
|  |  |  |  |  |  | SARS2 |  |
|  |  |  |  |  |  | MAGOHB |  |
|  |  |  |  |  |  | STIP1 |  |
|  |  |  |  |  |  | PRPF19 |  |
|  |  |  |  |  |  | TUBG1 |  |
|  |  |  |  |  |  | APOE |  |
|  |  |  |  |  |  | SNRNP70 |  |
|  |  |  |  |  |  | LDHB |  |
|  |  |  |  |  |  | ANXA2 |  |
|  |  |  |  |  |  | GOT1 |  |
|  |  |  |  |  |  | PNP |  |
|  |  |  |  |  |  | WDR61 |  |
|  |  |  |  |  |  | RPN2 |  |
|  |  |  |  |  |  | FTHL17 |  |
|  |  |  |  |  |  |  |  |
|  |  |  |  |  |  | **Down (6)** |  |
|  |  |  |  |  |  | IRS2 |  |
|  |  |  |  |  |  | CRISPLD2 |  |
|  |  |  |  |  |  | DUSP5 |  |
|  |  |  |  |  |  | SLC7A8 |  |
|  |  |  |  |  |  | FOSB |  |
|  |  |  |  |  |  | MARVELD1 |  |

**Supplementary Table S9.** Immunocytochemistry antibodies and fluorescent conjugates

| Target | Vendor | Catalog  Number | Clone | Stock Concentration | Final Concentration | Lot# | Isotype | RRID # |
| --- | --- | --- | --- | --- | --- | --- | --- | --- |
| Primary antibodies | | | | | | | | |
| AP2- γ | Invitrogen | PA5-84329 | Poly | 0.050 mg/ml | 0.5 μg/ml |  | rabbit IgG | AB_2791481 |
| Cytokeratin | Sigma-Aldrich/Cell Marque | 452M-94 | CAM5.2 | 81.6 μg/ml | 1.6 μg/ml |  | mouse IgG2a |  |
| Ki-67-Alexa 647 | BD | BD561126 | B56 |  |  |  |  |  |
| Mamu-AG^1^ | Golos lab |  | 25D3 | 1.92 mg/ml | 5 μg/ml | A8082175 | IgG1 |  |
| Macaque CG^2^ | University of California-Davis |  | 518B7 | 2 mg/ml |  | 7/05 | IgG1 |  |
| Vimentin | BD | BD550513 |  | 0.5 mg/ml | 5 μg/ml | 8169851 | IgG1 |  |
| Secondary Antibodies | | | | | | | | |
| Donkey anti-mouse CF568 | Biotium | 20105 |  | 2 mg/ml | 2 μg/ml | 17C1116 | polyclonal |  |
| Donkey anti-rabbit CF640 | Biotium | 20178 |  | 2 mg/ml | 2 μg/ml | 17C0131 | polyclonal |  |
| Donkey anti-rabbit | Biotium | 20098 |  | 2 mg/ml | 2 μg/ml | 18C0703 | polyclonal |  |
| Fluorescent Conjugates | | |  |  |  |  |  |  |
| DAPI, diacetate | Invitrogen | D3571 |  | 10 mg/ml | 1 μg/ml |  |  |  |
| Hoerscht 33342 | BD Pharminogen | 561908 |  | 1 mg/ml | 2 μg/ml | 9037866 |  |  |
| Wheat germ agglutinin (WGA) | Invitrogen | W7024 |  | 1 mg/ml | 5 μg/ml | 2068234 |  |  |

^1^Slukvin et al. 2000 PNAS; ^2^Ziegler et al. 1993 Am J Primatol.

| Gene | Forward (5’-3’) | Reverse (5’-3’) | Amplicon Size |
| --- | --- | --- | --- |
| ACTB | CTA CCA TGA GCT GCG TGT GG | GTA CAT GGC TGG GGT GTT GA | 130 |
| CD9 | CAT GCT GGG ACT GTT CTT TGG | TAG TGG ATG GCT TTC AGC GT | 183 |
| CDH1 | TCG GAA CTG CAA AGC ACC TG | CAA AAC TCA CTC TGC CCA GGA C | 289 |
| CDX2 | GAG GAC TGG AAT GGC TAC GC | GTC CTG GTT TTC ACT TGG CTG | 332 |
| ELF5 | ACA GCA CCT TCC TGC CTA AT | AGG AGT AAC CTG TCT GAT GCT | 111 |
| FN1 | CCC ATC AGC AGG AAC ACC TT | GTG GGA GCA TCC AGT TTG GT | 140 |
| GATA3 | GTG TGA ACT GCG GAG CAA C | GGT CAG GGG TCT GTT AAT ATT GTG | 278 |
| ITGA1 | CCG AGA TGT GGC CGT AGT TA | ACA CGG TAC TGC AAA TCA GC | 180 |
| ITGA5 | TTA CGG GAC TCA ACT GCA CC | CTC CGG GCA TTT CAG GAT CT | 149 |
| ITGA6 | CAA ATG CAG GCA CTC AGG TTC | TTA TCA GAT GGC TGA GCA TGG A | 226 |
| KRT7 | TCC CAG AAG TCT TTG AGG CCC | GTA GGC AGC ATC CAC ATC CTT C | 206 |
| Mamu-AG | GAC CCC CCC AAG ACA AAT | CAG CCT GAG AGT AGC TCC CGC C | 423 |
| MMP2 | TGG ATG ATG CCT TTG CTC GT | TAT CCG TCA CCA TGC TCC CA | 127 |
| NOTCH1 | GAC GGC ATC AAT GGC TTC AC | CAG TCG CAC TTG TAC CCG TT | 140 |
| NOTCH2 | CCT CTT GGC CTT CCA ACT CCT | ATC TCG ACA CTG CAA GGC TTC T | 165 |
| TCF4 | ATG CAT CAC CAA CAG CGA ATG | ACA GGA GGT GAA AAC ATC GCA | 89 |
| TEAD4 | ACT GGA CAA GCC CAT CGA CAA | TCC TTA GCT GCC TGG TCC TTT | 282 |
| TFAP2C | TGC CTA TGT CTG TGA AGC CG | ATT CTT TAC ACA GTT GCT GGG C | 134 |
| TP63 | TGG AAA CCA GAG ATG GGC AAG | TGT GTG TTC TGA CGA AAC GGG | 135 |
| VIM | ACA AGT CCA AGT TTG CTG ACC | TTG CAG ACG GCC AAT AGT GT | 227 |
| C19MC^*^ | GGT TGG TTT TTT TAT TTG TAA AGT TTT T | CAT CAT AAA ATA TAC ATA C | 449 |
| ELF5^*^ | AAG GGT TGT GTA TAA ATT TGA AAA AT | AAC AAA CAA ATC TCT CTC AAA CAA AC | 342 |

**Supplementary Table S10.** PCR primer sequences

^*^primer designed for bisulfite-converted DNA sequence

**Supplementary Table S11.** Flow cytometry antibodies

| Antibody | Vendor | Catalog Number | Fluorophore |
| --- | --- | --- | --- |
| Cytokeratin 7/8 | BD Biosciences | 564709 | BV421 |
| Vimentin | BioLegend | 677809 | Alexa Fluor 488 |
| Ki-67 | BioLegend | 350515 | BV711 |
| Mamu-AG^1^ (25D3) | Golos lab |  | Alexa Fluor 647 |
| Mamu-E^2^ (MEM-E/06) | Invitrogen | MA1-19356 | PE |
| CD56 | BD Biosciences | 562328 | PE CF594 |
| Live/Dead | Tonbo Biosciences | 13-0865 | Ghost Red 780 |

^1^Slukvin et al. 2000 PNAS

^2^Dambaeva et al. 2008 Placenta

BV: brilliant violet
